# Identification of a viral gene essential for the genome replication of a domesticated endogenous virus in ichneumonid parasitoid wasps

**DOI:** 10.1371/journal.ppat.1011980

**Published:** 2024-04-25

**Authors:** Ange Lorenzi, Fabrice Legeai, Véronique Jouan, Pierre-Alain Girard, Michael R. Strand, Marc Ravallec, Magali Eychenne, Anthony Bretaudeau, Stéphanie Robin, Jeanne Rochefort, Mathilde Villegas, Gaelen R. Burke, Rita Rebollo, Nicolas Nègre, Anne-Nathalie Volkoff

**Affiliations:** 1 DGIMI, Univ Montpellier, INRAE, Montpellier, France; 2 Department of Entomology, University of Georgia, Athens, Georgia, United States of America; 3 INRAE, UMR Institut de Génétique, Environnement et Protection des Plantes (IGEPP), BioInformatics Platform for Agroecosystems Arthropods (BIPAA), Campus Beaulieu, Rennes, France; 4 INRIA, IRISA, GenOuest Core Facility, Campus de Beaulieu, Rennes, France; 5 INRAE, INSA Lyon, BF2I, UMR203, Villeurbanne, France; University of Cambridge, UNITED KINGDOM

## Abstract

Thousands of endoparasitoid wasp species in the families Braconidae and Ichneumonidae harbor "domesticated endogenous viruses" (DEVs) in their genomes. This study focuses on ichneumonid DEVs, named ichnoviruses (IVs). Large quantities of DNA-containing IV virions are produced in ovary calyx cells during the pupal and adult stages of female wasps. Females parasitize host insects by injecting eggs and virions into the body cavity. After injection, virions rapidly infect host cells which is followed by expression of IV genes that promote the successful development of wasp offspring. IV genomes consist of two components: proviral segment loci that serve as templates for circular dsDNAs that are packaged into capsids, and genes from an ancestral virus that produce virions. In this study, we generated a chromosome-scale genome assembly for *Hyposoter didymator* that harbors *H. didymator* ichnovirus (HdIV). We identified a total of 67 HdIV loci that are amplified in calyx cells during the wasp pupal stage. We then focused on an HdIV gene, *U16*, which is transcribed in calyx cells during the initial stages of replication. Sequence analysis indicated that U16 contains a conserved domain in primases from select other viruses. Knockdown of *U16* by RNA interference inhibited virion morphogenesis in calyx cells. Genome-wide analysis indicated *U16* knockdown also inhibited amplification of HdIV loci in calyx cells. Altogether, our results identified several previously unknown HdIV loci, demonstrated that all HdIV loci are amplified in calyx cells during the pupal stage, and showed that U16 is required for amplification and virion morphogenesis.

## Author summary

Parasitoid "domesticated endogenous viruses" (DEVs) provide a fascinating example of eukaryotes acquiring new functions through integration of a viral genome. DEVs consist of multiple loci in the genomes of wasps. Upon activation, these elements collectively orchestrate the production of virions or virus-like particles that are crucial for successful parasitism of host insects. Despite the significance of DEVs for parasitoid biology, the mechanisms regulating key steps in virion morphogenesis are largely unknown. In this study, we focused on the ichneumonid parasitoid *Hyposoter didymator*, which harbors an ichnovirus consisting of 67 proviral loci. Our findings reveal that all proviral loci are simultaneously amplified in ovary calyx cells of female wasps during the pupal stage suggesting the hijacking of cellular replication complexes by viral proteins. We functionally studied the ichnovirus gene *U16*, which encodes a protein with a weakly conserved primase C-terminal domain. Silencing *U16* inhibited viral DNA amplification and virion production, underscoring the key role of this gene for ichnovirus replication. Our study provides evidence that many genes involved in viral DNA replication have been conserved during the domestication of ichnoviruses.

## Introduction

Endogenous viral elements (EVEs) refer to viral sequences in eukaryotic genomes that originate from complete or partial integration of a viral genome into the germline [1]. While retroviruses are the best-known sources of EVEs, bioinformatic studies have also identified non-retroviral EVEs across a diverse range of organisms [2]. Although many EVEs become non-functional and decay through neutral evolution [3], some have been preserved and repurposed by their hosts for new functions, often as short regulatory sequences or individual genes [4,5]. A notable exception to this pattern is observed in domesticated endogenous viruses (DEVs) that have been identified in four lineages of endoparasitoid wasps—insects that lay eggs and develop within the bodies of other insects [6]. Parasitoid DEVs consist of numerous genes conserved within the wasp genome that originate from the integration of complete viral genomes. Unlike other EVEs, these genes remain functional and actively interact to produce viral particles in calyx cells, which are located in the apical part of the oviducts of female wasps [7]. Viral particles are produced in the pupal and adult stages and accumulate in the oviducts of the wasp. Adult female wasps inject these particles along with eggs into insect hosts where they have essential functions in the successful development of wasp offspring [8].

DEVs are most prevalent among species in two wasp families named the Braconidae and Ichneumonidae. The DEVs identified in these two wasp families have evolved from different viral ancestors but through convergence have been similarly repurposed to produce either virions containing circular double-stranded (ds) DNAs or virus-like particles (VLPs) lacking nucleic acid. The hyperdiverse microgastroid complex in the family Braconidae harbors DEVs named bracoviruses (BVs) that evolved from a common virus ancestor in the family *Nudiviridae* [9]. Other braconids in the subfamily Opiinae and ichneumonids in the subfamily Campopleginae independently acquired two other distinct nudiviruses that wasps have coopted to produce VLPs [10,11]. The fourth type of DEV that has been identified are named ichnoviruses (IVs). IVs are present in two ichneumonid subfamilies (Campopleginae and Banchinae) which produce virions containing circular dsDNAs. Unlike the other three DEVs, IVs evolved from a still unknown ancestor. This ancestor was likely though a large DNA virus in the phylum Nucleocytoviricota, which was formerly referred to as nucleocytoplasmic large DNA viruses (NCLDVs) [12,13,14]. Together, BVs and IVs were also earlier referred to as polydnaviruses, but this term is largely no longer used because of strong evidence indicating BVs and IVs derive from different ancestors [6].

BVs have been more studied than IVs but the latter are intriguing because of their uncertain origins. Despite differences in ancestry and gene content, BV and IV genomes are similarly organized into two components that have distinct functions [15]. Insights into the genome components of IVs primarily derive from sequencing two campoplegine wasps named *Hyposoter didymator* and *Campoletis sonorensis* [16], along with calyx transcriptome studies [12,13,17,18] and proteomic analyses of purified virions [12,13]. The first genome component of IVs are domains in the wasp genome that show evidence of deriving from the virus ancestor and having essential functions in virion formation. These domains, named "Ichnovirus Structural Protein Encoding Regions" (IVSPERs), contain intronless genes that are specifically transcribed in calyx cells [12,13,18]. Most IVSPER genes are transcribed at the onset of pupation in hyaline stage 1 pupae [17], and some genes in IVSPERs encode proteins associated with IV virions [12,13]. Six genes have been knocked down by RNA interference (RNAi) in *H*. *didymator* which demonstrated that they have functions in virion assembly or cell trafficking [17]. Five IVSPERs have been identified in the *H*. *didymator* and *C*. *sonorensis* genomes [16], while three have been identified in the genome of the more distantly related banchine *Glypta fumiferanae* [13]. The content of IVSPER genes is notably similar between ichneumonid wasp species [12,13,18], and their gene order is well-conserved among campoplegine species [15]. Additionally, one intronless gene (*U37*) was identified in the *H*. *didymator* and *C*. *sonorensis* genomes outside of any IVSPER with features suggesting it also derives from the IV viral ancestor [16]. Together, these genes, whether found within or outside IVSPERs, represent the fingerprints of the ancestral viral machinery essential for virion production and are designated as IV core replication genes. Notably, none of these genes are packaged in virions, indicating that IV core genes can only be transmitted vertically through the germline of associated parasitoids.

The second component of IV genomes are domains referred to as "proviral segments," which are amplified in calyx cells and produce circular, double-stranded (ds) DNAs that are packaged into capsids [19,20]. The number of proviral segments, typically exceeding 50, are widely dispersed in wasp genomes and exhibit considerable variability between wasp species [16]. Each proviral segment is characterized by flanking direct repeats (DRs) of variable length (<100 bp to >1 kb) and homology that identifies where homologous recombination processes occur to produce circularized, dsDNAs [19, 20]. Some IV proviral segments also contain internal repeats that facilitate additional homologous recombination events and produce multiple overlapping or nested circularized DNAs per proviral segment [16,19]. The circularized dsDNAs produced from proviral segments encode genes with and without introns that are expressed in the hosts of wasps after virion infection [21,22,23,24]. These genes also have functions that are required for the successful development of parasitoid progeny. Thus, while IV core replication genes represent the conserved viral machinery that produces virions in calyx cells, proviral segments contain the IV genome components virions transfer to parasitized hosts.

The replication of IVs, encompassing the processes leading to the production of virions containing IV segments, occurs within the nuclei of calyx cells during pupal and adult developmental stages [7,25]. Electron microscopy studies of *H*. *didymator* ichnovirus (HdIV) show that fusiform-shaped capsids are individually enveloped in the nuclei of calyx cells during the late pupal stage (pigmented pupae, stage 3) [17]. These enveloped "subvirions" exit the nucleus, traverse the cytoplasm, and exit calyx cells by budding, resulting in mature virions with two envelopes that accumulate in the calyx lumen of the ovaries [7, 25]. Earlier findings indicated that IVSPERs and proviral segments undergo amplification in newly emerged adult wasps [12]. However, these data focused on only a subset of IVSPER genes and one proviral segment, leaving our knowledge of whether all IV genome components are amplified in calyx cells incomplete. Similarly, the initiation time of amplification during pupal development and IV virion production remains unknown. The specific role of IV core genes in virion production is also poorly documented when compared to BVs [26,27]. The limited sequence homology of IVSPER genes with genes in other viruses provides minimal insights into potential functions. To date, only the six genes mentioned above that are involved in subvirion assembly or cell trafficking have been studied [17].

In this work, we explored IV DNA replication using the campoplegine wasp *H*. *didymator*. We first generated a chromosome-level assembly for the *H*. *didymator* genome. Through this assembly, we determined that all genome components undergo local amplification in calyx cells which initiates between pupal stages 1 and 2. Notably, IVSPERs, isolated IV core genes, and proviral segments were amplified in large regions with non-discrete boundaries. Next, we studied the function of *U16* which is located on *H*. *didymator* IVSPER-3. *U16* is one of the most transcribed IVSPER genes during the initial pupal stage and contains a weakly conserved domain found in the C-terminus of primases. RNAi knockdown of *U16* inhibited virion formation. Knockdown also significantly reduced DNA amplification of all HdIV genome components, which decreased transcript abundance of IV core genes and the abundance of circular dsDNA viral molecules. We conclude that *U16* is an essential gene for amplification of the HdIV genome and virion production, demonstrating that genes from the IV ancestor regulating IV replication have been conserved during virus domestication. Additionally, our results show that viral DNA amplification is essential for IV virion production.

## Results

### Genomic localization of *Hyposoter didymator* IV components in a novel chromosome-level assembly

The genome assembly for *H*. *didymator* we previously generated [16] consisted of 2,591 scaffolds with an N50 of 4 Mbp. We concluded that this assembly was overly fragmented to evaluate DNA amplification in calyx cells during virion morphogenesis. We therefore used proximity ligation technology to produce a new chromosome-level assembly consisting of twelve large scaffolds that correspond with the haploid karyotype for *H*. *didymator* [28]. The sizes of these scaffolds ranged from 6.7 Mbp to 29.3 Mbp (Fig A and Table A in S1 Dataset).

**Fig 1:**
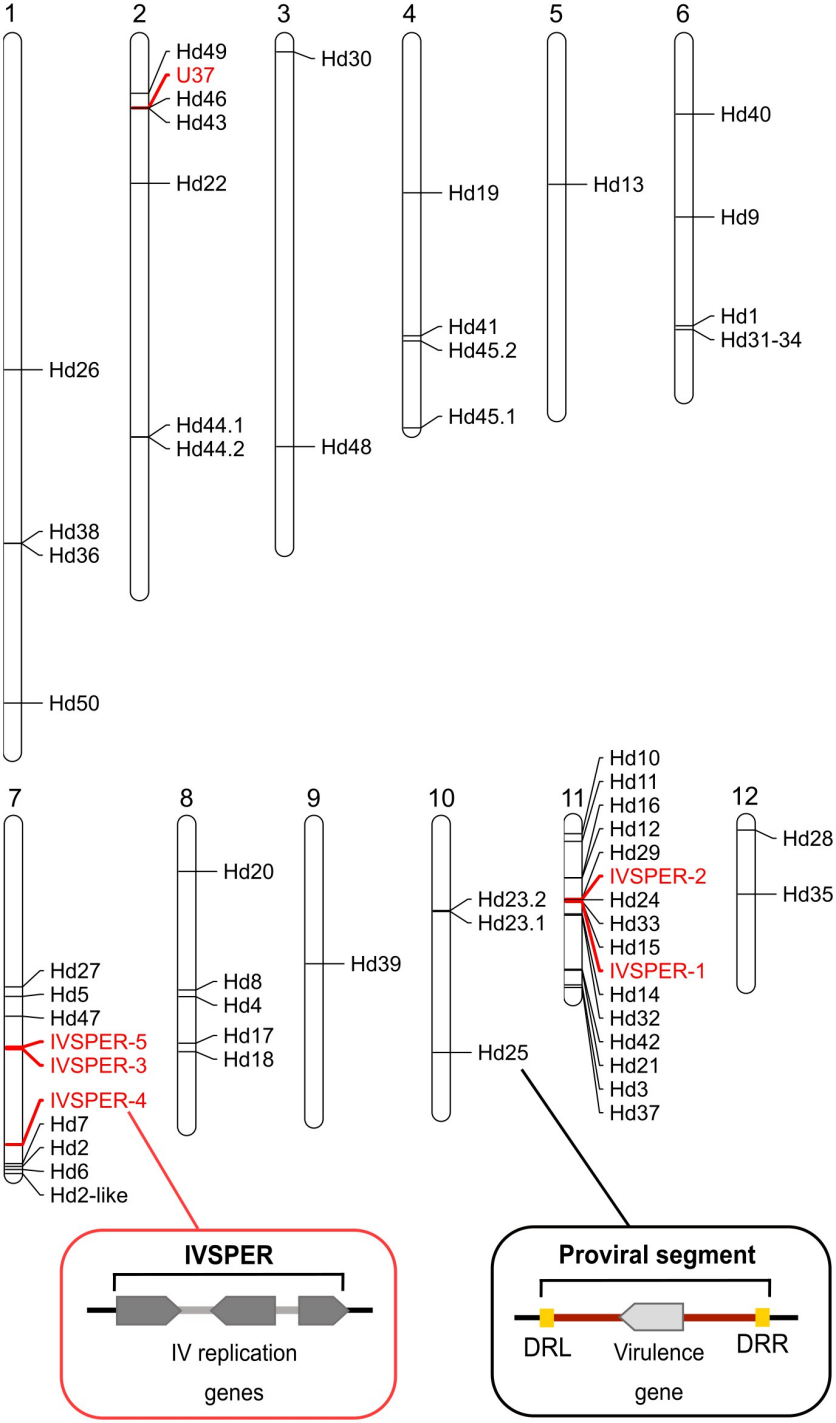
Genome of *H*. *didymator* and position of HdIV loci. Position of the HdIV loci previously identified in [16] within the twelve large scaffolds obtained using Hi-C technology. The HdIV replication genes, organized in Ichnovirus Structural Protein Encoding Regions (IVSPERs) or that are isolated in other regions of the *H*. *didymator* genome, are highlighted in red while viral segments are indicated in black. The scaffold diagram was generated using Mapchart software [29]. At the bottom of the figure, diagrams illustrate the difference between the two types of viral loci: the IVSPER which are clusters of HdIV replication genes inherited from the IV proviral ancestor, and the viral segments flanked by direct repeats located on the left (DRL) and right (DRR) sides of segments, housing virulence genes expressed in the parasitized insect.

The twelve scaffolds contained all HdIV loci previously identified: the replication genes organized in clusters (IVSPER-1 to IVSPER-5), the predicted IV replication gene (*U37*) located outside of an IVSPER, and 53 of the 54 previously identified proviral segments (Hd1 to Hd54) [16] (Fig 1). Only the scaffold containing Hd51 was missing from the 12 main scaffolds, possibly due to low-quality sequencing data in this region (Table A in S1 Dataset). Our chromosome-level assembly revealed that each scaffold contained at least one HdIV locus, but notably, all IVSPERs and 40% of the proviral segment loci resided on two scaffolds (7 and 11) (Fig 1). The high density of HdIV loci observed on scaffold 11 might indicate that this genomic region served as the integration site for the ancestral virus.

While three IVSPERs and the majority of proviral segments were distantly located from each other in the *H*. *didymator* genome, there were exceptions to this pattern including certain pairs of proviral segments separated by less than 20 kb (e.g., Hd36 and Hd38; Hd46 and Hd43; Hd44.1 and Hd44.2; Hd12 and Hd16 in scaffolds -1, -2 and -11 respectively). In all of these cases, the paired segments exhibited significant homology which suggested they derived from recent duplication events (Fig B in S1 Dataset). Additionally, several proviral segments were in proximity to IVSPERs or IV replication genes that resided outside of IVSPERs (e.g., Hd46 near U37; Hd29 and Hd24 on each side of IVSPER-2; Hd15 near IVSPER-1 in scaffolds -2 and -11 respectively; Fig 1).

### Amplification of *Hyposoter didymator* IV genome components in calyx cells during wasp pupal development

**Fig 2:**
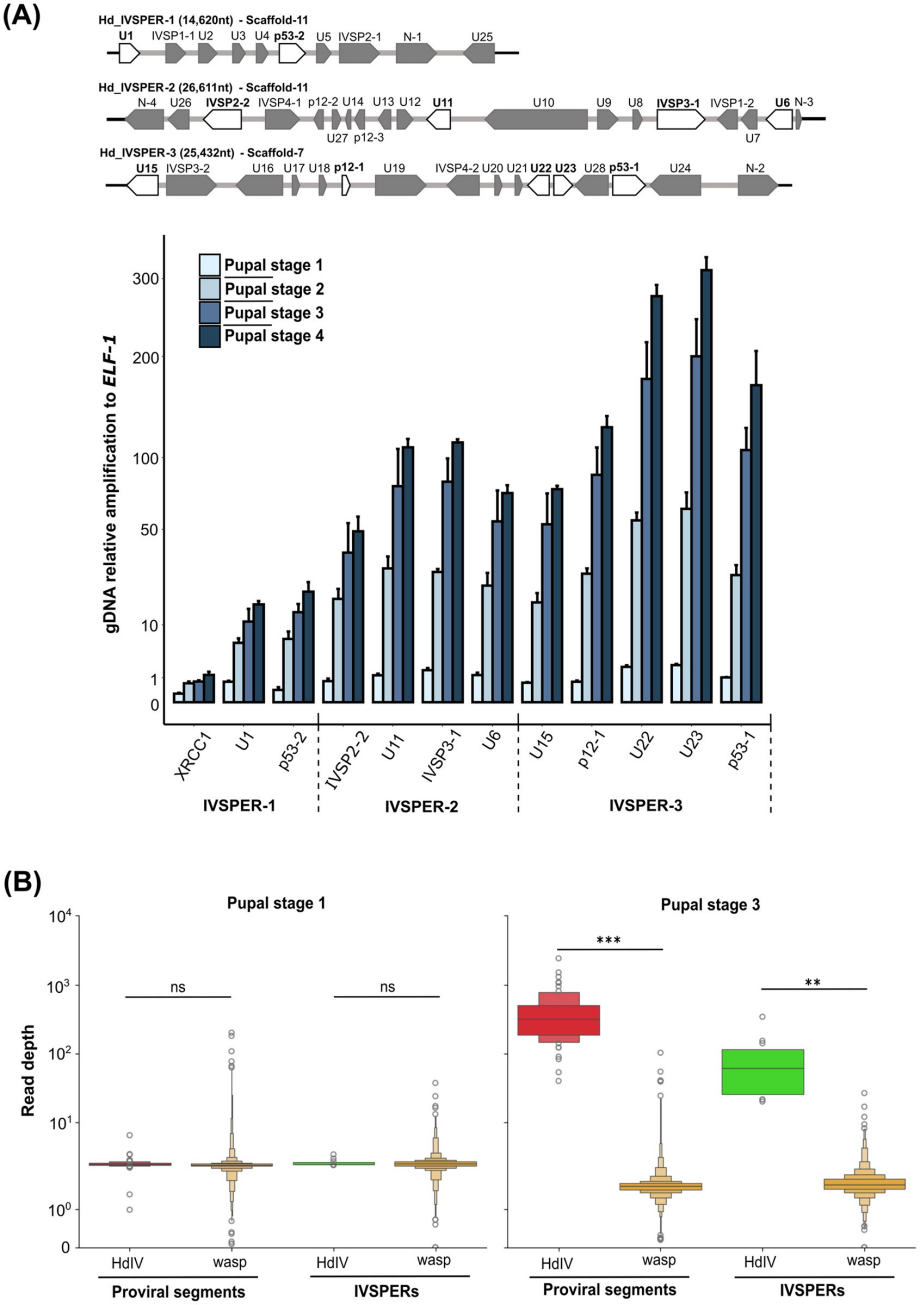
DNA amplification of HdIV loci. **(A)** qPCR analysis of selected IVSPER genes in calyx cells during wasp pupal development. **Top panel**. A schematic representation of *H*. *didymator* IVSPERs-1, -2, and -3 (GenBank GQ923581.1, GQ923582.1, and GQ923583.1); genes selected for qPCR assays are highlighted in white. U1-U24 are unknown protein-encoding genes, while IVSPs are members of a gene family encoding ichnovirus structural proteins. **Bottom panel**. Genomic (g) DNA amplification levels of IVSPER genes and wasp *XRCC1* in calyx cells from pupal stage 1–4. The XRCC1 (X-Ray Repair Cross Complementing 1) encoding gene is located 1,200 bp from *U1* (position 3,270,470 to 3,272,519 in Scaffold-11). Data correspond to gDNA amplification relative to amplification of the housekeeping gene elongation factor 1 (*ELF1*). The Y-axis was transformed using the square root function for better data visualization. Statistical test results are available in Table B in S2 Dataset. **(B)** Read depth of HdIV loci compared to the rest of the wasp genome. Read depth values per analyzed region (see Materials and methods) are presented for each locus type (proviral segments and IVSPERs) at pupal stage 1 (hyaline pupa) and pupal stage 3 (pigmented pupa). The read depths per HdIV locus (HdIV) are compared to the read depth per random genome regions outside of HdIV loci (wasp). Note that the read depth value for random wasp regions is lower for DNA samples collected from stage 3 versus stage 1 pupae. This difference is attributed to the higher proportion of reads mapping to HdIV regions among the total number of reads in stage 3 compared to stage 1. The significance levels are indicated as follows: ns = non-significant, **p<0.01, and ***p<0.001. Statistical test results are available at https://github.com/flegeai/EVE_amplification/blob/main/notebooks/Count_tables.ipynb.

Prior results suggested IV proviral segment loci are amplified before processing and excision of individual segments that are packaged into capsids [19,20]. An earlier study also reported that select IVSPER genes and one proviral segment located near an IVSPER are amplified in *H*. *didymator* adults [12]. However, these findings did not address whether all IV genome components are amplified in calyx cells or when amplification initiates in association with virion formation. To more precisely investigate the temporal dynamics of amplification, we first conducted relative quantitative (q) PCR assays that measured copy number of genes in IVSPER-1, -2, and -3 in calyx DNA samples that were collected from stage 1–4 pupae. We compared these treatments to DNA samples from hind legs of stage 1 pupae where no HdIV replication occurs. We also included a wasp gene (*XRCC1*) located in close proximity to IVSPER-1. Results showed that the copy number of each tested gene was similar in calyx and hind legs in stage 1 pupae (statistical significances in Table B in S2 Dataset, lower panel), indicating none were amplified during the initial pupal stage. Subsequently, the copy number of each gene increased progressively with each pupal stage (Fig 2A and Table B in S2 Dataset). While exhibiting lower amplification levels than the IVSPER genes we analyzed, a similar trend was observed for the wasp gene *XRCC1* (Fig 2A and Table B in S2 Dataset). These findings indicated IVSPER amplification in calyx cells begins between pupal stage 1 (one day old, hyaline) and pupal stage 2, and further increases in pupal stage 3 (five days old, pigmented abdomen) and pupal stage 4.

We next investigated whether all or only some HdIV genome components are amplified by isolating DNA from calyx cells from stage 1 pupae, which the preceding qPCR assays detected no amplification, and stage 3 pupae, which showed high levels of amplication. We then generated paired-end libraries, which were sequenced using the Illumina platform, followed by read alignment to the new chromosome-level genome assembly. When analyzing the reads from stage 1 pupae, read depth per HdIV locus did not differ significantly from read depth of randomly selected regions of the same size from the rest of the wasp genome (Fig 2B). In contrast, for stage 3 pupae, read depth of the HdIV loci was significantly higher than that of randomly selected regions from the rest of the wasp genome (Fig 2A, read depth values in S1 Table), suggesting most or all HdIV loci are amplified in pupal stage 3.

### Differential levels of amplification across all components of the HdIV genome

**Fig 3:**
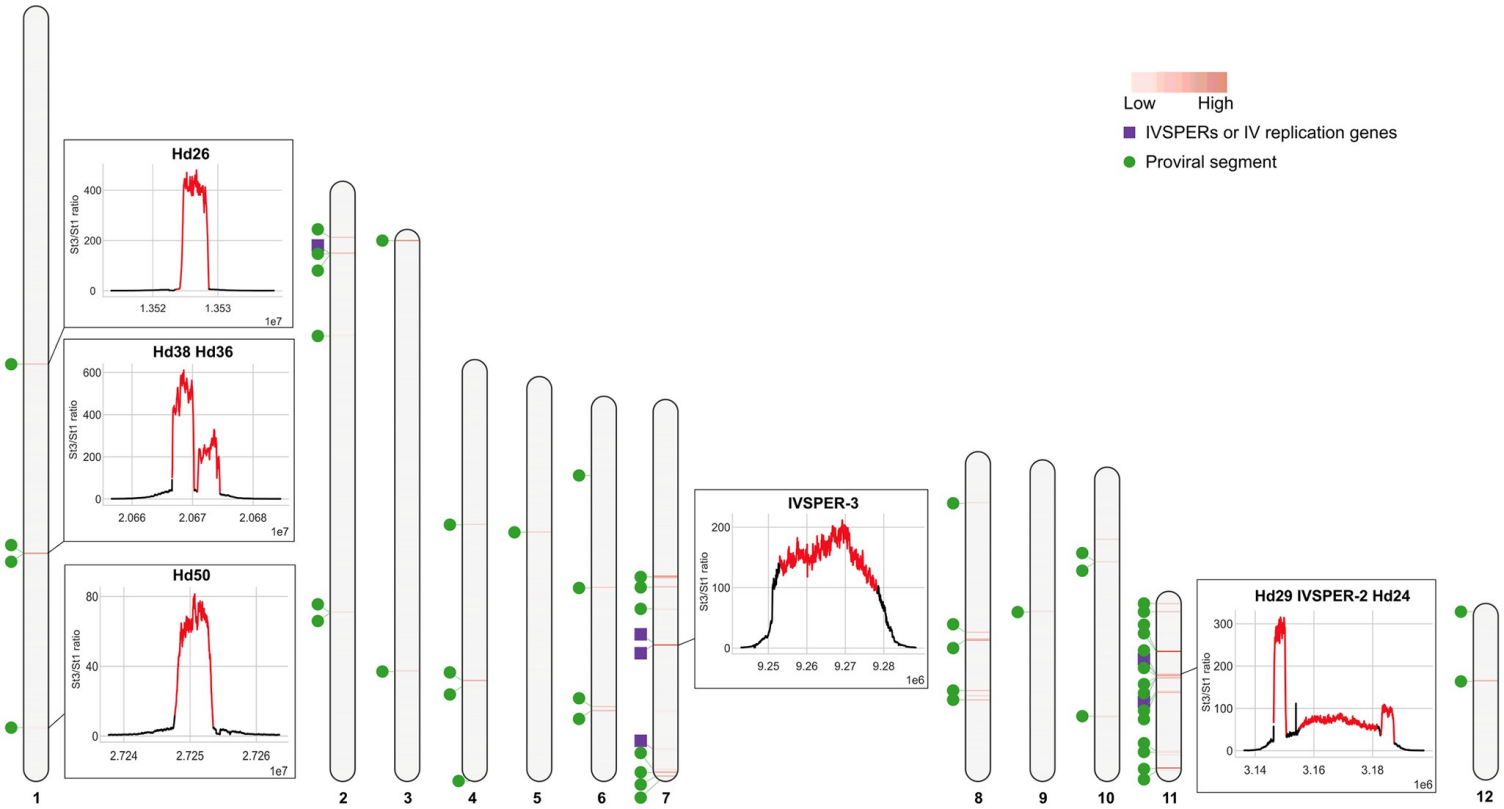
HdIV DNA amplification. DNA amplification in pupal stage 3 was assessed by mapping genomic DNA Illumina reads against the 12 large *H*. *didymator* genome scaffolds. In each scaffold, red bars indicate amplified loci, with the intensity of red corresponding to increased values of the counts per million (CPM) ratio between pupal stage 3 and pupal stage 1. The positions of IVSPERs and isolated IV replication genes are indicated by purple squares, while proviral segments are indicated by green circles. For selected HdIV loci, amplification curves (representing the ratio of the CPM values calculated for 10 bp intervals between pupal stage 3 and pupal stage 1) are shown in boxes. Amplification curves for all of the annotated HdIV loci are shown in S1 Fig. Each HdIV locus is indicated in red while 10,000 bp of flanking sequence on each side of the locus is also shown. For proviral segments, loci are defined as the sequence delimited by two direct repeats; IVSPERs are defined as the region between the start and stop codon of the first and last coding sequences in the cluster; isolated IV replication genes are defined by their coding sequence.

While the preceding results suggested all HdIV genome components were amplified in calyx cells, the qPCR results presented in Fig 2A also indicated amplification levels varied with genes in IVSPER-3 exhibiting higher levels of amplification than genes in IVSPER-1 and -2. We therefore also analyzed read depth per position and the ratio of read depths between stage 3 and stage 1 in our Illumina data set (Figs 3 and S1). This analysis indicated that amplification levels of IVSPER loci, determined at the summit of the read depth curve, ranged from 10X for IVSPER-5 in Scaffold-7 to over 200X for IVSPER-3 in Scaffold-3 (S1 Table). Consistent with the findings from our qPCR analysis, IVSPER-3 was more highly amplified than IVSPER-1 and -2. Results from the read depth per position curve further indicated that the peak of amplification occurs toward the middle of each IVSPER (S1 Fig), consistent with our qPCR data for genes closer to the boundary of an IVSPER that exhibit lower levels of amplification than genes in the middle of the cluster (Figs 2A, 3 and S1).

Proviral segment loci were relatively more amplified than IV replication gene loci, but were also variable (Figs 3 and S1). For example, the ratio of read depth between stage 3 and stage 1 ranged from 30X for proviral locus Hd40 in Scaffold-6 to over 1,100X for Hd27 in Scaffold-7 (S1 Table) at the summit of the read depth curves. Variability in sequencing depth among proviral loci was consistent with earlier studies indicating that the circularized DNAs packaged into IV capsids are non-equimolar in abundance [8,30].

**Fig 4:**
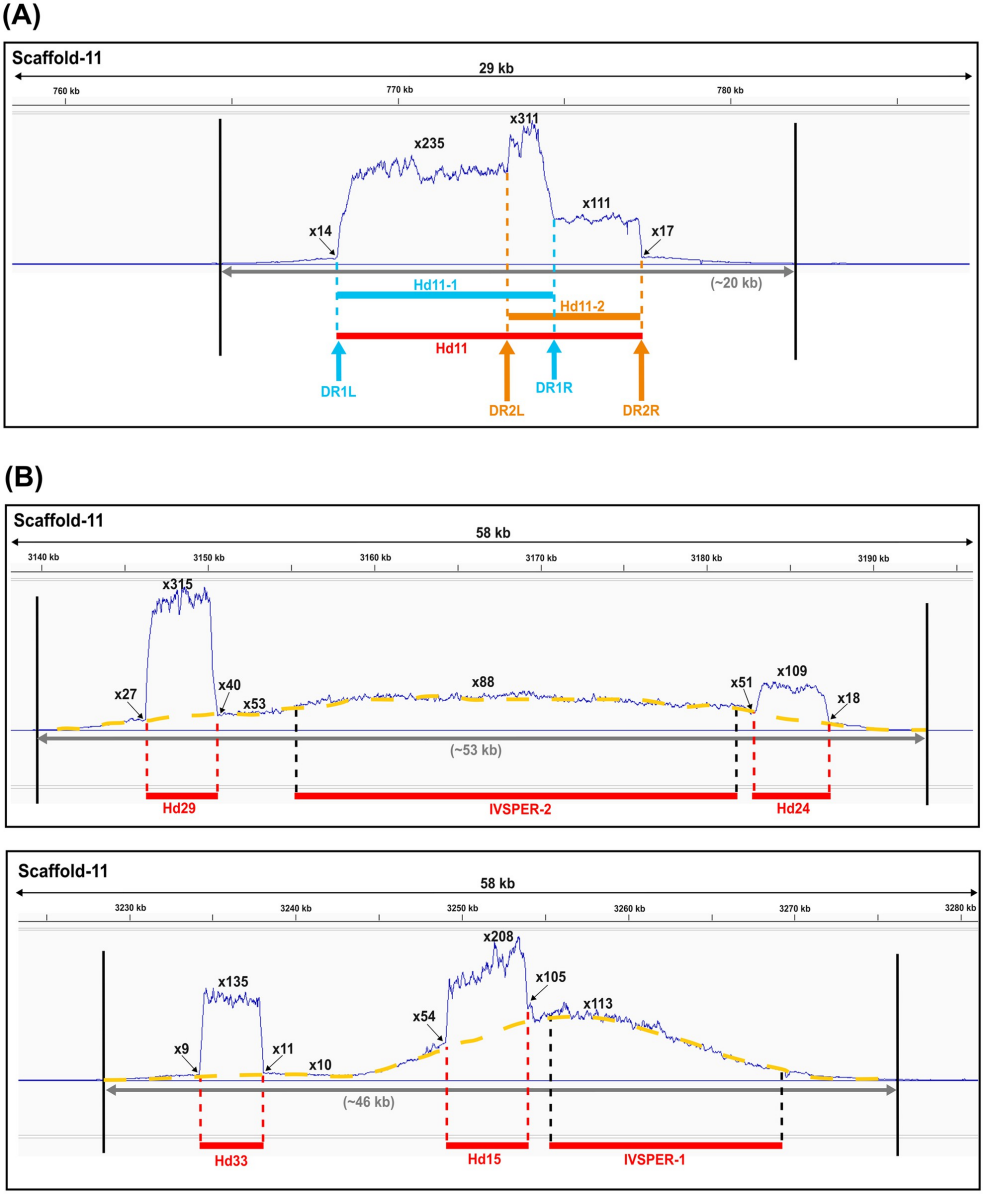
HdIV amplified regions in Scaffold-11. (A) Detail of the amplified region at the Hd11 locus. Hd11 (red bar) represents two overlapping segments Hd11-1 (defined by left and right DRs, DR1L and DR1R respectively, blue bar) and Hd11-2 (defined by DR2L and DR2R, orange bar). **(B)** Detail of two other amplified regions containing IVSPERs and HdIV proviral loci. In **(B)**, amplification curves of IVSPERs are highlighted in yellow. In **(A)** and **(B)**, amplification curves represent the ratio of the CPM values (calculated for 10 bp intervals) obtained in pupal stage 3 compared to pupal stage 1. For each locus, amplification values at the summit of the peaks (bold type) and at the start and end positions of HdIV segments are indicated with arrows. Each amplification curve figure was generated by Integrated Genome Viewer (IGV) [31].

All proviral segments consistently exhibited a substantial increase in amplification that peaked between the two DRs (as exemplified by Hd14 or Hd12 in S2 Fig). For numerous proviral loci, the reads mapping between the flanking DRs displayed uniform read depth. However, in other cases, peaks with varying read depth were evident (as exemplified by Hd32 or Hd16 in S2 Fig). This differential read depth usually applied to proviral segments that contained more than one pair of DRs, as illustrated by proviral locus Hd11 (Fig 4A) or Hd32 and Hd16 (S2 Fig). Previous studies indicated that Hd11 contains two pairs of DRs, enabling the formation of two nested, circularized segments termed Hd11-1 (formed by recombination between DR1Left (DR1L) and DR1Right (DR1R)) and Hd11-2 (formed by recombination between DR2L and DR2R) (Fig 4A). Reads mapping to the Hd11 locus (bounded by DR1L and DR2R) exhibited three relatively uniform plateaus of different values. Two plateaus corresponded to reads mapping to the predicted locations of Hd11-1 (235X) and Hd11-2 (111X), while the central region with higher read depth (311X) corresponded to reads mapping to both nested segments (Fig 4A). This differential read depth would not be expected if reads mapped only to Hd11 chromosomal DNA. Consequently, the pattern of proviral segment amplification suggested part of the read depth values were due to reads arising from amplification intermediates and/or circularized dsDNAs that were also present in our DNA samples. Some amplified HdIV loci contain both an IVSPER and proviral segments. Two of these loci resided on Scaffold-11 (Hd29, IVSPER-2, Hd24, and Hd33, Hd15, IVSPER-1 (Fig 4B)). For these loci, the amplification curves spanned the length of the amplified region (yellow dotted line in Fig 4B) but were interrupted by peaks corresponding to the length of proviral segments. This pattern suggested amplification levels of the chromosomal form of the proviral segments could correspond to levels comparable to the surrounding region and to the IVSPER amplification curves, but were higher because of reads also mapping to circular dsDNAs or amplification intermediates.

### *H. didymator* IV genome components are amplified in wasp genome domains with undefined boundaries

Since our read depth data indicated amplified regions were larger than the annotated HdIV loci (Figs 3 and S1), we used the MACS2 peak calling program, originally developed for chromatin immunoprecipitation sequencing experiments, to identify areas in the *H*. *didymator* genome that were enriched for reads when compared to a control [32]. Amplification peaks were called with MACS2 using alignments from stage 3 pupae as the treatment and alignments from stage 1 pupae as the control. MACS2 identified a total of 165 regions that were enriched for reads (i.e. were amplified) (S2 Table). All HdIV genome components that we had annotated in our earlier study [16] were included in these regions. Among the other regions identified by MACS2, manual curation (see Materials and methods section) indicated three were proviral segment loci that we named Hd52, Hd53, and Hd54. Five others contained intronless genes with features suggesting origins from the IV ancestor that were outside of IVSPERs. We thus named these potential IV replication genes *U38*, *U39*, *U40*, *U41*, and *U42*. The remaining regions detected by MACS2 either contained predicted wasp genes or lacked any features that identified them as IV replication genes or proviral segments. Altogether, the MACS2 algorithm predicted a total of 55 regions in the *H*. *didymator* genome containing HdIV loci. Two proviral segments (Hd45.1 on Scaffold-4 and Hd2-like on Scaffold-7) escaped MACS2 detection, possibly because they were located too close to the ends of each scaffold. However, our read mapping data clearly indicated these two segments are amplified in stage 3 (Table 1) with a profile similar to other proviral segments (S1 Fig). In total, read mapping and MACS2 peaks indicated the *H*. *didymator* genome contains 67 HdIV loci (56 proviral segments, five IVSPERs, and six predicted IV replication genes that reside outside of IVSPERs) that are all amplified in calyx cells at pupal stage 3 and embedded in a total of 57 large and locally amplified regions (Fig 3 and Table 1).

**Table 1:** HdIV loci amplified in calyx cells from stage 3 pupae identified by read mapping and/or the MACS2 algorithm. For each scaffold, the position and size of the HdIV loci are indicated. Loci newly identified in the present work are marked with asterisks. Corresponding amplified regions (i.e., the peak predicted by the MACS2 algorithm) are provided for each locus or groups of loci. Start and end positions delimiting the HdIV loci and the amplified regions detected by MACS2 are indicated. The distance between the start or the end of the amplified region and the locus is presented. For each HdIV locus and amplified region detected by MACS2, read depth values are provided for calyx cell samples collected from stage 1 or stage 3 pupae. Read depth is based on the length of the HdIV locus or the amplified region. ND indicates amplified regions not detected by MACS2.

	Annotated proviral locus						Amplified region (MACS2 peak prediction)							
Scaffold	HdIV locus	Locus start	Locus end	Locus size (bp)	Read depth Pupal stage 1	Read depth Pupal stage 3	MACS2 Peak name	Predicted region start	Distance from locus start (bp)	Predicted region end	Distance from locus end (bp)	Region size (bp)	Read depth Pupal stage 1	Read depth Pupal stage 3
Scaffold-1	Hd26	13,523,617	13,528,634	5,017	3	723	Hd26	13,517,317	6,300	13,534,392	5,758	17,075	2	217
	Hd38	20,666,612	20,670,275	3,663	3	1,090	Hd38+Hd36	20,656,417	10,195	20,681,709	11,434	25,292	2	246
	Hd36	20,670,791	20,674,528	3,737	3	501			14,374		7,181			
	Hd50	27,247,704	27,253,490	5,786	2	123	Hd50	27,241,046	6,658	27,259,533	6,043	18,487	2	42
Scaffold-2	Hd49	2,124,324	2,129,588	5,264	2	505	Hd49	2,117,165	7,159	2,135,110	5,522	17,945	2	155
	U37	2,735,040	2,736,878	1,838	3	88	U37+Hd46+Hd43	2,728,207	6,833	2,758,008	21,130	29,801	3	132
	Hd46	2,737,536	2,741,644	4,108	2	188			9,329		16,364			
	Hd43	2,744,162	2,748,320	4,158	3	365			15,955		9,688			
	Hd22	5,839,467	5,843,644	4,177	2	152	Hd22	5,832,207	7,260	5,850,065	6,421	17,858	2	40
	Hd44.1	16,284,645	16,287,653	3,008	3	218	Hd44.1+Hd44.2	16,278,981	5,664	16,302,690	15,037	23,709	2	60
	Hd44.2	16,289,605	16,294,435	4,830	3	122			10,624		8,255			
Scaffold-3	Hd30	419,458	423,621	4,163	3	1,502	Hd30	410,434	9,024	429,813	6,192	19,379	3	336
	Hd48	16,689,745	16,699,417	9,672	3	184	Hd48	16,679,121	10,624	16,706,535	7,118	27,414	2	70
Scaffold-4	Hd19	6,231,095	6,235,534	4,439	3	428	Hd19	6,229,498	1,597	6,243,248	7,714	13,750	2	143
	Hd41	12,119,882	12,127,834	7,952	2	618	Hd41	12,113,372	6,510	12,135,096	7,262	21,724	2	233
	Hd45.2	12,329,917	12,331,967	2,050	1	83	Hd45.2	12,321,418	8,499	12,340,715	8,748	19,297	2	18
	Hd45.1	15,910,208	15,914,421	4,213	1	186		ND	ND	ND	ND	ND	ND	ND
Scaffold-5	Hd13	5,876,265	5,882,021	5,756	3	385	Hd13	5,869,105	7,160	5,889,886	7,865	20,781	2	112
Scaffold-6	Hd40	2,988,772	2,992,265	3,493	2	54	Hd40	2,983,699	5,073	2,997,266	5,001	13,567	2	16
	Hd9	7,229,774	7,247,665	17,891	2	148	Hd9	7,223,110	6,664	7,254,609	6,944	31,499	3	87
	U42*	9,084,542	9,085,630	1,088	3	20	U42*	9,075,600	8,942	9,095,671	10,041	20,071	2	8
	Hd1	11,708,977	11,723,747	14,770	7	284	Hd1	11,702,950	6,027	11,728,752	5,005	25,802	5	168
	Hd31-34	11,868,292	11,872,410	4,118	3	945	Hd31-34	11,862,430	5,862	11,878,832	6,422	16,402	2	242
Scaffold-7	U38*	4,768,418	4,770,145	1,727	2	30	U38*	4,758,477	9,941	4,776,797	6,652	18,320	2	15
	Hd27	6,697,648	6,701,649	4,001	3	2,407	Hd27	6,688,177	9,471	6,708,677	7,028	20,500	2	479
	Hd52*	6,772,399	6,776,469	4,070	2	141	Hd52*	6,765,613	6,786	6,783,734	7,265	18,121	2	35
	Hd5	7,084,945	7,098,657	13,712	2	220	Hd5	7,078,958	5,987	7,104,504	5,847	25,546	2	124
	Hd47	7,900,815	7,905,317	4,502	3	248	Hd47	7,894,307	6,508	7,912,018	6,701	17,711	2	67
	IVSPER-5	9,178,274	9,179,903	1,629	3	22	IVSPER-5	9,168,108	10,166	9,191,030	11,127	22,922	2	9
	IVSPER-3	9,252,903	9,278,334	25,431	2	342	IVSPER-3	9,243,166	9,737	9,287,918	9,584	44,752	2	221
	U39*	11,747,266	11,750,520	3,254	3	92	U39*	11,738,062	9,204	11,764,025	13,505	25,963	3	33
	IVSPER-4	13,191,258	13,207,069	15,811	2	61	IVSPER-4	13,176,550	14,708	13,216,107	9,038	39,557	3	33
	Hd7	13,968,918	13,976,983	8,065	2	157	Hd7	13,965,551	3,367	13,978,603	1,620	13,052	2	98
	Hd2	14,085,672	14,099,608	13,936	3	373	Hd2	14,079,432	6,240	14,106,161	6,553	26,729	2	205
	Hd6	14,221,056	14,231,516	10,460	3	473	Hd6	14,218,171	2,885	14,238,167	6,651	19,996	3	254
	Hd2-like	14,388,343	14,389,879	1,536	3	528		ND	ND	ND	ND	ND	ND	ND
Scaffold-8	Hd20	1,942,407	1,949,270	6,863	2	189	Hd20	1,934,960	7,447	1,956,552	7,282	21,592	2	65
	Hd8	6,823,745	6,831,100	7,355	2	404	Hd8	6,818,165	5,580	6,837,954	6,854	19,789	2	156
	Hd4	7,097,009	7,107,334	10,325	2	780	Hd4	7,090,110	6,899	7,114,242	6,908	24,132	2	340
	Hd17	9,012,106	9,019,835	7,729	2	342	Hd17	9,006,318	5,788	9,026,009	6,174	19,691	2	138
	Hd54*	9,214,207	9,220,125	5,918	2	319	Hd54*	9,208,814	5,393	9,228,042	7,917	19,228	2	104
	Hd18	9,364,794	9,369,489	4,695	2	771	Hd18	9,360,612	4,182	9,376,778	7,289	16,166	2	229
Scaffold-9	U40*	5,723,866	5,725,782	1,916	3	37	U40+Hd39	5,714,059	9,807	5,757,032	31,250	42,973	2	37
	Hd39	5,744,955	5,749,076	4,121	2	243			30,896		7,956			
	U41*	7,049,476	7,051,946	2,470	2	22	U41	7,040,265	9,211	7,060,018	8,072	19,753	2	11
Scaffold-10	Hd23.2	3,553,203	3,556,564	3,361	3	90	Hd23.2	3,545,352	7,851	3,561,495	4,931	16,143	3	27
	Hd23.1	3,592,527	3,596,983	4,456	2	229	Hd23.1	3,587,203	5,324	3,605,012	8,029	17,809	2	63
	Hd25	9,399,172	9,403,345	4,173	3	496	Hd25	9,393,559	5,613	9,412,007	8,662	18,448	2	117
Scaffold-11	Hd10	453,966	460,472	6,506	2	403	Hd10	447,712	6,254	465,323	4,851	17,611	2	154
	Hd11	768,148	777,337	9,189	2	361	Hd11	764,311	3,837	783,916	6,579	19,605	4	175
	Hd16	2,262,941	2,270,644	7,703	2	1,298	Hd16+Hd12	2,257,264	5,677	2,292,899	22,255	35,635	2	318
	Hd12	2,280,031	2,285,932	5,901	2	177			22,767		6,967			
	Hd29	3,146,273	3,150,628	4,355	3	587	Hd29+IVSPER-2+Hd24	3,139,131	7,142	3,193,136	42,508	54,005	2	153
	IVSPER-2	3,155,228	3,181,838	26,610	2	153			16,097		11,298			
	Hd24	3,182,748	3,187,444	4,696	3	196			3,128,743		5,692			
	Hd33	3,234,245	3,238,079	3,834	3	244	Hd33+Hd15+IVSPER-1	3,229,681	4,564	3,277,203	39,124	47,522	2	115
	Hd15	3,249,051	3,254,037	4,986	3	363			19,370		23,166			
	IVSPER-1	3,255,266	3,269,285	14,019	2	141			25,585		7,918			
	Hd14	3,759,577	3,764,772	5,195	2	325	Hd14	3,753,870	5,707	3,770,377	5,605	16,507	2	107
	Hd32	3,811,943	3,819,858	7,915	2	402	Hd32	3,807,216	4,727	3,823,379	3,521	16,163	2	200
	Hd42	6,043,764	6,046,920	3,156	2	184	Hd42	6,039,490	4,274	6,052,307	5,387	12,817	2	48
	Hd21	6,076,348	6,080,715	4,367	2	312	Hd21	6,067,122	9,226	6,086,904	6,189	19,782	2	75
	Hd53*	6,184,778	6,190,126	5,348	3	178	Hd53*	6,177,710	7,068	6,199,505	9,379	21,795	2	49
	Hd3	6,685,111	6,695,124	10,013	2	807	Hd3	6,678,585	6,526	6,701,155	6,031	22,570	2	367
	Hd37	6,786,206	6,789,913	3,707	2	220	Hd37	6,779,687	6,519	6,795,693	5,780	16,006	2	55
Scaffold-12	Hd28	308,843	313,456	4,613	2	40	Hd28	305,632	3,211	316,324	2,868	10,692	2	19
	Hd35	2,941,876	2,945,585	3,709	3	1,062	Hd35	2,935,430	6,446	2,954,029	8,444	18,599	2	219

Our results also indicated all amplified regions in the *H*. *didymator* genome containing HdIV loci consist of the annotated HdIV locus along with flanking wasp sequences consistent with our detailed analysis of the wasp gene *XRCC1* that is located in close proximity to IVSPER-1 (Fig 2B). Across all HdIV loci, we determined that the flanking regions containing wasp sequences that were amplified varied from 7,000 to 15,000 bp (Table 1). The total size of the amplified regions ranged from 10,692 bp (Hd28 on Scaffold-12) to 54,005 bp (IVSPER-2 on Scaffold-11). Most amplified regions contained a single HdIV locus, but seven contained a mix of HdIV genome components (Table 1). Three amplified regions contained the neighboring and closely related proviral segments mentioned above (e.g., Hd36 and Hd38 on Scaffold-1, Hd44.1 and Hd44.2 on Scaffold-2, Hd12 and Hd16 on Scaffold-11). In addition to the two examples noted above on Scaffold 11 (see Fig 4B), two other amplified regions also contained both IVSPERs and proviral segments (*U37*, Hd46, and Hd43 on Scaffold-2; *U40* and Hd39 on Scaffold-9). The simultaneous amplification of both types of HdIV loci within the same amplicons suggested regulation by shared molecular mechanisms. In turn, we further hypothesized similar molecular mechanisms also regulate the amplification of isolated proviral segments and IVSPERs.

Lastly, we searched for sequence signatures that potentially identify the amplification boundaries for each HdIV locus. However, our analysis identified only low-complexity A-tract sequences, which were not specific to HdIV components as they were also found in random non-amplified wasp genomic sequences (S3 Fig). Thus, no motifs were identified that distinguished the amplification boundaries of HdIV loci.

### RNAi knockdown of *U16* inhibits virion morphogenesis

**Fig 5:**
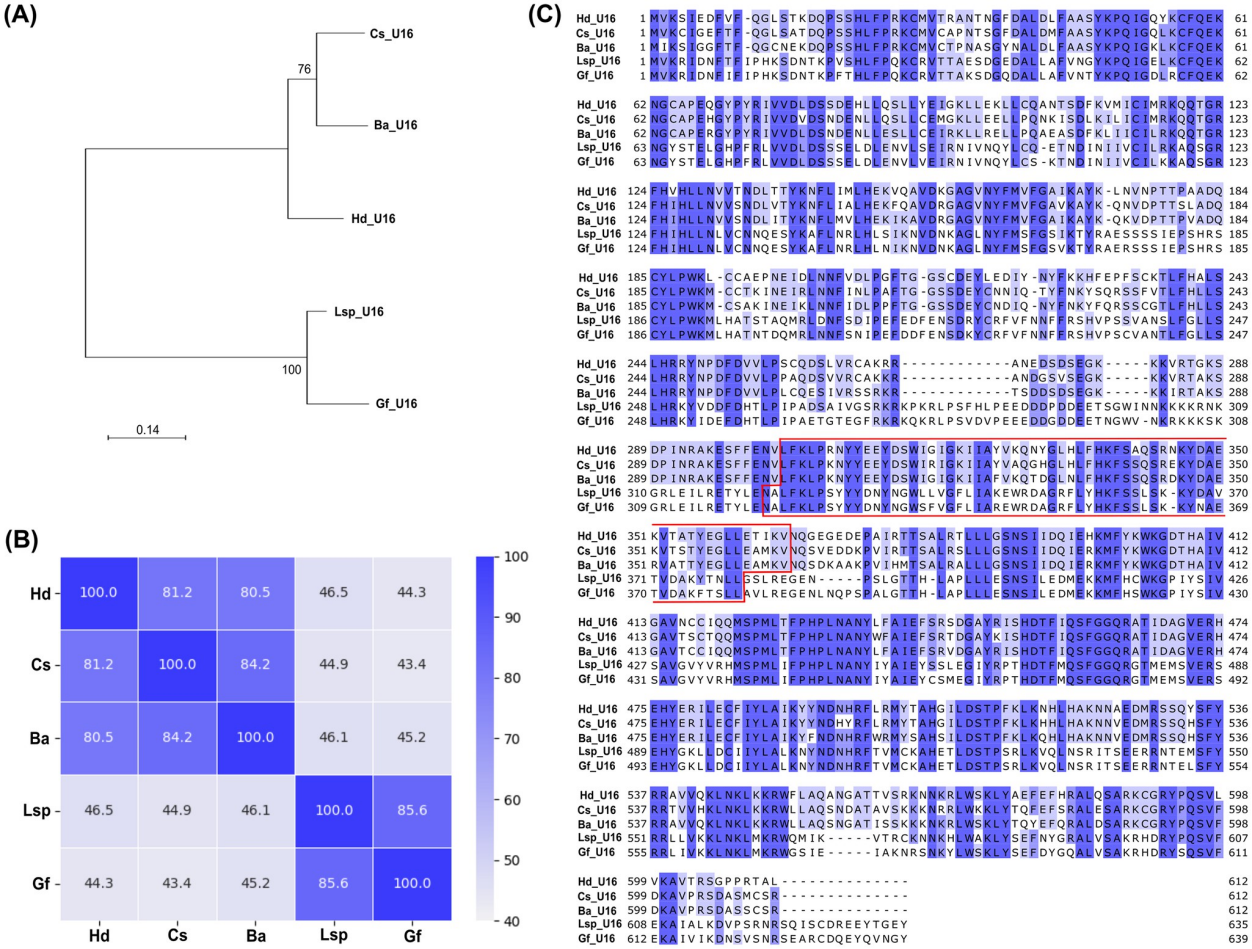
U16 proteins analysis. U16 proteins were identified in the campoplegine *Hyposoter didymator* [12], *Campoletis sonorensis* [16], and *Bathyplectes anurus* [18], and in two banchine wasps *Glypta fumiferanae* [13] and *Lissonota* sp. [34]. **(A)** Phylogenetic tree of U16 proteins inferred using a maximum-likelihood method. Branch supports are labeled near the nodes. **(B)** Identity heat map of U16 proteins. **(C)** Alignment of U16 proteins. The predicted PriCT-2 domain is indicated by the red outline. In panels (B) and (C), increased blue intensity depicts a stronger degree of identity between the proteins. Abbreviations: Hd, *H*. *didymator*; Cs, *C*. *sonorensis*; Ba, *B*. *anurus*; Gf, *G*. *fumiferanae*; Lsp, *Lissonota* sp.

**Fig 6:**
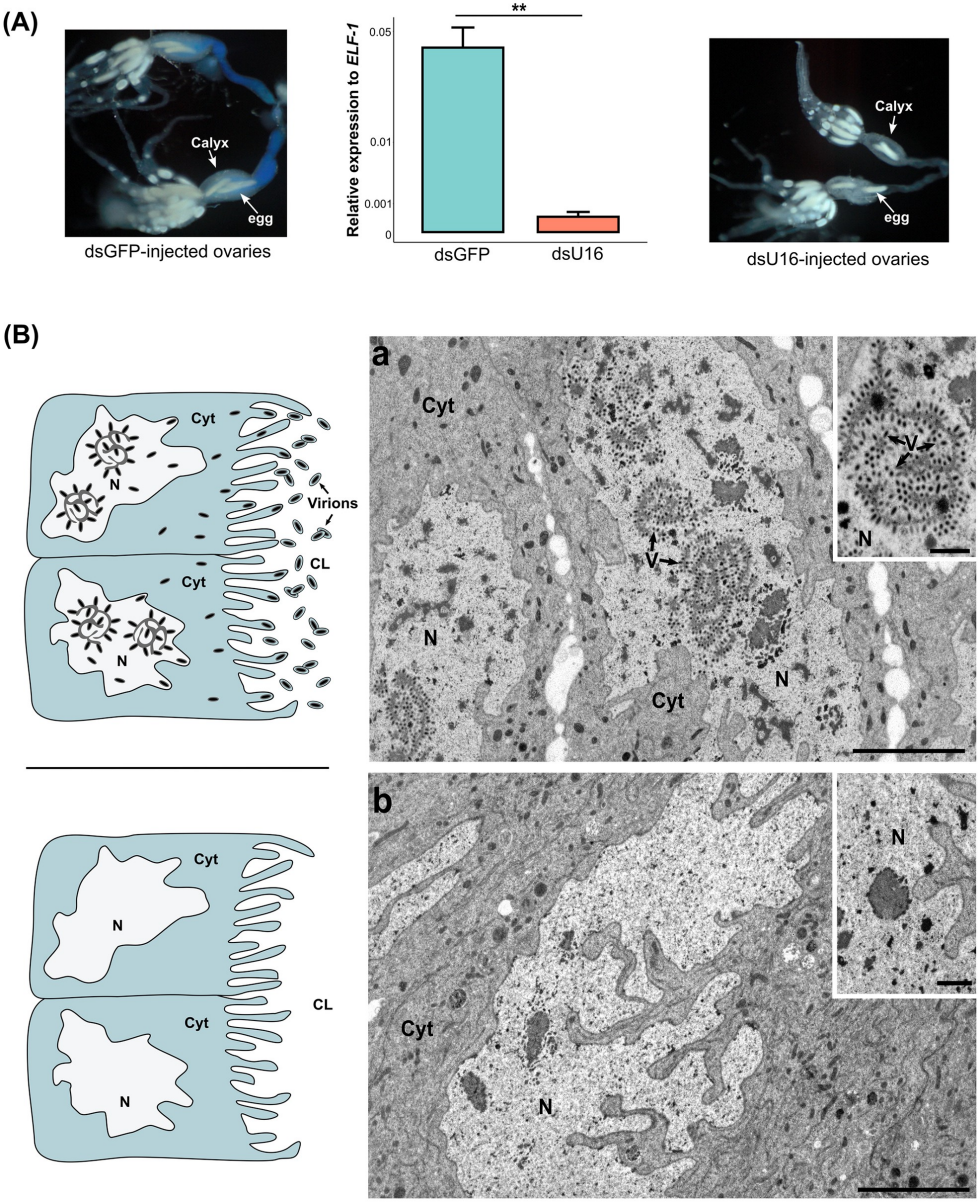
RNAi knockdown of *U16*. **(A)** RT-qPCR data showing relative expression of *U16* in ds*GFP* (control) and ds*U16* injected females. ** p<0.01. Images of ovaries dissected from newly emerged adult females that were injected with ds*GFP* (left) or ds*U16* (right). Note the blue color in the oviduct of the ds*GFP* control indicating the presence of HdIV virions. **(B)** Schematics and electron micrographs showing that (a) calyx cell nuclei (N) from females treated with ds*GFP*-injected contain subvirions (V) while (b) calyx cell from a ds*U16*-injected wasps do not. This results in no accumulation of virions in the calyx lumen as illustrated in the schematic images. Abbreviations: CL, calyx lumen; Cyt, cytoplasm. Scale bars = 5 μm, zooms = 1 μm.

We selected the gene *U16* located on *H*. *didymator* IVSPER-3 as a factor with potential functions in activating IV replication. *U16* is conserved among all IV-producing wasps for which genome or transcriptome data are available (Fig 5). In *H*. *didymator* calyx cells, *U16* is also one of the most transcribed IV genes detected in calyx cells from stage 1 pupae [16]. Sequence analysis using the basic local alignment search tool and DeepLoc2.0 predicted all U16 family members contain a C-terminal alpha-helical domain (PriCT-2) of unknown function that is present in several primases [33] and a nuclear localization signal (Fig 5 and S3 Dataset). We next assessed the effects of knocking down *U16* by RNAi on virion morphogenesis in calyx cells. We injected newly pupated wasps with dsRNAs that specifically targeted *U16* using previously established methods [17]. RT-qPCR analysis indicated transcript abundance in the calyx of newly emerged adult females was reduced by more than 90% when compared to control wasps that were injected with ds*GFP* (Fig 6A). Inspection of the ovaries further indicated that the calyx lumen of control wasps contained blue ‘calyx fluid’ indicative of HdIV virions being present, whereas almost no calyx fluid was seen in ds*U16*-injected wasps (Fig 6A). Examination of calyx cell nuclei by transmission electron microscopy similarly showed that calyx cells in one-day-old control females contained an abundance of subvirions, whereas no subvirions were observed in treatment wasps (Fig 6B). We thus concluded that U16 is required for virion morphogenesis.

### RNAi knockdown of *U16* also disables amplification of HdIV loci

**Fig 7:**
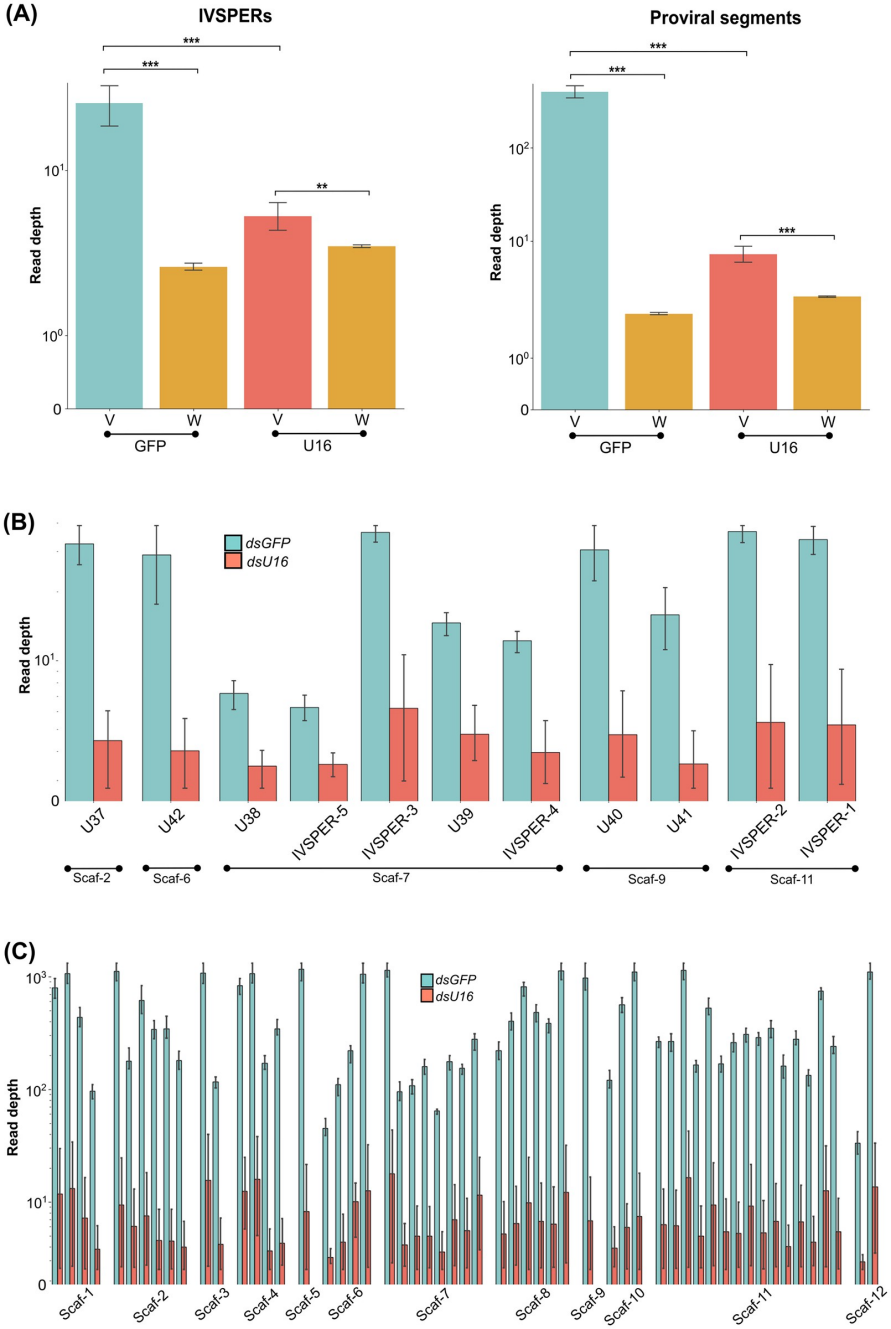
Impact of *U16* RNAi knockdown on DNA proviral amplification. **(A)** Comparative distribution of read depths in ds*GFP*- and ds*U16*-injected females. Read depth values in three biological replicates are given for HdIV loci (V) and random genomic regions outside of HdIV loci (W) with equal size distribution. IVSPERs and IV replication genes loci are shown in the left panel, while proviral segment loci are shown in the right panel. **(B)** Read depth values for IVSPERs and IV replication genes residing outside an IVSPER in three biological replicates treated with ds*U16*- or ds*GFP*. Names of HdIV loci and the scaffold (Scaf-) they are located are indicated. **(C)** Read depth values for proviral segment loci in the three biological replicates of the ds*U16* and ds*GFP* samples. For better visualization, only the scaffold (Scaf-) on which the proviral segments are located is indicated. The list of the proviral segment loci within each scaffold is available in Table 1. The y-axis was transformed by the log function for better data visualization. In **(A)**, significance levels are indicated as follows: **p<0.01, and ***p<0.001. In **(B)** and **(C)**, all differences between ds*U16* and ds*GFP* samples are statistically significant at p<0.05. Statistical test results are available at https://github.com/flegeai/EVE_amplification/blob/main/notebooks/Count_tables.ipynb.

Since *U16* contained a domain found in primases, we investigated whether RNAi knockdown also disabled amplification of HdIV genome components. We injected newly pupated wasps with ds*U16* or ds*GFP*, followed by isolation and deep sequencing of calyx cell DNA from stage 3 pupae in three independent replicates. Mapping the reads from ds*GFP*-treated calyx samples to the *H*. *didymator* genome indicated all HdIV loci were amplified as evidenced by higher read depth values when compared to random regions of the wasp genome (Fig 7A). Silencing *U16* significantly decreased read depth values when compared to ds*GFP* samples for both IVSPER and proviral segment loci (Fig 7A). However, read depth values remained higher for HdIV loci compared to other regions of the wasp genome in ds*U16*-treated samples (Fig 7A), suggesting that ds*U16* treatment did not completely abolish *U16* expression across the three replicates. Upon individual analysis of each HdIV locus, we observed that read depth for every HdIV genome component (including IVSPERs, isolated IV replication genes, or HdIV proviral segments) was lower in the ds*U16*-treated samples compared to the ds*GFP*-treated samples (Fig 7B and 7C and S3 Table; statistical analyses at https://github.com/flegeai/EVE_amplification).

**Fig 8:**
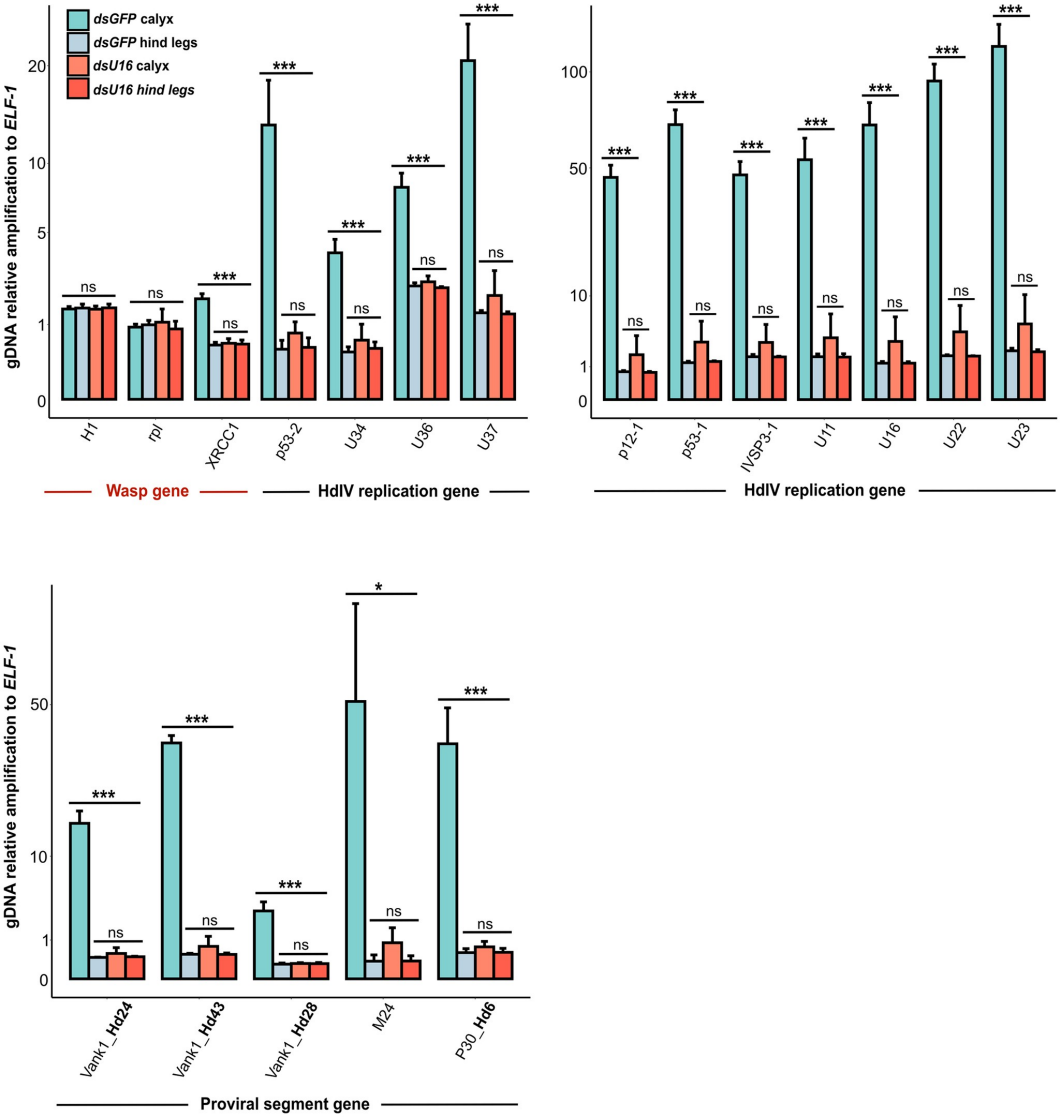
Impact of *U16* RNAi knockdown on amplification of selected wasp and HdIV genes. Relative genomic amplification of select HdIV genes in two-day-old females injected with ds*GFP* or ds*U16*. The wasp gene *XRCC1*, located within the amplified region of the IVSPER-1 locus, was incorporated into the analysis. Wasp histone (*H1*) and ribosomal protein (*rpl*) genes served as controls. Samples were obtained from calyx cells (where virion are produced) and hind legs (control). Statistical significance levels are denoted as follows: ns = non-significant, *p<0.05, **p<0.01, and ***p<0.001. Statistical test results are available in Table D in S2 Dataset. The y-axis values were transformed using the square root function for better data visualization.

We extended our analysis by injecting ds*GFP* or ds*U16* into newly formed pupae followed by isolation of DNA from calyx cells and hind legs where no HdIV replication occurs. We then used specific primers and qPCR assays that measured DNA abundance of three wasp genes, selected HdIV replication genes inside and outside of IVSPERs, and selected HdIV genes in different proviral segments. As anticipated, no genes were amplified in hind legs from either control or treatment wasps (Fig 8 and Table D in S2 Dataset). In ds*GFP*-injected control wasps, all HdIV genes were amplified in calyx cell samples (Fig 8 and Table D in S2 Dataset). Among the wasp genes, only *XRCC1* exhibited significant amplification, consistent with its location within the IVSPER-1 amplified region (Fig 8 and Table D in S2 Dataset). In contrast, when examining calyx cell DNA from wasps injected with ds*U16*, none of the HdIV genes nor *XRCC1* were amplified (Fig 8 and Table D in S2 Dataset). Altogether, our results indicate that U16 is required for amplification of all HdIV loci.

### Impact of DNA amplification on IV replication gene transcription levels and abundance of circularized HdIV molecules in calyx cells

**Fig 9:**
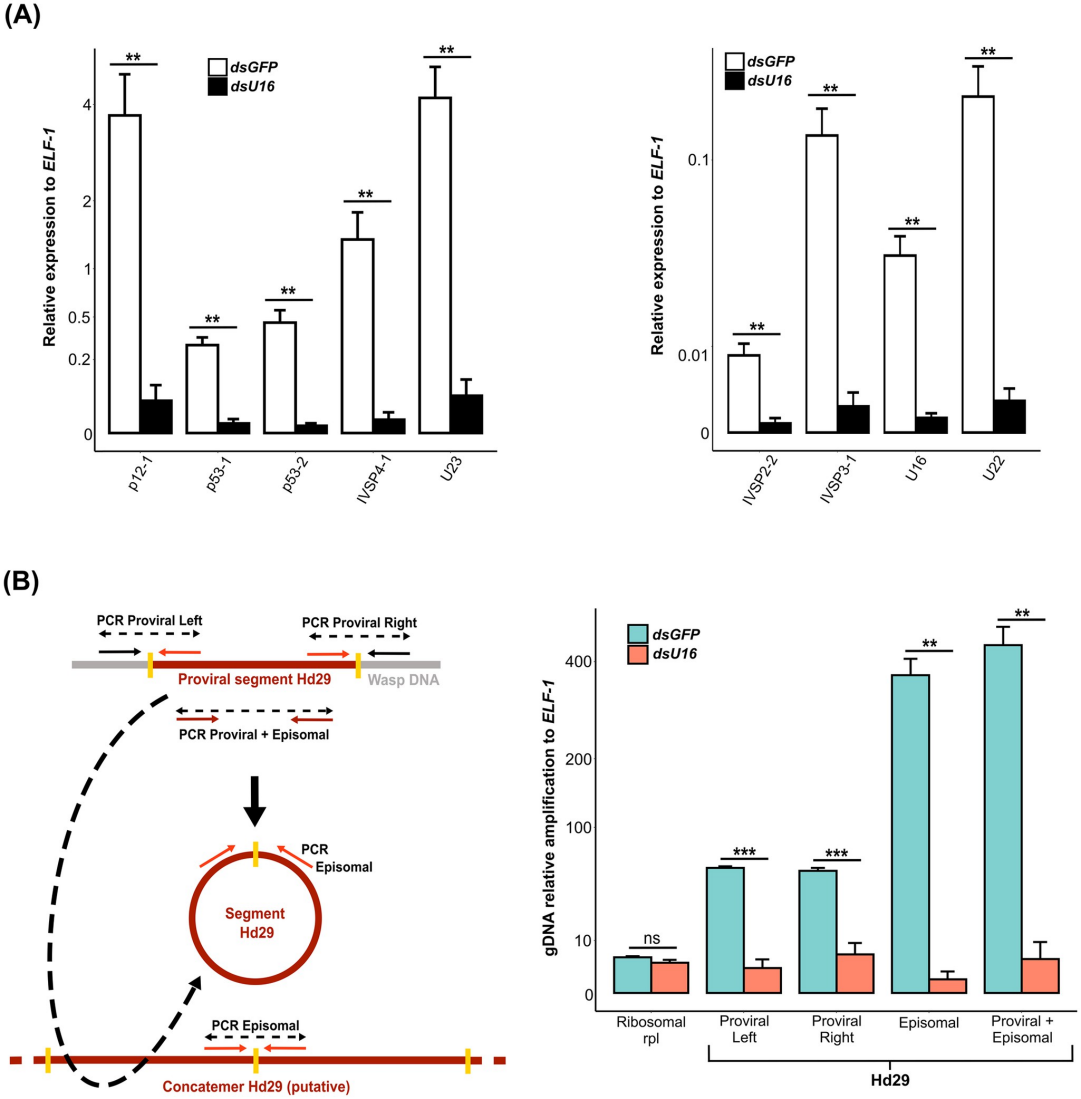
Impact of *U16* RNAi knockdown on HdIV replication gene expression and proviral segment amplification. **(A)** Relative expression of nine IVSPER genes in 2-day-old adult females injected with ds*GFP* (control) or ds*U16*. **(B)** Relative DNA amplification of the integrated linear (proviral) and circularized (episomal) forms of viral segment Hd29 in 2-day-old adult females injected with ds*GFP* (control) or ds*U16*. The left panel illustrates the position of primer pairs designed to selectively amplify the proviral form (Proviral Left and Right, indicated by red and black arrows), the circularized form (Episomal, red arrows), or both (Proviral + Episomal, brown arrows). Note that the primers designed to amplify circular molecules may also anneal to concatemers generated during segment amplification and processing. The right panel presents the relative amplification of each form using DNA from ds*GFP*- and ds*U16*-injected females. In both **(A)** and **(B)**, significance levels are indicated as follows: ns = non-significant, *p<0.005, **p<0.01, and ***p<0.001. Statistical test results are available in Tables F (A) and H (B) in S2 Dataset. The y-axis values were transformed using the square root function for better data visualization.

We hypothesized that amplification of IV replication genes would increase transcript abundance which in turn would be affected by inhibiting HdIV DNA amplification. We thus compared transcript abundance of various genes in IVSPER-1, -2, and -3, in calyx RNA samples that were collected from wasps treated with ds*U16* or ds*GFP*. *U16* knockdown reduced the expression of every HdIV replication gene we examined (Fig 9A and Table F in S2 Dataset).

Finally, we investigated the impact of *U16* knockdown on the abundance of the circularized dsDNAs that are processed from amplified proviral segments. For this assay, we used PCR primers that specifically amplified the proviral form, circularized (episomal) or concatemeric form or both forms of Hd29 (Fig 9B and Table H in S2 Dataset). Results showed a significant reduction in both the proviral and circularized/concatemeric forms of Hd29 in calyx cell DNA from wasps injected with ds*U16* when compared to DNA from wasps injected with ds*GFP* (Fig 9B and Table H in S2 Dataset). Our results thus indicated U16 is required for proviral segment amplification which is also required for production of circularized segments.

## Discussion

This study significantly advances our understanding of IV replication by generating a chromosome-level assembly for the *H*. *didymator* genome, presenting several lines of evidence on the timing of HdIV loci amplification in calyx cells, and identifying *U16* as an essential gene for amplification of all HdIV loci and virion formation. Read mapping to genomic DNA extracted from *H*. *didymator* pupal stages 1 and 3 further shows that all HdIV genome components are concurrently amplified in calyx cells while our qPCR data indicate amplification begins between pupal stage 1 and stage 2 and reaches its maximum in stage 4. This temporal pattern is overall similar to what has previously been shown to occur for BVs [27,35]. In the braconid wasp *Chelonus inanitus*, DNA content of calyx cells also increases through endoreduplication [36], which we have not yet examined in *H*. *didymator* but is a future goal. Overall, our results indicate that DNA amplification of HdIV genome components in calyx cells is an essential feature of virion morphogenesis in *H*. *didymator*.

A second important advance from this study is our finding that HdIV loci are amplified with non-discrete boundaries that extend variable distances into flanking wasp DNA. This result differs from other integrated viruses such as polyomavirus where DNA undergoes “onion skin" replication, which is distinguished by replication forks that terminate at discrete boundaries [37]. In contrast, IVSPER amplification does share similarities with chorion genes in *Drosophila* follicle cells that are amplified in regions that extend ~100 kb beyond the genes themselves without discrete termination boundaries [38,39]. Similar to IVSPERs, levels of DNA amplification in *Drosophila* follicle cells also vary between amplicons [39,40]. Amplification of chorion gene loci is associated with multiple origins of replication (ORs) that are interspersed within each gene cluster. This results in overlapping bidirectional replication forks progressing outward on either side of the ORs [40]. Thus, IVSPERs and IV replication genes may also contain multiple ORs but additional approaches, such as nascent strand sequencing based on λ-exonuclease enrichment [41], will be necessary to determine if this is the case.

Our study also highlights that amplification of proviral segment loci is associated with a significant increase in read depth at the Direct Repeat (DR) positions bordering the proviral segments, which serve as sites for homologous recombination and circularization of the segments. This suggests that a portion of the rapid increase in read depth is due to reads mapping to amplification intermediates and circularized segments. The presence of concatemeric intermediates and circularized HdIV DNAs in the genomic DNA samples we sequenced is supported by our qPCR results for segment Hd29 (Fig 9B). Quantifying the proportion of reads mapping to the chromosomal form of HdIV segments was not possible in this study because our paired-end read data cannot discriminate between chromosomal HdIV DNA, potential replication intermediates, or circularized DNA. Long-read data as generated for some BVs [42] will be required to identify HdIV replication intermediates. Nonetheless, our results overall suggest HdIV proviral segment amplification involves both local chromosomal amplification and amplification of intermediates related to producing the circular dsDNAs that are packaged into capsids.

Our interest in *U16* stemmed from previous results indicating it is transcriptionally upregulated in calyx cells before the appearance of envelope and capsid components [17]. Sequence analysis during this study revealed a PriCT-2 domain in U16, known from primases in herpesviruses, whose function is unknown but may facilitate the association of the large primase domain (AEP) with DNA [33,43]. Although other known primase domains were not identified in the U16 sequence, the presence of a PriCT-2 domain suggested this protein might play a role in the replication of HdIV genome components. Additionally, our RNAi experiments demonstrate that *U16* knockdown resulted in the complete absence of virion production in calyx cell nuclei and calyx fluid. These observations indicated an essential role for U16 in the early stages of viral replication, potentially involved in the amplification of HdIV genome components and/or the transcriptional regulation of IV replication genes. Subsequently, we analyzed the genome-wide impact of RNAi knockdown of *U16* on HdIV loci amplification, revealing that this gene is crucial for the amplification of all *H*. *didymator* IV genome components. In the case of IV replication genes, reduced amplification was accompanied by a simultaneous significant reduction in transcript abundance, likely resulting in insufficient amounts of HdIV structural proteins. However, amplification and transcription abundance levels did not fully correlate with each other. For instance, *U11* and *IVSP3-1* (both located on IVSPER-2) exhibit similar amplification patterns (Fig 2), but earlier findings showed that transcript abundances were not the same in calyx cells [16]. Thus, differences in gene expression observed among genes located within the same amplified regions (Fig 2) could also be affected by promoter strength or other factors. On the other hand, inhibition of proviral segment loci amplification had consequences for the abundance of the circularized dsDNA that is packaged into capsids, which were drastically reduced. Thus, our results identify U16 as an essential protein for virion morphogenesis. However, its precise role in viral replication remains to be understood. Questions to be addressed in the future include whether U16 acts at the initiation or elongation step of HdIV DNA replication, whether it interacts directly with DNA, or with proteins from the replisome complex, which itself could be composed of a mixture of HdIV and wasp proteins.

BVs share some features with IVs but also exhibit differences. Notably, in contrast to IVs, where most core genes with functions in virion morphogenesis reside in IVSPERs, many BV core replication genes are widely dispersed in the genomes of wasps [44,45,46] and are not amplified in calyx cells during virion morphogenesis [41]. However, some BV-producing wasp genomes contain a ~400 Kb DNA domain housing several nudiviral core genes, known as the nudivirus-like cluster, potentially marking the integration site of the nudivirus ancestor of BVs in the common ancestor of microgastroid braconids [9]. Notably, the nudivirus-like cluster is amplified with non-discrete boundaries [42], similar to what is reported for IV genome components in this study. The observed similarity in the amplification pattern between the BV nudivirus cluster and the proviral components of IVs could suggest they are amplified through a common mechanism, even though the specific genes involved differ.

BV genomes also contain proviral segment loci with boundaries defined by flanking DRs and amplified in regions that include flanking regions outside of each DR. However, unlike IV proviral segments, the amplified flanking regions in BVs contain very precise nucleotide junctions that identify the boundaries of amplification [42,47]. It is also known that some BV proviral segments are amplified as head-to-tail concatemers, consistent with a rolling circle amplification mechanism, while others are amplified as head-to-head and tail-to-tail concatemers, suggesting amplification by different mechanisms. However, all of these concatemers are similarly processed into circular DNAs by recombination at a precise site within DRs, which is a tetramer conserved in all BV segments [42,47]. Nudiviral genes encoding tyrosine recombinases are further known to mediate this homologous recombination event [26,27]. These types of molecules could also be present in IV genomes and need to be discovered. Currently, a detailed comparison between BV and IV proviral segment amplification is challenging and will require more information about the machinery involved in the processing of IV proviral segments into circular dsDNAs that are packaged into capsids.

Collectively, our results identify *U16* as a gene deriving from the IV ancestor that is required for HdIV DNA replication. This indicates that viral regulatory factors required for DNA amplification other than U16 have been preserved in parasitoid genomes, which may also interact with wasp cellular machinery in regulating viral replication. Furthermore, this work emphasizes the value of studying original endogenized viruses, such as those found in parasitoids, to unveil new regulators of DNA processing.

## Materials and methods

### Insects

*H*. *didymator* was reared as previously outlined by [48]. Female pupae obtained from cocoons were staged using pigmentation patterns: stage 1, corresponding to hyaline pupae (approximately 3-day-old pupae); stage 2, had a pigmented thorax (4-day-old); stage 3, had a pigmented thorax and abdomen (5-day-old); stage 4, were pharate adults just before emergence.

### Dovetail Omni-C Library preparation and sequencing

DNA from 10 male offspring (i.e., haploid genomes) from a single female *H*. *didymator* was sent on dry ice to Dovetail Genomics for Omni-C library construction. In the process of constructing the Dovetail Omni-C library, chromatin was fixed in place within the nucleus using formaldehyde and subsequently extracted. The fixed chromatin was digested with DNAse I followed by repair of chromatin ends and ligation to a biotinylated bridge adapter. Proximity ligation of adapter-containing ends ensued. Post-proximity ligation, crosslinks were reversed, and the DNA was purified. The purified DNA underwent treatment to eliminate biotin not internal to ligated fragments. Sequencing libraries were generated utilizing NEBNext Ultra enzymes and Illumina-compatible adapters. Fragments containing biotin were isolated using streptavidin beads before PCR enrichment of each library. The library was sequenced using the Illumina HiSeqX platform, which generated approximately 30x read depth. Subsequently, HiRise utilized reads with a mapping quality greater than 50 (MQ>50) for scaffolding purposes.

### Scaffolding the assembly with HiRise

The *de novo* assembly from [16], and the Dovetail OmniC library reads served as input data for HiRise, a specialized software pipeline designed for leveraging proximity ligation data to scaffold genome assemblies, as outlined by [49]. The sequences from the Dovetail OmniC library were aligned to the initial draft assembly using the bwa tool (available at https://github.com/lh3/bwa). HiRise then analyzed the separations of Dovetail OmniC read pairs mapped within the draft scaffolds. This analysis generated a likelihood model for the genomic distance between read pairs. The model was subsequently employed to identify and rectify putative misjoins, score potential joins, and execute joins above a specified threshold. A contact map was generated from a BAM file by utilizing read pairs where both ends were aligned with a mapping quality of 60.

### Genomic DNA (gDNA) extraction for high throughput sequencing

*Comparative analysis of two pupal stages*. Genomic DNA (gDNA) was extracted from pooled calyx samples dissected from *H*. *didymator* female pupae at stage 1 (~60 females) and stage 3 (~50 females). Since the aim was to compare the two developmental pupal stages, a single replicate was done for each stage. *Impact of U16 knockdown*. Genomic DNA from calyces was collected from stage 3 female pupae that were injected with ds*GFP* and ds*U16*. This experiment involved three biological replicates, each corresponding to 30 to 50 calyx samples. Genomic DNA was extracted using the phenol-chloroform method. Briefly, calyx samples were incubated in proteinase K (Ambion, 0.5 μg/μl) and Sarkosyl detergent (Sigma, 20%), followed by treatment with RNAse (Promega, 0.3 μg/μl). Total genomic DNA was then extracted through phenol-chloroform extraction and ethanol precipitation. Following extraction, gDNA was quantified using a QBIT fluorometer (ThermoFisher) and subsequently sent for sequencing to Genewiz/Azenta company. Paired-end sequencing was carried out using Illumina technology and NovaSeq 2x150bp platform.

### NGS data analyses

Illumina reads were aligned to the updated version of the *H*. *didymator* genome using bwa mem [50], version 0.7.17, with default parameters. Subsequently, the aligned reads were converted to BAM files utilizing samtools view (version 1.15) [51].

#### Prediction of the amplified regions

Amplification peaks were identified using MACS2 [31] by comparing the pupal stage 3 alignment file as treatment and the pupal stage 1 alignment file as control. The specified parameters for this analysis were:—broad—nomodel -g 1.8e8 -q 0.01—min-length 5000. Out of the 165 predicted peaks (i.e., amplified regions), only those with a fold change (FC) higher than 2 were retained for further analyses, resulting in a total of 59 peaks. These 59 peaks encompassed all known proviral loci, except for Hd40, which had a slightly lower value than the specified threshold (FC = 1.9), and Hd45.1 and Hd2-like, located too close to the scaffold end and potentially missed. For the predicted peaks with FC>2 that did not correspond to known proviral loci, a manual curation was performed to determine whether these regions corresponded to HdIV loci. Proviral segments were identified by their flanking direct repeats (DRs) and gene contents, specifically the presence of genes belonging to IV segment conserved gene families. To identify putative core IV replication genes, genes present in the MACS2 peak were analyzed. Only those with no similarity to wasp proteins and that were transcribed in calyx cells (based on the available transcriptome from [17]) were retained.

#### Comparison of read depths between HdIV loci and the rest of the wasp genome

Read depths were computed for various types of virus-related genomic regions, including each locus (IVSPERs, IV replication genes outside IVSPERs, proviral segments), and each MACS2-detected amplified region. To calculate read depth for non-viral regions of the wasp genome, one hundred sets of random regions mimicking the size distribution of HdIV loci were generated using the shuffle tool from bedtools version 2.27 [52]. This was achieved by utilizing the bed file of HdIV loci (56 for proviral segments and 11 for IVSPERs) as parameters for the shuffle tool. Raw read counts were determined for each proviral and wasp genomic region using featureCounts [53] from the Subread package (version 2.0.1) with the parameters (-c -P -s 0 -O). Subsequently, read depth values were computed for each pupal stage (stage 1, St1 and stage 3, St3), as well as for each experiment (ds*GFP* and ds*U16*) and each replicate with a custom script available at https://github.com/flegeai/EVE_amplification. Read depth values for each region were calculated by dividing the number of fragments mapped to the region by the size of the region (expressed in kilobase pairs, kbp), and further normalized by the depth of the library (expressed in million reads).

#### Genome read depths per position on *H. didymator* scaffolds (Counts per Million, CPM) and Maximal value of amplification per proviral locus

Genome read depths per position in 10 bp bins were acquired using the BamCoverage tool from the deeptools package [54] with the options:—normalizeUsing CPM and -bs 10. Subsequently, for each 10 bp bin, the pupal stage 3 (St3) versus stage 1 (St1) ratio was computed through an in-house script available at https://github.com/flegeai/EVE_amplification. This script utilized the pyBigWig python library from deeptools [54]. To determine the maximal counts per million (CPM) at each stage for every proviral locus, an in-house script importing the pyBigWig python library was employed. The maximum CPM value for the "stage 3 / stage 1" ratio was then calculated based on the 10 bp bin bigwig file, specifically for the position displaying the highest CPM value at stage 3 (summit).

#### Search for motifs at the HdIV amplified regions boundaries

The MEME suite [55] was employed for analyses using default parameters and a search for six motifs. A dataset comprising a total of 110 sequences, each spanning 1,000 nucleotides on both sides of the start and end positions of the 55 HdIV amplified regions predicted by the MACS2 algorithm, was utilized for this analysis. As a control, a parallel analysis was conducted using 110 sequences, each 2,000 nucleotides in length, randomly selected from locations within the *H*. *didymator* genome but outside the HdIV loci. This control dataset allowed for the comparison of motif patterns between the HdIV amplified regions and randomly chosen genomic regions.

### Genomic DNA extraction for gDNA amplification analyses by quantitative real-time PCR

To assess the level of DNA amplification, total genomic DNA (gDNA) was extracted using the DNeasy Blood & Tissue Kit (Qiagen) following the manufacturer’s protocol. Ovaries (ovarioles removed) and hind legs, representing the negative control, were dissected from ten pupae at four different stages. Three replicates were generated for each pupal stage. Quantification of target gene amplification was conducted through quantitative PCR, utilizing LightCycler 480 SYBR Green I Master Mix (Roche) in 384-well plates (Roche). The total reaction volume per well was 3 μl, comprising 1.75 μl of the reaction mix (1.49 μl SYBR Green I Master Mix, 0.1 μl nuclease-free water, and 0.16 μl diluted primer), and 1.25 μl of each gDNA sample diluted to achieve a concentration of 1.2 ng/μl. Primers used are listed in S4 Table. The gDNA levels corresponding to the viral genes and the housekeeping wasp gene (elongation factor (ELF-1)) were determined using the LightCycler 480 System (Roche). The cycling conditions involved heating at 95°C for 10 min, followed by 45 cycles of 95°C for 10 s, 58°C for 10 s, and 72°C for 10 s. Each sample was evaluated in triplicate. The obtained DNA levels were normalized with respect to the wasp gene ELF-1. Raw data are provided in S2 Dataset.

### Total RNA extraction

Total RNA was extracted from ovaries (ovarioles removed) dissected from pupae at different stages using the Qiagen RNeasy extraction kit in accordance with the manufacturer’s protocol. To control for gene silencing, total RNAs were also extracted from individual adult wasp abdomens (2 to 4 days old). For this, Trizol reagent (Ambion) was initially used followed by extraction using the NucleoSpin RNA kit (Macherey-Nagel). Isolated RNA was then subjected to DNase treatment using the TURBO DNA-free Kit (Life Technologies) to assure removal of any residual genomic DNA from the RNA samples.

### Protein sequence analyses

Conserved domains of U16 were identified using the CD-search tool available through NCBI’s conserved domain database resource [56,57]. Subcellular localization predictions were made using the DeepLoc—2.0 tool, a deep learning-based approach for predicting the subcellular localization of eukaryotic proteins [58]. Protein multiple sequence alignment and phylogenetic tree were generated using MEGA11 software [59], using MUSCLE for protein alignment and Maximum Likelihood method to generate the phylogenetic tree. The alignment was visualized using Jalview [60]. Structure predictions for U16 were carried out using the MPI Bioinformatics Toolkit [61].

### RNA interference (RNAi)

Gene-specific double-stranded RNA (dsRNA) used for RNAi experiments was prepared using the T7 RiboMAX Express RNAi System (Promega). Initially, a 350–450 bp fragment corresponding to the *U16* sequence was cloned into the double T7 vector L4440 (a gift from Andrew Fire, Addgene plasmid # 1654). Subsequently, an *in vitro* transcription template DNA was PCR amplified with a T7 primer, and this template was used to synthesize sense and antisense RNA strands with T7 RNA polymerase at 37°C for 5 hours. The primers used for dsRNA production are listed in S4 Table. After annealing and DNase treatment using the TURBO DNA-free Kit (Life Technologies), the purified dsRNAs were resuspended in nuclease-free water, quantified using a NanoDrop ND-1000 Spectrophotometer (Thermo Scientific), and examined by agarose gel electrophoresis to ensure their integrity. Injections were performed in less than one-day-old female pupae using a microinjector (Fentojet Express, Eppendorf) and a micromanipulator (Narishige). Approximately 0.3–0.6 μl of 500 ng/μl dsRNA was injected into each individual. Control wasps were injected with a non-specific dsRNA homologous to the green fluorescent protein (GFP) gene. Treated pupae were kept in an incubator until adult emergence, which occurred approximately 5 days after injection.

### Transmission electron microscopy

Ovaries were dissected from adult wasps between 2 and 3 days after emergence, following the procedures outlined in [17]. To ensure consistency of the observed phenotype, at least three females (taken at different microinjection dates) were observed for each tested dsRNA. For transmission electron microscopy (TEM) observations, calyces were fixed in a solution of 2% glutaraldehyde in PBS for 2 hours and then post-fixed in 2% osmium tetroxide in the same buffer for 1 hour. Tissues were subsequently bulk-stained for 2 hours in a 5% aqueous uranyl acetate solution, dehydrated in ethanol, and embedded in EM812 resin (EMS). Ultrathin sections were double-stained with Uranyless (DeltaMicroscopy) and lead citrate before examination under a Jeol 1200 EXII electron microscope at 100 kV (MEA Platform, University of Montpellier). Images were captured with an EMSIS Olympus Quemesa 11 Megapixels camera and analyzed using ImageJ software [62].

### Reverse-transcriptase quantitative real-time PCR (RT-qPCR)

For RT-qPCR assays, 400 ng of total RNA was reverse-transcribed using the SuperScript III Reverse Transcriptase kit (Life Technologies) and oligo(dT)15 primer (Promega). The mRNA transcript levels of selected IVSPER genes were measured by quantitative reverse transcription-PCR (qRT-PCR) using a LightCycler 480 System (Roche) and SYBR Green I Master Mix (Roche). Expression levels were normalized relative to a housekeeping wasp gene (elongation factor 1 ELF-1). Each sample was evaluated in triplicate, and the total reaction volume per well was 3 μl, including 0.5 μM of each primer and cDNA corresponding to 0.88 ng of total RNA. The amplification program consisted of an initial step at 95°C for 10 min, followed by 45 cycles of 95°C for 10 s, 58°C for 10 s, and 72°C for 10 s. The primers used for this analysis are listed in S4 Table.

### qPCR data analysis

Data were acquired using Light-Cycler 480 software. PCR amplification efficiency (E) for each primer pair was determined by linear regression of a dilution series (5x) of the cDNA pool. Relative expression, using the housekeeping gene ELF-1 as a reference, was calculated through advanced relative quantification (Efficiency method) software provided by Light-Cycler 480 software. For statistical analyses, Levene’s and Shapiro-Wilk tests were employed to verify homogeneity of variance and normal distribution of data among the tested groups. Differences in gene relative expression between developmental stages and between ds*GFP* and ds*U16*-injected females were assessed using a two-tailed unpaired t-test for group comparison. In cases where homogeneity of variance was not assumed, a Welch-test was used to compare gene relative expression between groups. A p-value < 0.05 was considered significant. All statistical analyses were conducted using R [63]. Detailed statistical analyses of qPCR results are provided in S2 Dataset.

## Supporting information

S1 Dataset *Hyposoter didymator* Hi-C genome assembly. The dataset includes: **Fig A.** Figure depicting the Hi-C scaffold contact map; **Table A.** Table presenting the Hi-C scaffolds containing HdIV loci; **Fig B.** Figure displaying the pairwise comparisons of HdIV segments located in close proximity within the *H*. *didymator* scaffolds.
(DOCX)

S2 Dataset Raw data and statistical analyses of qPCR data. The dataset includes raw data and statistical analyses for: Genomic DNA amplification of IVSPER genes at four different *H*. *didymator* pupal stages, Table A. Raw data and Table B. Statistical analyses; Genomic DNA amplification of IVSPER and HdIV segment genes in ds*GFP* and ds*U16*-injected wasps, Table C. Raw data and Table D. Statistical analyses; RNA quantification of IVSPER genes in ds*GFP* and ds*U16*-injected wasps, Table E. Raw data and Table F. Statistical analyses; DNA amplification of Hd29 segment in ds*GFP* and ds*U16*-injected wasps, Table G. Raw data and Table H. Statistical analyses.
(DOCX)

S3 Dataset Sequence analysis and alignment of the U16 gene from *H*. *didymator* to four other wasp species that harbor IVs. The dataset includes: **Table A.** Raw sequence of U16 proteins. **Table B.** Position of the PriCT-domain in U16 proteins. **Fig A.** Detail of the predicted secondary structure of the PricT-2 domain in the *H*. *didymator* U16 protein. **Fig B.** Subcellular localization of U16 predicted by DeepLoc 2.0.
(DOCX)

S1 Table Read depth of HdIV loci on each scaffold of the *H*. *didymator* genome. (DOCX)

S2 Table List of the peaks predicted in *H*. *didymator* genome scaffolds using MACS2 algorithm. (DOCX)

S3 Table Read depth of HdIV amplified regions in calyx cell DNA from ds*GFP*- and ds*U16*-injected female pupae. (DOCX)

S4 Table List of primers used in the present work. (DOCX)

S1 Fig DNA amplification patterns of HdIV loci in calyx cells of *H*. *didymator*. (DOCX)

S2 Fig HdIV amplified regions in Scaffold-11. (DOCX)

S3 Fig MEME analysis of boundaries of the predicted MACS2 HdIV amplified regions. (DOCX)
