# Supplementary material for: Identification of a viral gene essential for the genome replication of a domesticated endogenous virus in ichneumonid parasitoid wasps
Source: PLoS Pathog. 2024 Apr 25;20(4):e1011980. doi: 10.1371/journal.ppat.1011980 (PMC11075835; doi:10.1371/journal.ppat.1011980)

**S1 Dataset.  *Hyposoter didymator* Hi-C genome assembly.**

The dataset includes:

**Fig A.** Figure depicting the Hi-C scaffold contact map, illustrating the spatial interactions between genomic regions.

**Table A.** Table presenting the Hi-C scaffolds containing HdIV loci.

**Fig B.** Figure displaying the pairwise comparisons of HdIV segments located in close proximity within the *H. didymator* scaffolds, highlighting their genomic relationships.

**
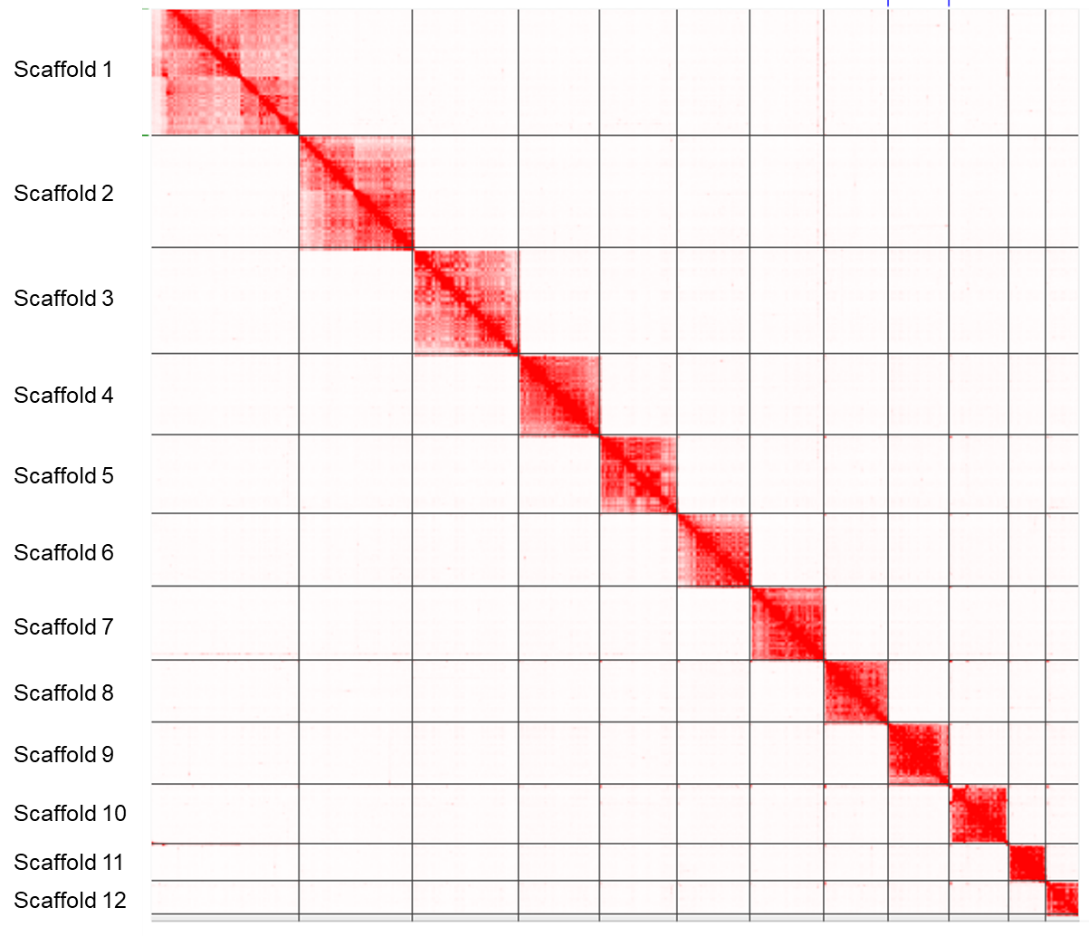
Fig A. Hi‐C scaffold contact map.** Chromatin contact intensities, quantified from Hi-C data, are represented in the matrix displaying various genomic regions. Blocks with a darker red color signify higher contact intensity. Frames are used to demarcate putative chromosomal units.

**Table A. List of Hi-C scaffolds containing HdIV loci.** This table presents the correspondence between the 12 largest scaffolds obtained through proximity ligation technology (Hi-C) and the scaffolds from the assembly in [1]. For each *H. didymator* IV (HdIV) locus, the start and end positions are provided. The scaffolds encompass 59 out of the 60 proviral loci described in Legeai et al., 2020. Notably, the 6 kbp-long scaffold containing segment Hd51 has not been merged with others through proximity ligation technology, likely due to a ~1.4 kbp region of low complexity ('NNNN').

| **New scaffold name** | **Scaffold size (nt)** | **HdIV locus** | **HdIV locus start** | **HdIVl locus end** | **HdIV locus size (nt)** | **Previous scaffold containing the HdIV locus (Legeai et al., 2020)** |
| --- | --- | --- | --- | --- | --- | --- |
| Scaffold-1 | 29,280,370 | Hd26 | 13523617 | 13528634 | 5,017 | scaffold175_len12456768 |
|  |  | Hd38 | 20666612 | 20670275 | 3,663 | scaffold175_len12456768 |
|  |  | Hd36 | 20670791 | 20674528 | 3,737 | scaffold175_len12456768 |
|  |  | Hd50 | 27247704 | 27253490 | 5,786 | scaffold357_len4004213 |
| Scaffold-2 | 22,662,106 | Hd49 | 2124324 | 2129588 | 5,264 | scaffold128246_len1305886 |
|  |  | U37 | 2735040 | 2736878 | 1,838 | scaffold29771_len25145 |
|  |  | Hd46 | 2737536 | 2741644 | 4,108 | scaffold29771_len25145 |
|  |  | Hd43 | 2744162 | 2748320 | 4,158 | scaffold1868_len202142 |
|  |  | Hd22 | 5839467 | 5843644 | 4,177 | scaffold119_len3623981 |
|  |  | Hd44.1 | 16284645 | 16287653 | 3,008 | scaffold128243_len5513913 |
|  |  | Hd44.2 | 16289605 | 16294435 | 4,830 | scaffold128243_len5513913 |
| Scaffold-3 | 20,844,088 | Hd30 | 419458 | 423621 | 4,163 | scaffold67_len6283432 |
|  |  | Hd48 | 16689745 | 16699417 | 9,672 | scaffold429_len1434187 |
| Scaffold-4 | 15,928,701 | Hd19 | 6231095 | 6235534 | 4,439 | scaffold198_len3327987 |
|  |  | Hd41 | 12119882 | 12127834 | 7,952 | scaffold184_len7628668 |
|  |  | Hd45.2 | 12329917 | 12331967 | 2,050 | scaffold184_len7628668 |
|  |  | Hd45.1 | 15910208 | 15914421 | 4,213 | scaffold28498_len4159 |
| Scaffold-5 | 15,286,740 | Hd13 | 5876265 | 5882021 | 5,756 | scaffold128241_len6033897 |
| Scaffold-6 | 14,543,462 | Hd40 | 2988772 | 2992265 | 3,493 | scaffold127348_len3747147 |
|  |  | Hd9 | 7229774 | 7247665 | 17,891 | scaffold128215_len912714 |
|  |  | Hd1 | 11708977 | 11723747 | 14,77 | scaffold90_len1092007 |
|  |  | Hd31-34 | 11868292 | 11872410 | 4,118 | scaffold127549_len2774194 |
| Scaffold-7 | 14,420,521 | Hd27 | 6697648 | 6701649 | 4,001 | scaffold127548_len15678958 |
|  |  | Hd5 | 7084945 | 7098657 | 13,712 | scaffold127548_len15678958 |
|  |  | Hd47 | 7900815 | 7905317 | 4,502 | scaffold127548_len15678958 |
|  |  | IVSPER-5 | 9178274 | 9179903 | 1,629 | scaffold127548_len15678958 |
|  |  | IVSPER-3 | 9252903 | 9278334 | 25,431 | scaffold127548_len15678958 |
|  |  | IVSPER-4 | 13191258 | 13207069 | 15,811 | scaffold127548_len15678958 |
|  |  | Hd7 | 13968918 | 13976983 | 8,065 | scaffold127548_len15678958 |
|  |  | Hd2 | 14085672 | 14099608 | 13,936 | scaffold127548_len15678958 |
|  |  | Hd6 | 14221056 | 14231516 | 10,460 | scaffold127548_len15678958 |
|  |  | Hd2-like | 14388343 | 14389879 | 1,536 | scaffold49647_len1586 |
| Scaffold-8 | 12,449,997 | Hd20 | 1942407 | 1949270 | 6,863 | scaffold144_len1844462 |
|  |  | Hd8 | 6823745 | 6831100 | 7,355 | scaffold377_len2592399 |
|  |  | Hd4 | 7097009 | 7107334 | 10,325 | scaffold377_len2592399 |
|  |  | Hd17 | 9012106 | 9019835 | 7,729 | scaffold351_len5773413 |
|  |  | Hd18 | 9364794 | 9369489 | 4,695 | scaffold351_len5773413 |
| Scaffold-9 | 12,140,457 | Hd39 | 5744955 | 5749076 | 4,121 | scaffold22_len5910489 |
| Scaffold-10 | 11,865,606 | Hd23.2 | 3553203 | 3556564 | 3,361 | scaffold128213_len363895 |
|  |  | Hd23.1 | 3592527 | 3596983 | 4,456 | scaffold128213_len363895 |
|  |  | Hd25 | 9399172 | 9403345 | 4,173 | scaffold161_len2602850 |
| Scaffold-11 | 7,176,887 | Hd10 | 453966 | 460472 | 6,506 | scaffold59_len2934223 |
|  |  | Hd11 | 768148 | 777337 | 9,189 | scaffold59_len2934223 |
|  |  | Hd16 | 2262941 | 2270644 | 7,703 | scaffold59_len2934223 |
|  |  | Hd12 | 2280031 | 2285932 | 5,901 | scaffold59_len2934223 |
|  |  | Hd29 | 3146273 | 3150628 | 4,355 | scaffold91_len761975 |
|  |  | IVSPER-2 | 3155228 | 3181838 | 26,610 | scaffold91_len761975 |
|  |  | Hd24 | 3182748 | 3187444 | 4,696 | scaffold91_len761975 |
|  |  | Hd33 | 3234245 | 3238079 | 3,834 | scaffold91_len761975 |
|  |  | Hd15 | 3249051 | 3254037 | 4,986 | scaffold91_len761975 |
|  |  | IVSPER-1 | 3255266 | 3269285 | 14,019 | scaffold91_len761975 |
|  |  | Hd14 | 3759577 | 3764772 | 5,195 | scaffold64_len2416597 |
|  |  | Hd32 | 3811943 | 3819858 | 7,915 | scaffold64_len2416597 |
|  |  | Hd42 | 6043764 | 6046920 | 3,156 | scaffold64_len2416597 |
|  |  | Hd21 | 6076348 | 6080715 | 4,367 | scaffold64_len2416597 |
|  |  | Hd3 | 6685111 | 6695124 | 10,013 | scaffold65_len1016704 |
|  |  | Hd37 | 6786206 | 6789913 | 3,707 | scaffold65_len1016704 |
| Scaffold-12 | 6,664,771 | Hd28 | 308843 | 313456 | 4,613 | scaffold264_len1259582 |
|  |  | Hd35 | 2941876 | 2945585 | 3,709 | scaffold116_len1992701 |
|  |  | Hd51 | 1077 | 5708 | 4,631 | scaffold82201_len6109 |

Reference:

1. Legeai F, Santos BF, Robin S, Bretaudeau A, Dikow RB, Lemaitre C, et al. Genomic architecture of endogenous ichnoviruses reveals distinct evolutionary pathways leading to virus domestication in parasitic wasps. BMC Biol. 2020 Jul 24;18(1):89. doi: 10.1186/s12915-020-00822-3.

**Fig B. Pairwise comparison of HdIV segments located at close vicinity within *H. didymator* scaffolds.** Dot plots were generated using NCBI Blastn searches 'align two or more sequences'. (a) Dot plot illustrating the comparison between Hd36 (Query_213343; 3,738 nt) and Hd38 (Query_213345; 3,664 nt) located on Scaffold-1. (b) Dot plot depicting the comparison between Hd46 (Query_384927; 4,109 nt) and Hd43 (Query_384929; 4,159 nt) located on Scaffold-2. (c) Dot plot showing the comparison between Hd44.1 (Query_40789; 3,009 nt) and Hd44.2 (Query_40791; 4,831 nt) located on Scaffold-2. (d) Dot plot illustrating the comparison between Hd12 (Query_11753; 5,902 nt) and Hd16 (Query_11755; 7,704 nt) located on Scaffold-11.


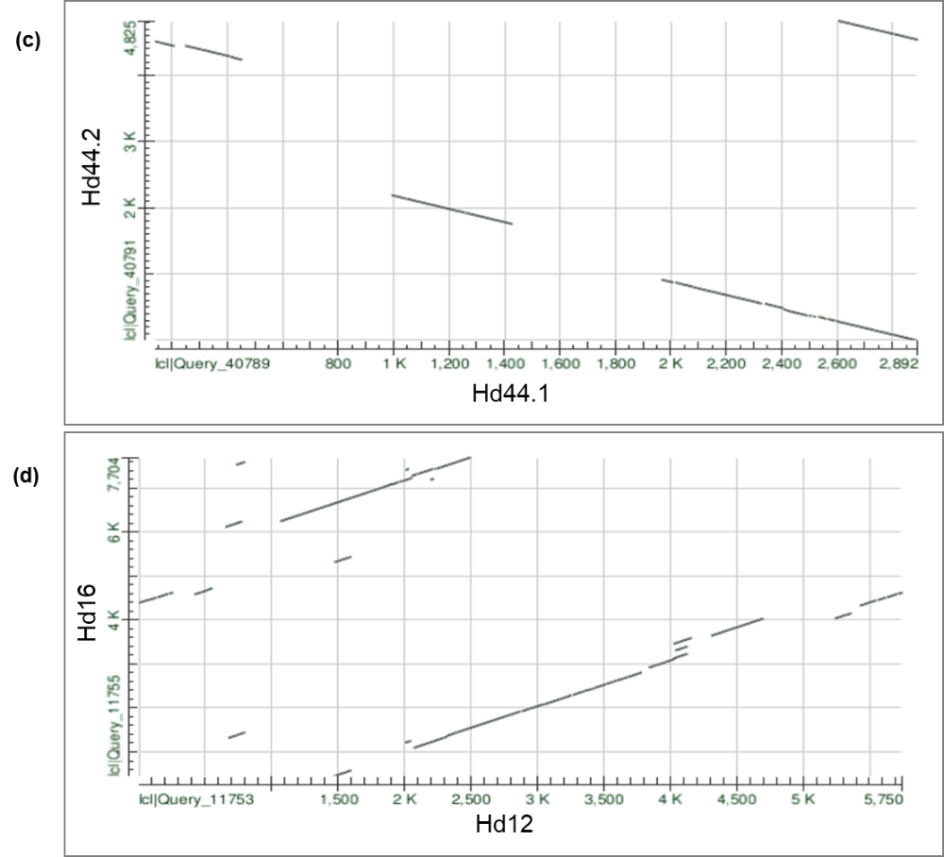

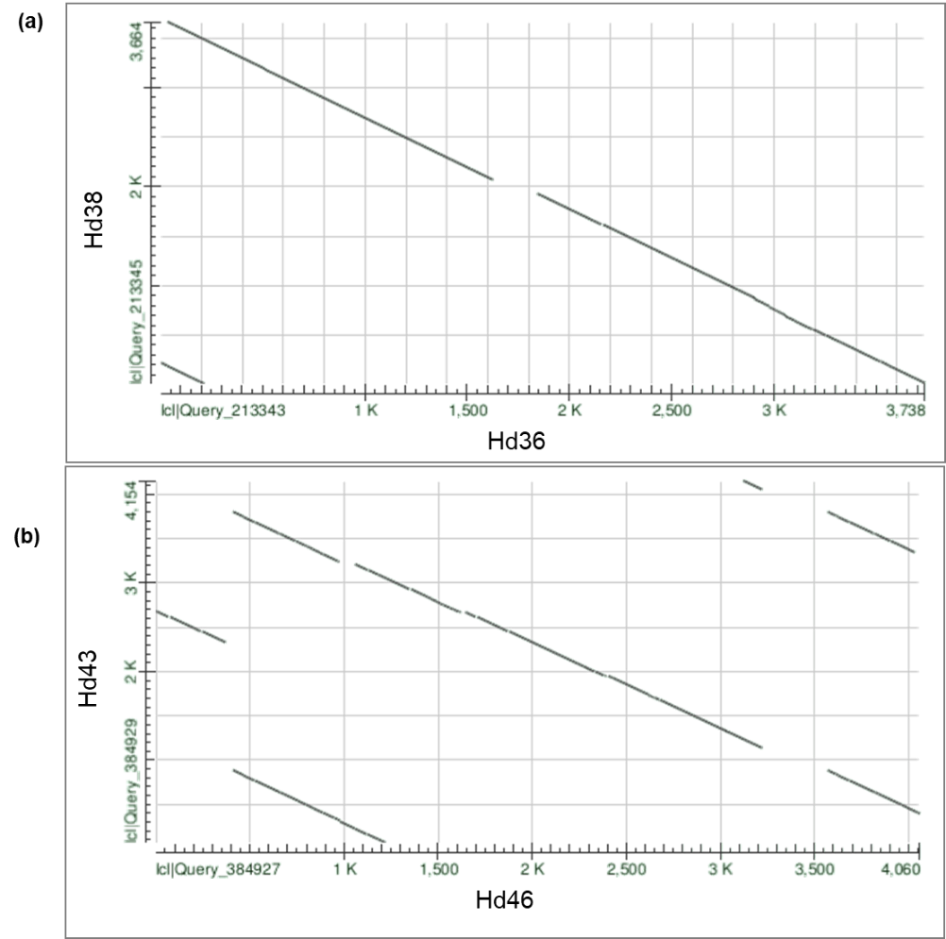

Supplement: S1 Dataset — The dataset includes: Fig A. Figure depicting the Hi-C scaffold contact map; Table A. Table presenting the Hi-C scaffolds containing HdIV loci; Fig B. Figure displaying the pairwise comparisons of HdIV segments located in close proximity within the H. didymator scaffolds. (DOCX) [file ppat.1011980.s001.docx]
