# Supplementary material for: Identification of a viral gene essential for the genome replication of a domesticated endogenous virus in ichneumonid parasitoid wasps
Source: PLoS Pathog. 2024 Apr 25;20(4):e1011980. doi: 10.1371/journal.ppat.1011980 (PMC11075835; doi:10.1371/journal.ppat.1011980)
Supplement: S2 Dataset — The dataset includes raw data and statistical analyses for: Genomic DNA amplification of IVSPER genes at four different H. didymator pupal stages, Table A. Raw data and Table B. Statistical analyses; Genomic DNA amplification of IVSPER and HdIV segment genes in dsGFP and dsU16-injected wasps, Table C. Raw data and Table D. Statistical analyses; RNA quantification of IVSPER genes in dsGFP and dsU16-injected wasps, Table E. Raw data and Table F. Statistical analyses; DNA amplification of Hd29 segment in dsGFP and dsU16-injected wasps, Table G. Raw data and Table H. Statistical analyses. (DOCX) [file ppat.1011980.s002.docx]

**S2 Dataset. Raw data and statistical analyses of qPCR analyses.**

The dataset includes:

Genomic DNA amplification of IVSPER genes at four different *H. didymator* pupal stages:

**Table A.** Raw data depicting the genomic DNA amplification patterns of IVSPER genes at four different pupal stages.

**Table B.** Statistical analyses of the genomic DNA amplification data for IVSPER genes.

Genomic DNA amplification of IVSPER and HdIV segment genes in ds*GFP* and ds*U16*-injected wasps:

**Table C.** Raw data illustrating the genomic DNA amplification of IVSPER and HdIV segment genes in ds*GFP*- and ds*U16*-injected wasps.

**Table D.** Statistical analyses of the genomic DNA amplification data for IVSPER and HdIV segment genes.

RNA quantification of IVSPER genes in ds*GFP* and ds*U16*-injected wasps:

**Table E.** Raw data presenting RNA quantification data for IVSPER genes in dsG*FP*- and ds*U16*-injected wasps.

**Table F.** Statistical analyses of the RNA quantification data for IVSPER genes.

DNA amplification of Hd29 segment in ds*GFP* and ds*U16*-injected wasps:

**Table G.** Raw data illustrating the DNA amplification patterns of different forms of the Hd29 segment in ds*GFP*- and ds*U16*-injected wasps.

**Table H.** Statistical analyses of the DNA amplification data for different forms of the Hd29 segment.

**Genomic DNA amplification of IVSPER genes at four different *H. didymator* pupal stages**

**Table A.** Genomic DNA amplification (Cf Fig 2A). Raw data.

**Table B.** Genomic DNA amplification (Cf Fig 2A). Statistical analysis.

| **evREPs genes relative amplification comparison between calyx pupal stages** | | **Levene's Test for equality of variance** | | **t-test Equality of Means** | | | | **95% confidence interval of the difference** | |
| --- | --- | --- | --- | --- | --- | --- | --- | --- | --- |
| **Genes** | **Condition** | F | P value | t | df | P value | Significance | Lower | upper |
| **IVSP2-2** | **Stage 1 vs Stage 2** | 2,092 | 0,22 | -8,254 | 4 | 0,00118 | ** | -22,912 | -11,378 |
|  | **Stage 2 vs Stage 3** | 2,152 | 0,21 | -2,039 | 4 | 0,111 | n.s | -46,272 | 7,08 |
|  | **Stage 3 vs Stage 4** | 0,591 | 0,48 | -1,069 | 4 | 0,345 | n.s | -40,931 | 18,122 |
|  | **Stage 2 vs Stage 4** | 0,351 | 0,58 | 5,7 | 4 | 0,00468 | ** | -46,07 | -15,89 |
| **IVSP3-1** | **Stage 1 vs Stage 2** | 1,069 | 0,35 | -3,85 | 4 | < 0,001 | *** | -28,75 | -24,617 |
|  | **Stage 2 vs Stage 3** | 2,806 | 0,16 | -5,06 | 4 | 0,0071 | ** | -82,044 | -23,91 |
|  | **Stage 3 vs Stage 4** | 2,03 | 0,2 | -2,99 | 4 | 0,0401 | * | -60,928 | -2,306 |
|  | **Stage 2 vs Stage 4** | 0,858 | 0,4 | -49,44 | 4 | < 0,001 | *** | -89,343 | -79,843 |
| **IVp12-1** | **Stage 1 vs Stage 2** | 1,045 | 0,36 | -20,33 | 4 | < 0,001 | *** | -30,64 | -23,276 |
|  | **Stage 2 vs Stage 3** | 1,434 | 0,29 | -4,57 | 4 | 0,0102 | * | -97,491 | -23,081 |
|  | **Stage 3 vs Stage 4** | 0,401 | 0,56 | -2,832 | 4 | 0,0472 | * | -79,32 | -0,786 |
|  | **Stage 2 vs Stage 4** | 1,539 | 0,28 | -16 | 4 | < 0,001 | *** | -115,99 | -81,69 |
| **IVp53-1** | **Stage 1 vs Stage 2** | 1,238 | 0,32 | -10,25 | 4 | < 0,001 | *** | -33,086 | -18,981 |
|  | **Stage 2 vs Stage 3** | 1,8 | 0,25 | -6,903 | 4 | 0,0023 | ** | -11,275 | -47,443 |
|  | **Stage 3 vs Stage 4** | 0,183 | 0,69 | -2,051 | 4 | 0,066 | n.s | -130,27 | 6,633 |
|  | **Stage 2 vs Stage 4** | 0,856 | 0,4 | -6,386 | 4 | 0,003 | ** | -202,55 | -79,806 |
| **IVp53-2** | **Stage 1 vs Stage 2** | 2,282 | 0,2 | -6,554 | 4 | 0,0028 | ** | -9,156 | -3,707 |
|  | **Stage 2 vs Stage 3** | 0,281 | 0,62 | -3,848 | 4 | 0,018 | * | -11,867 | -1,919 |
|  | **Stage 3 vs Stage 4** | 0,214 | 0,66 | -2,667 | 4 | 0,055 | n.s | -14,072 | 0,281 |
|  | **Stage 2 vs Stage 4** | 0,903 | 0,39 | -5,94 | 4 | 0,004 | ** | -20,233 | -7,343 |
| **U1** | **Stage 1 vs Stage 2** | 3,051 | 0,15 | -9,681 | 4 | < 0,001 | *** | -6,67 | -3,697 |
|  | **Stage 2 vs Stage 3** | 0,939 | 0,38 | -2,267 | 4 | 0,085 | n.s | -11,087 | 1,119 |
|  | **Stage 3 vs Stage 4** | 0,803 | 0,42 | -2,307 | 4 | 0,082 | n.s | -11,338 | 1,0465 |
|  | **Stage 2 vs Stage 4** | 0,007 | 0,93 | -12 | 4 | < 0,001 | *** | -12,474 | -7,785 |
| **U6** | **Stage 1 vs Stage 2** | 2,617 | 0,18 | -7,188 | 4 | 0,0019 | ** | -29,859 | -13,219 |
|  | **Stage 2 vs Stage 3** | 1,06 | 0,36 | -2,646 | 4 | 0,057 | n.s | -65,567 | 1,571 |
|  | **Stage 3 vs Stage 4** | 1,014 | 0,37 | -1,518 | 4 | 0,2 | n.s | -52,173 | 15,287 |
|  | **Stage 2 vs Stage 4** | 3E-04 | 0,98 | -11,48 | 4 | < 0,001 | *** | -62,645 | -38,236 |
| **U11** | **Stage 1 vs Stage 2** | 3,77 | 0,14 | -8,954 | 4 | < 0,001 | *** | -37,674 | -19,84 |
|  | **Stage 2 vs Stage 3** | 1,261 | 0,32 | -2,793 | 4 | 0,049 | * | -95,868 | -0,301 |
|  | **Stage 3 vs Stage 4** | 1,067 | 0,35 | -1,757 | 4 | 0,15 | n.s | 79,037 | 17,756 |
|  | **Stage 2 vs Stage 4** | 0,041 | 0,84 | -14,81 | 4 | < 0,001 | *** | -93,485 | -63,965 |
| **U15** | **Stage 1 vs Stage 2** | 1,452 | 0,29 | -8,439 | 4 | 0,00108 | ** | -21,277 | -10,743 |
|  | **Stage 2 vs Stage 3** | 1,022 | 0,36 | -3,125 | 4 | 0,035 | * | -68,734 | -4,059 |
|  | **Stage 3 vs Stage 4** | 1,158 | 0,34 | -1,984 | 4 | 0,11 | n.s | -55,084 | 9,166 |
|  | **Stage 2 vs Stage 4** | 0,084 | 0,78 | -25,48 | 4 | < 0,001 | *** | -65,823 | -52,89 |
| **U22** | **Stage 1 vs Stage 2** | 0,968 | 0,38 | -19,3 | 4 | < 0,001 | *** | -60,964 | -45,629 |
|  | **Stage 2 vs Stage 3** | 1,37 | 0,3 | -4,899 | 4 | 0,00804 | ** | -187,32 | -51,814 |
|  | **Stage 3 vs Stage 4** | 0,618 | 0,47 | -3,922 | 4 | 0,017 | * | -172,68 | -29,541 |
|  | **Stage 2 vs Stage 4** | 1,63 | 0,27 | -24,05 | 4 | < 0,001 | *** | -246,15 | -195,21 |
| **U23** | **Stage 1 vs Stage 2** | 1,134 | 0,34 | -9,395 | 4 | < 0,001 | *** | -78,014 | -42,423 |
|  | **Stage 2 vs Stage 3** | 0,538 | 0,5 | -5,116 | 4 | 0,0069 | ** | -212,35 | -62,953 |
|  | **Stage 3 vs Stage 4** | 0,263 | 0,63 | -3,956 | 4 | 0,016 | * | -190,73 | -33,424 |
|  | **Stage 2 vs Stage 4** | 0,34 | 0,59 | -19,69 | 4 | < 0,001 | *** | -284,95 | -214,51 |
| **XRCC1** | **Stage 1 vs Stage 2** | 1,259 | 0,32 | -7,221 | 4 | 0,0019 | ** | -0,67 | -0,297 |
|  | **Stage 2 vs Stage 3** | 0,111 | 0,75 | -1,417 | 4 | 0,22 | n.s | -0,336 | 0,109 |
|  | **Stage 3 vs Stage 4** | 0,974 | 0,37 | -2,861 | 4 | 0,045 | * | -1,039 | -0,015 |
|  | **Stage 2 vs Stage 4** | 0,665 | 0,46 | -3,37 | 4 | 0,028 | * | -1,169 | -0,113 |

| **evREPs genes relative amplification comparison between calyx stage 1 & hind legs stage 1** | | **Levene's Test for equality of variance** | | **t-test Equality of Means** | | | | **95% confidence interval of the difference** | |
| --- | --- | --- | --- | --- | --- | --- | --- | --- | --- |
| **Genes** | **Condition** | F | P value | t | df | P value | Significance | Lower | upper |
| **IVSP2-2** | **Calyx vs Hind legs stage 1** | 0,623 | 0,47 | -1,38 | 4 | 0,24 | n.s | -0,497 | 0,167 |
| **IVSP3-1** | **Calyx vs Hind legs stage 1** | 0,297 | 0,61 | -0,9 | 4 | 0,41 | n.s | -0,633 | 0,323 |
| **IVp12-1** | **Calyx vs Hind legs stage 1** | 0,018 | 0,9 | -0,67 | 4 | 0,54 | n.s | -0,226 | 0,138 |
| **IVp53-1** | **Calyx vs Hind legs stage 1** | 0,264 | 0,63 | -2,71 | 4 | 0,053 | n.s | -0,134 | 0,002 |
| **IVp53-2** | **Calyx vs Hind legs stage 1** | 0,188 | 0,68 | -1,24 | 4 | 0,28 | n.s | -0,584 | 0,222 |
| **U1** | **Calyx vs Hind legs stage 1** | 0,102 | 0,76 | -0,4 | 4 | 0,7 | n.s | -0,181 | 0,135 |
| **U6** | **Calyx vs Hind legs stage 1** | 4E-04 | 0,98 | -0,34 | 4 | 0,75 | n.s | -0,556 | 0,435 |
| **U11** | **Calyx vs Hind legs stage 1** | 0,067 | 0,8 | -0,74 | 4 | 0,5 | n.s | -0,499 | 0,29 |
| **U15** | **Calyx vs Hind legs stage 1** | 0,075 | 0,79 | 0,785 | 4 | 0,47 | n.s | -0,086 | 0,154 |
| **U22** | **Calyx vs Hind legs stage 1** | 0,253 | 0,64 | -1,27 | 4 | 0,27 | n.s | -0,759 | 0,281 |
| **U23** | **Calyx vs Hind legs stage 1** | 0,013 | 0,91 | -1,25 | 4 | 0,27 | n.s | -0,45 | 0,17 |
| **XRCC1** | **Calyx vs Hind legs stage 1** | 0,099 | 0,76 | 0,418 | 4 | 0,69 | n.s | -0,035 | 0,048 |

**Genomic DNA amplification of IVSPER and HdIV segment genes in ds*GFP* and ds*U16*-injected wasps**

**Table C.** Genomic DNA amplification (Cf Fig 8). Raw Data

| Biological replica ID | Sample | Target gene | Efficiency | Mean Ct Target gene | Mean Ct reference (*ELF-1*) | Relative amplification to *ELF-1* |
| --- | --- | --- | --- | --- | --- | --- |
| C1 | ds-GFP-calyx-R1 |  |  | 19,59553 | 19,0152365 | 1,361949 |
| C2 | ds-GFP-calyx-R2 |  |  | 20,6574414 | 20,1914827 | 1,529959 |
| C3 | ds-GFP-calyx-R3 |  |  | 22,6384005 | 22,0709032 | 1,532932 |
| C4 | ds-GFP-calyx-R4 |  |  | 20,5697389 | 20,0149517 | 1,435346 |
| C1 | ds-GFP-hind legs-R1 |  |  | 18,6256389 | 18,0663004 | 1,334186 |
| C2 | ds-GFP-hind legs-R2 |  |  | 20,48692 | 20,0652367 | 1,567289 |
| C3 | ds-GFP-hind legs-R3 |  |  | 22,845923 | 22,3636463 | 1,637000 |
| C4 | ds-GFP-hind legs-R4 | *H1* | 1,91 | 20,943969 | 20,3911466 | 1,456764 |
| T1 | ds-U16-calyx-R1 |  |  | 19,518832 | 18,9284962 | 1,348910 |
| T2 | ds-U16-calyx-R2 |  |  | 19,8610046 | 19,3049705 | 1,398002 |
| T3 | ds-U16-calyx-R3 |  |  | 20,9375654 | 20,4723268 | 1,546223 |
| T4 | ds-U16-calyx-R4 |  |  | 20,0800388 | 19,6629592 | 1,549368 |
| T1 | ds-U16-hind legs-R1 |  |  | 19,9706482 | 19,5191085 | 1,507376 |
| T2 | ds-U16-hind legs-R2 |  |  | 19,9674699 | 19,6603043 | 1,663423 |
| T3 | ds-U16-hind legs-R3 |  |  | 20,2764746 | 19,7922 | 1,490359 |
| T4 | ds-U16-hind legs-R4 |  |  | 19,241577 | 18,6709878 | 1,353652 |
| C1 | ds-GFP-calyx-R1 |  |  | 19,4677579 | 19,0152365 | 0,988178 |
| C2 | ds-GFP-calyx-R2 |  |  | 20,7507569 | 20,1914827 | 0,936855 |
| C3 | ds-GFP-calyx-R3 |  |  | 22,5875102 | 22,0709032 | 0,991994 |
| C4 | ds-GFP-calyx-R4 |  |  | 20,7669487 | 20,0149517 | 0,821496 |
| C1 | ds-GFP-hind legs-R1 |  |  | 18,4757965 | 18,0663004 | 1,002356 |
| C2 | ds-GFP-hind legs-R2 |  |  | 20,5587034 | 20,0652367 | 0,977061 |
| C3 | ds-GFP-hind legs-R3 |  |  | 22,6587215 | 22,3636463 | 1,155319 |
| C4 | ds-GFP-hind legs-R4 | *rpl* | 1,95 | 21,0800916 | 20,3911466 | 0,861762 |
| T1 | ds-U16-calyx-R1 |  |  | 19,5036461 | 18,9284962 | 0,909271 |
| T2 | ds-U16-calyx-R2 |  |  | 20,0139437 | 19,3049705 | 0,836328 |
| T3 | ds-U16-calyx-R3 |  |  | 20,178152 | 20,4723268 | 1,663659 |
| T4 | ds-U16-calyx-R4 |  |  | 20,3424499 | 19,6629592 | 0,857632 |
| T1 | ds-U16-hind legs-R1 |  |  | 19,7924038 | 19,5191085 | 1,122426 |
| T2 | ds-U16-hind legs-R2 |  |  | 20,1167106 | 19,6603043 | 0,995372 |
| T3 | ds-U16-hind legs-R3 |  |  | 20,8689641 | 19,7922 | 0,659075 |
| T4 | ds-U16-hind legs-R4 |  |  | 19,467345 | 18,6709878 | 0,781319 |
| C1 | ds-GFP-calyx-R1 |  |  | 13,1323101 | 19,0152365 | 77,827239 |
| C2 | ds-GFP-calyx-R2 |  |  | 14,5832067 | 20,1914827 | 66,952807 |
| C3 | ds-GFP-calyx-R3 |  |  | 16,6745483 | 22,0709032 | 61,112428 |
| C4 | ds-GFP-calyx-R4 |  |  | 14,2117758 | 20,0149517 | 75,764018 |
| C1 | ds-GFP-hind legs-R1 |  |  | 18,4850224 | 18,0663004 | 1,205339 |
| C2 | ds-GFP-hind legs-R2 |  |  | 20,5951811 | 20,0652367 | 1,179106 |
| C3 | ds-GFP-hind legs-R3 |  |  | 22,713817 | 22,3636463 | 1,407404 |
| C4 | ds-GFP-hind legs-R4 | *IVp53-1* | 1,93 | 20,7782882 | 20,3911466 | 1,306025 |
| T1 | ds-U16-calyx-R1 |  |  | 18,1341403 | 18,9284962 | 2,735833 |
| T2 | ds-U16-calyx-R2 |  |  | 17,1136268 | 19,3049705 | 6,921361 |
| T3 | ds-U16-calyx-R3 |  |  | 20,8724719 | 20,4723268 | 1,297597 |
| T4 | ds-U16-calyx-R4 |  |  | 19,9205818 | 19,6629592 | 1,395879 |
| T1 | ds-U16-hind legs-R1 |  |  | 19,7416221 | 19,5191085 | 1,423232 |
| T2 | ds-U16-hind legs-R2 |  |  | 20,0013064 | 19,6603043 | 1,321322 |
| T3 | ds-U16-hind legs-R3 |  |  | 20,105068 | 19,7922 | 1,350541 |
| T4 | ds-U16-hind legs-R4 |  |  | 18,9645733 | 18,6709878 | 1,329106 |
| C1 | ds-GFP-calyx-R1 |  |  | 16,1858129 | 19,0152365 | 19,005210 |
| C2 | ds-GFP-calyx-R2 |  |  | 18,7174307 | 20,1914827 | 8,821048 |
| C3 | ds-GFP-calyx-R3 |  |  | 20,5314899 | 22,0709032 | 10,331572 |
| C4 | ds-GFP-calyx-R4 |  |  | 17,5671647 | 20,0149517 | 15,964715 |
| C1 | ds-GFP-hind legs-R1 |  |  | 20,7798423 | 18,0663004 | 0,574389 |
| C2 | ds-GFP-hind legs-R2 |  |  | 24,6203004 | 20,0652367 | 0,207571 |
| C3 | ds-GFP-hind legs-R3 |  |  | 25,8972613 | 22,3636463 | 0,451719 |
| C4 | ds-GFP-hind legs-R4 | *IVp53-2* | 1,86 | 23,3364667 | 20,3911466 | 0,575259 |
| T1 | ds-U16-calyx-R1 |  |  | 20,7002904 | 18,9284962 | 1,087501 |
| T2 | ds-U16-calyx-R2 |  |  | 21,271541 | 19,3049705 | 0,986636 |
| T3 | ds-U16-calyx-R3 |  |  | 23,5969088 | 20,4723268 | 0,517314 |
| T4 | ds-U16-calyx-R4 |  |  | 22,5795067 | 19,6629592 | 0,559559 |
| T1 | ds-U16-hind legs-R1 |  |  | 22,1166963 | 19,5191085 | 0,675934 |
| T2 | ds-U16-hind legs-R2 |  |  | 24,1192557 | 19,6603043 | 0,214821 |
| T3 | ds-U16-hind legs-R3 |  |  | 22,8766817 | 19,7922 | 0,508271 |
| T4 | ds-U16-hind legs-R4 |  |  | 21,5354983 | 18,6709878 | 0,543171 |
| C1 | ds-GFP-calyx-R1 |  |  | 13,7216112 | 19,0152365 | 45,858157 |
| C2 | ds-GFP-calyx-R2 |  |  | 14,7335681 | 20,1914827 | 52,103316 |
| C3 | ds-GFP-calyx-R3 |  |  | 17,0739178 | 22,0709032 | 39,413188 |
| C4 | ds-GFP-calyx-R4 |  |  | 14,716344 | 20,0149517 | 46,718656 |
| C1 | ds-GFP-hind legs-R1 |  |  | 19,1446861 | 18,0663004 | 0,641240 |
| C2 | ds-GFP-hind legs-R2 |  |  | 20,9027692 | 20,0652367 | 0,776481 |
| C3 | ds-GFP-hind legs-R3 |  |  | 23,3062591 | 22,3636463 | 0,749710 |
| C4 | ds-GFP-hind legs-R4 | *IVp12-1* | 1,95 | 21,3387618 | 20,3911466 | 0,725043 |
| T1 | ds-U16-calyx-R1 |  |  | 18,9208785 | 18,9284962 | 1,341888 |
| T2 | ds-U16-calyx-R2 |  |  | 17,4135781 | 19,3049705 | 4,748675 |
| T3 | ds-U16-calyx-R3 |  |  | 21,5106985 | 20,4723268 | 0,683250 |
| T4 | ds-U16-calyx-R4 |  |  | 20,5997268 | 19,6629592 | 0,722240 |
| T1 | ds-U16-hind legs-R1 |  |  | 20,6018452 | 19,5191085 | 0,653720 |
| T2 | ds-U16-hind legs-R2 |  |  | 20,5123909 | 19,6603043 | 0,764231 |
| T3 | ds-U16-hind legs-R3 |  |  | 20,8141754 | 19,7922 | 0,683637 |
| T4 | ds-U16-hind legs-R4 |  |  | 19,7612538 | 18,6709878 | 0,642073 |
| C1 | ds-GFP-calyx-R1 |  |  | 14,0437004 | 19,0152365 | 49,477796 |
| C2 | ds-GFP-calyx-R2 |  |  | 15,2206099 | 20,1914827 | 51,595379 |
| C3 | ds-GFP-calyx-R3 |  |  | 17,6522161 | 22,0709032 | 38,619527 |
| C4 | ds-GFP-calyx-R4 |  |  | 15,1308893 | 20,0149517 | 48,467955 |
| C1 | ds-GFP-hind legs-R1 |  |  | 18,4897681 | 18,0663004 | 1,456802 |
| C2 | ds-GFP-hind legs-R2 |  |  | 20,2826508 | 20,0652367 | 1,788775 |
| C3 | ds-GFP-hind legs-R3 |  |  | 22,6306687 | 22,3636463 | 1,881665 |
| C4 | ds-GFP-hind legs-R4 | *IVSP3-1* | 1,91 | 20,6917841 | 20,3911466 | 1,714990 |
| T1 | ds-U16-calyx-R1 |  |  | 18,5923591 | 18,9284962 | 2,456703 |
| T2 | ds-U16-calyx-R2 |  |  | 17,5306993 | 19,3049705 | 6,315406 |
| T3 | ds-U16-calyx-R3 |  |  | 20,8359545 | 20,4723268 | 1,651309 |
| T4 | ds-U16-calyx-R4 |  |  | 19,8698938 | 19,6629592 | 1,775057 |
| T1 | ds-U16-hind legs-R1 |  |  | 19,841903 | 19,5191085 | 1,638337 |
| T2 | ds-U16-hind legs-R2 |  |  | 19,8763959 | 19,6603043 | 1,764402 |
| T3 | ds-U16-hind legs-R3 |  |  | 20,0557576 | 19,7922 | 1,719173 |
| T4 | ds-U16-hind legs-R4 |  |  | 18,9025627 | 18,6709878 | 1,685704 |
| C1 | ds-GFP-calyx-R1 |  |  | 13,5801427 | 19,0152365 | 54,046827 |
| C2 | ds-GFP-calyx-R2 |  |  | 14,4631637 | 20,1914827 | 67,233623 |
| C3 | ds-GFP-calyx-R3 |  |  | 17,0628396 | 22,0709032 | 43,346490 |
| C4 | ds-GFP-calyx-R4 |  |  | 14,7381265 | 20,0149517 | 49,668511 |
| C1 | ds-GFP-hind legs-R1 |  |  | 18,0311921 | 18,0663004 | 1,479906 |
| C2 | ds-GFP-hind legs-R2 |  |  | 19,6270554 | 20,0652367 | 2,013524 |
| C3 | ds-GFP-hind legs-R3 |  |  | 22,22342 | 22,3636463 | 1,732115 |
| C4 | ds-GFP-hind legs-R4 | *U11* | 1,94 | 20,3183247 | 20,3911466 | 1,591089 |
| T1 | ds-U16-calyx-R1 |  |  | 17,9864059 | 18,9284962 | 2,747307 |
| T2 | ds-U16-calyx-R2 |  |  | 16,6870484 | 19,3049705 | 8,405264 |
| T3 | ds-U16-calyx-R3 |  |  | 20,4574619 | 20,4723268 | 1,533677 |
| T4 | ds-U16-calyx-R4 |  |  | 19,6423381 | 19,6629592 | 1,514316 |
| T1 | ds-U16-hind legs-R1 |  |  | 19,4463287 | 19,5191085 | 1,562979 |
| T2 | ds-U16-hind legs-R2 |  |  | 19,1476786 | 19,6603043 | 2,097939 |
| T3 | ds-U16-hind legs-R3 |  |  | 19,7177705 | 19,7922 | 1,573433 |
| T4 | ds-U16-hind legs-R4 |  |  | 18,64003 | 18,6709878 | 1,494167 |
| C1 | ds-GFP-calyx-R1 |  |  | 13,0360643 | 19,0152365 | 72,485132 |
| C2 | ds-GFP-calyx-R2 |  |  | 14,0227365 | 20,1914827 | 83,758929 |
| C3 | ds-GFP-calyx-R3 |  |  | 16,5776634 | 22,0709032 | 54,900023 |
| C4 | ds-GFP-calyx-R4 |  |  | 14,1097327 | 20,0149517 | 70,053303 |
| C1 | ds-GFP-hind legs-R1 |  |  | 18,273571 | 18,0663004 | 1,147293 |
| C2 | ds-GFP-hind legs-R2 |  |  | 20,013615 | 20,0652367 | 1,406100 |
| C3 | ds-GFP-hind legs-R3 |  |  | 22,5875107 | 22,3636463 | 1,211589 |
| C4 | ds-GFP-hind legs-R4 | *U16* | 1,95 | 20,5842342 | 20,3911466 | 1,200060 |
| T1 | ds-U16-calyx-R1 |  |  | 18,1110116 | 18,9284962 | 2,304654 |
| T2 | ds-U16-calyx-R2 |  |  | 16,6493822 | 19,3049705 | 7,910714 |
| T3 | ds-U16-calyx-R3 |  |  | 20,7109433 | 20,4723268 | 1,165565 |
| T4 | ds-U16-calyx-R4 |  |  | 19,805709 | 19,6629592 | 1,227367 |
| T1 | ds-U16-hind legs-R1 |  |  | 19,7397078 | 19,5191085 | 1,162630 |
| T2 | ds-U16-hind legs-R2 |  |  | 19,6305163 | 19,6603043 | 1,377205 |
| T3 | ds-U16-hind legs-R3 |  |  | 19,9195701 | 19,7922 | 1,242487 |
| T4 | ds-U16-hind legs-R4 |  |  | 18,845361 | 18,6709878 | 1,183655 |
| C1 | ds-GFP-calyx-R1 |  |  | 12,8697363 | 19,0152365 | 98,888696 |
| C2 | ds-GFP-calyx-R2 |  |  | 14,0254053 | 20,1914827 | 103,919126 |
| C3 | ds-GFP-calyx-R3 |  |  | 16,395808 | 22,0709032 | 79,931165 |
| C4 | ds-GFP-calyx-R4 |  |  | 13,9689486 | 20,0149517 | 95,569462 |
| C1 | ds-GFP-hind legs-R1 |  |  | 18,1450422 | 18,0663004 | 1,656264 |
| C2 | ds-GFP-hind legs-R2 |  |  | 20,1068813 | 20,0652367 | 1,804484 |
| C3 | ds-GFP-hind legs-R3 |  |  | 22,4634145 | 22,3636463 | 1,864672 |
| C4 | ds-GFP-hind legs-R4 | *U22* | 1,92 | 20,4420605 | 20,3911466 | 1,811684 |
| T1 | ds-U16-calyx-R1 |  |  | 17,9628975 | 18,9284962 | 3,361360 |
| T2 | ds-U16-calyx-R2 |  |  | 16,6683145 | 19,3049705 | 10,114834 |
| T3 | ds-U16-calyx-R3 |  |  | 20,5715917 | 20,4723268 | 1,759825 |
| T4 | ds-U16-calyx-R4 |  |  | 19,671322 | 19,6629592 | 1,821402 |
| T1 | ds-U16-hind legs-R1 |  |  | 19,5984788 | 19,5191085 | 1,731278 |
| T2 | ds-U16-hind legs-R2 |  |  | 19,7000512 | 19,6603043 | 1,784347 |
| T3 | ds-U16-hind legs-R3 |  |  | 19,8398965 | 19,7922 | 1,782337 |
| T4 | ds-U16-hind legs-R4 |  |  | 18,6801582 | 18,6709878 | 1,765714 |
| C1 | ds-GFP-calyx-R1 |  |  | 13,0437471 | 19,0152365 | 133,567276 |
| C2 | ds-GFP-calyx-R2 |  |  | 14,4602125 | 20,1914827 | 123,849881 |
| C3 | ds-GFP-calyx-R3 |  |  | 16,8277938 | 22,0709032 | 102,886984 |
| C4 | ds-GFP-calyx-R4 |  |  | 14,5396286 | 20,0149517 | 104,501215 |
| C1 | ds-GFP-hind legs-R1 |  |  | 18,647734 | 18,0663004 | 2,156933 |
| C2 | ds-GFP-hind legs-R2 |  |  | 20,8656314 | 20,0652367 | 2,133534 |
| C3 | ds-GFP-hind legs-R3 |  |  | 23,0984921 | 22,3636463 | 2,565504 |
| C4 | ds-GFP-hind legs-R4 | *U23* | 1,86 | 21,3163259 | 20,3911466 | 2,015198 |
| T1 | ds-U16-calyx-R1 |  |  | 18,4613355 | 18,9284962 | 4,363722 |
| T2 | ds-U16-calyx-R2 |  |  | 17,1719372 | 19,3049705 | 12,561844 |
| T3 | ds-U16-calyx-R3 |  |  | 21,0413776 | 20,4723268 | 2,526406 |
| T4 | ds-U16-calyx-R4 |  |  | 20,6009788 | 19,6629592 | 1,910227 |
| T1 | ds-U16-hind legs-R1 |  |  | 20,115247 | 19,5191085 | 2,340564 |
| T2 | ds-U16-hind legs-R2 |  |  | 20,4522977 | 19,6603043 | 2,091072 |
| T3 | ds-U16-hind legs-R3 |  |  | 20,5126315 | 19,7922 | 2,204129 |
| T4 | ds-U16-hind legs-R4 |  |  | 19,5180776 | 18,6709878 | 1,899580 |
| C1 | ds-GFP-calyx-R1 |  |  | 16,7401228 | 19,0152365 | 3,998340 |
| C2 | ds-GFP-calyx-R2 |  |  | 17,6187128 | 20,1914827 | 4,856786 |
| C3 | ds-GFP-calyx-R3 |  |  | 20,0497645 | 22,0709032 | 3,251533 |
| C4 | ds-GFP-calyx-R4 |  |  | 17,9938592 | 20,0149517 | 3,319312 |
| C1 | ds-GFP-hind legs-R1 |  |  | 19,1757234 | 18,0663004 | 0,386522 |
| C2 | ds-GFP-hind legs-R2 |  |  | 20,6845894 | 20,0652367 | 0,532078 |
| C3 | ds-GFP-hind legs-R3 |  |  | 23,4797017 | 22,3636463 | 0,368486 |
| C4 | ds-GFP-hind legs-R4 | *U34* | 2 | 21,7367545 | 20,3911466 | 0,320574 |
| T1 | ds-U16-calyx-R1 |  |  | 19,5041775 | 18,9284962 | 0,554732 |
| T2 | ds-U16-calyx-R2 |  |  | 18,7712267 | 19,3049705 | 1,192368 |
| T3 | ds-U16-calyx-R3 |  |  | 21,5464526 | 20,4723268 | 0,386632 |
| T4 | ds-U16-calyx-R4 |  |  | 20,7905141 | 19,6629592 | 0,375619 |
| T1 | ds-U16-hind legs-R1 |  |  | 20,3347818 | 19,5191085 | 0,466940 |
| T2 | ds-U16-hind legs-R2 |  |  | 20,0156603 | 19,6603043 | 0,641525 |
| T3 | ds-U16-hind legs-R3 |  |  | 20,8052665 | 19,7922 | 0,406114 |
| T4 | ds-U16-hind legs-R4 |  |  | 19,9173643 | 18,6709878 | 0,349388 |
| C1 | ds-GFP-calyx-R1 |  |  | 17,1786901 | 19,0152365 | 7,796633 |
| C2 | ds-GFP-calyx-R2 |  |  | 18,1769983 | 20,1914827 | 9,222832 |
| C3 | ds-GFP-calyx-R3 |  |  | 20,7087837 | 22,0709032 | 6,644786 |
| C4 | ds-GFP-calyx-R4 |  |  | 18,0886302 | 20,0149517 | 8,648168 |
| C1 | ds-GFP-hind legs-R1 |  |  | 18,2028323 | 18,0663004 | 2,124490 |
| C2 | ds-GFP-hind legs-R2 |  |  | 20,2154306 | 20,0652367 | 2,311335 |
| C3 | ds-GFP-hind legs-R3 |  |  | 22,6318174 | 22,3636463 | 2,386065 |
| C4 | ds-GFP-hind legs-R4 | *U36* | 1,89 | 20,4956732 | 20,3911466 | 2,415865 |
| T1 | ds-U16-calyx-R1 |  |  | 18,9664324 | 18,9284962 | 2,354680 |
| T2 | ds-U16-calyx-R2 |  |  | 19,0690543 | 19,3049705 | 2,852644 |
| T3 | ds-U16-calyx-R3 |  |  | 20,6651108 | 20,4723268 | 2,292519 |
| T4 | ds-U16-calyx-R4 |  |  | 19,7079242 | 19,6629592 | 2,425645 |
| T1 | ds-U16-hind legs-R1 |  |  | 19,6359059 | 19,5191085 | 2,301771 |
| T2 | ds-U16-hind legs-R2 |  |  | 19,8354646 | 19,6603043 | 2,232439 |
| T3 | ds-U16-hind legs-R3 |  |  | 19,9918492 | 19,7922 | 2,211434 |
| T4 | ds-U16-hind legs-R4 |  |  | 18,7996934 | 18,6709878 | 2,196013 |
| C1 | ds-GFP-calyx-R1 |  |  | 14,6795147 | 19,0152365 | 20,822720 |
| C2 | ds-GFP-calyx-R2 |  |  | 15,5143503 | 20,1914827 | 26,403279 |
| C3 | ds-GFP-calyx-R3 |  |  | 18,2500707 | 22,0709032 | 14,915549 |
| C4 | ds-GFP-calyx-R4 |  |  | 15,7229344 | 20,0149517 | 20,317313 |
| C1 | ds-GFP-hind legs-R1 |  |  | 17,8873939 | 18,0663004 | 1,237112 |
| C2 | ds-GFP-hind legs-R2 |  |  | 19,7120826 | 20,0652367 | 1,406419 |
| C3 | ds-GFP-hind legs-R3 |  |  | 22,0660588 | 22,3636463 | 1,370271 |
| C4 | ds-GFP-hind legs-R4 | *U37* | 1,97 | 20,0864536 | 20,3911466 | 1,363205 |
| T1 | ds-U16-calyx-R1 |  |  | 18,3139797 | 18,9284962 | 1,669465 |
| T2 | ds-U16-calyx-R2 |  |  | 17,6137725 | 19,3049705 | 3,470976 |
| T3 | ds-U16-calyx-R3 |  |  | 20,1865329 | 20,4723268 | 1,346401 |
| T4 | ds-U16-calyx-R4 |  |  | 19,4773388 | 19,6629592 | 1,252844 |
| T1 | ds-U16-hind legs-R1 |  |  | 19,280554 | 19,5191085 | 1,297681 |
| T2 | ds-U16-hind legs-R2 |  |  | 19,3066047 | 19,6603043 | 1,404058 |
| T3 | ds-U16-hind legs-R3 |  |  | 19,5816372 | 19,7922 | 1,275046 |
| T4 | ds-U16-hind legs-R4 |  |  | 18,4742987 | 18,6709878 | 1,255958 |
| C1 | ds-GFP-calyx-R1 |  |  | 18,8221547 | 19,0152365 | 2,036194 |
| C2 | ds-GFP-calyx-R2 |  |  | 20,1599131 | 20,1914827 | 1,900124 |
| C3 | ds-GFP-calyx-R3 |  |  | 22,2766225 | 22,0709032 | 1,724543 |
| C4 | ds-GFP-calyx-R4 |  |  | 20,235283 | 20,0149517 | 1,603463 |
| C1 | ds-GFP-hind legs-R1 |  |  | 19,9064164 | 18,0663004 | 0,524965 |
| C2 | ds-GFP-hind legs-R2 |  |  | 21,8139498 | 20,0652367 | 0,592568 |
| C3 | ds-GFP-hind legs-R3 |  |  | 24,366736 | 22,3636463 | 0,538752 |
| C4 | ds-GFP-hind legs-R4 | *XRCC1* | 1,92 | 22,5439325 | 20,3911466 | 0,459854 |
| T1 | ds-U16-calyx-R1 |  |  | 20,7160103 | 18,9284962 | 0,557898 |
| T2 | ds-U16-calyx-R2 |  |  | 20,7531489 | 19,3049705 | 0,704239 |
| T3 | ds-U16-calyx-R3 |  |  | 22,5257937 | 20,4723268 | 0,491860 |
| T4 | ds-U16-calyx-R4 |  |  | 21,6360812 | 19,6629592 | 0,505577 |
| T1 | ds-U16-hind legs-R1 |  |  | 21,2312012 | 19,5191085 | 0,596781 |
| T2 | ds-U16-hind legs-R2 |  |  | 21,270179 | 19,6603043 | 0,640708 |
| T3 | ds-U16-hind legs-R3 |  |  | 21,8092887 | 19,7922 | 0,493240 |
| T4 | ds-U16-hind legs-R4 |  |  | 20,7246009 | 18,6709878 | 0,465294 |
| C1 | ds-GFP-calyx-R1 |  |  | 14,5342312 | 19,0152365 | 18,446723 |
| C2 | ds-GFP-calyx-R2 |  |  | 16,245975 | 20,1914827 | 12,577209 |
| C3 | ds-GFP-calyx-R3 |  |  | 17,5944284 | 22,0709032 | 17,832736 |
| C4 | ds-GFP-calyx-R4 |  |  | 15,7700937 | 20,0149517 | 15,504873 |
| C1 | ds-GFP-hind legs-R1 |  |  | 19,5017225 | 18,0663004 | 0,308346 |
| C2 | ds-GFP-hind legs-R2 |  |  | 21,4619379 | 20,0652367 | 0,310435 |
| C3 | ds-GFP-hind legs-R3 |  |  | 23,7064091 | 22,3636463 | 0,314902 |
| C4 | ds-GFP-hind legs-R4 | *Vank1_Hd24* | 2 | 21,8673836 | 20,3911466 | 0,292823 |
| T1 | ds-U16-calyx-R1 |  |  | 19,8238757 | 18,9284962 | 0,444472 |
| T2 | ds-U16-calyx-R2 |  |  | 19,4935171 | 19,3049705 | 0,722735 |
| T3 | ds-U16-calyx-R3 |  |  | 22,4043781 | 20,4723268 | 0,213323 |
| T4 | ds-U16-calyx-R4 |  |  | 21,0007813 | 19,6629592 | 0,324676 |
| T1 | ds-U16-hind legs-R1 |  |  | 20,8180884 | 19,5191085 | 0,334019 |
| T2 | ds-U16-hind legs-R2 |  |  | 20,9581306 | 19,6603043 | 0,333812 |
| T3 | ds-U16-hind legs-R3 |  |  | 21,1234835 | 19,7922 | 0,325728 |
| T4 | ds-U16-hind legs-R4 |  |  | 20,1422937 | 18,6709878 | 0,298949 |
| C1 | ds-GFP-calyx-R1 |  |  | 13,7300216 | 19,0152365 | 39,639769 |
| C2 | ds-GFP-calyx-R2 |  |  | 15,1193292 | 20,1914827 | 34,512645 |
| C3 | ds-GFP-calyx-R3 |  |  | 16,8592716 | 22,0709032 | 38,298573 |
| C4 | ds-GFP-calyx-R4 |  |  | 14,8940942 | 20,0149517 | 35,639512 |
| C1 | ds-GFP-hind legs-R1 |  |  | 19,6893146 | 18,0663004 | 0,364590 |
| C2 | ds-GFP-hind legs-R2 |  |  | 21,3900932 | 20,0652367 | 0,450814 |
| C3 | ds-GFP-hind legs-R3 |  |  | 23,8808219 | 22,3636463 | 0,400332 |
| C4 | ds-GFP-hind legs-R4 | *Vank1_Hd43* | 1,97 | 21,9608211 | 20,3911466 | 0,382493 |
| T1 | ds-U16-calyx-R1 |  |  | 19,9197621 | 18,9284962 | 0,561990 |
| T2 | ds-U16-calyx-R2 |  |  | 18,9090334 | 19,3049705 | 1,442252 |
| T3 | ds-U16-calyx-R3 |  |  | 22,0299498 | 20,4723268 | 0,385790 |
| T4 | ds-U16-calyx-R4 |  |  | 21,0646269 | 19,6629592 | 0,427065 |
| T1 | ds-U16-hind legs-R1 |  |  | 21,0909014 | 19,5191085 | 0,380261 |
| T2 | ds-U16-hind legs-R2 |  |  | 20,9270454 | 19,6603043 | 0,467972 |
| T3 | ds-U16-hind legs-R3 |  |  | 21,3911213 | 19,7922 | 0,373847 |
| T4 | ds-U16-hind legs-R4 |  |  | 20,2850354 | 18,6709878 | 0,367938 |
| C1 | ds-GFP-calyx-R1 |  |  | 16,7671135 | 19,0152365 | 3,924232 |
| C2 | ds-GFP-calyx-R2 |  |  | 18,9896538 | 20,1914827 | 1,877823 |
| C3 | ds-GFP-calyx-R3 |  |  | 19,9846321 | 22,0709032 | 3,401692 |
| C4 | ds-GFP-calyx-R4 |  |  | 18,1083248 | 20,0149517 | 3,066130 |
| C1 | ds-GFP-hind legs-R1 |  |  | 20,3434917 | 18,0663004 | 0,172045 |
| C2 | ds-GFP-hind legs-R2 |  |  | 22,8676699 | 20,0652367 | 0,117166 |
| C3 | ds-GFP-hind legs-R3 |  |  | 24,8783341 | 22,3636463 | 0,139762 |
| C4 | ds-GFP-hind legs-R4 | *Vank1_Hd28* | 2 | 22,8457831 | 20,3911466 | 0,148620 |
| T1 | ds-U16-calyx-R1 |  |  | 21,1441948 | 18,9284962 | 0,177987 |
| T2 | ds-U16-calyx-R2 |  |  | 21,7419386 | 19,3049705 | 0,152103 |
| T3 | ds-U16-calyx-R3 |  |  | 23,065406 | 20,4723268 | 0,134911 |
| T4 | ds-U16-calyx-R4 |  |  | 21,9941027 | 19,6629592 | 0,163091 |
| T1 | ds-U16-hind legs-R1 |  |  | 21,7313826 | 19,5191085 | 0,177354 |
| T2 | ds-U16-hind legs-R2 |  |  | 22,4387791 | 19,6603043 | 0,119614 |
| T3 | ds-U16-hind legs-R3 |  |  | 22,2148 | 19,7922 | 0,152875 |
| T4 | ds-U16-hind legs-R4 |  |  | 20,9980577 | 18,6709878 | 0,165191 |
| C1 | ds-GFP-calyx-R1 |  |  | 11,9102287 | 19,0152365 | 113,716288 |
| C2 | ds-GFP-calyx-R2 |  |  | 14,8806947 | 20,1914827 | 32,402207 |
| C3 | ds-GFP-calyx-R3 |  |  | 17,4535673 | 22,0709032 | 19,661715 |
| C4 | ds-GFP-calyx-R4 |  |  | 14,4374694 | 20,0149517 | 39,050636 |
| C1 | ds-GFP-hind legs-R1 |  |  | 18,9173442 | 18,0663004 | 0,462332 |
| C2 | ds-GFP-hind legs-R2 |  |  | 23,0396953 | 20,0652367 | 0,103996 |
| C3 | ds-GFP-hind legs-R3 |  |  | 25,6468159 | 22,3636463 | 0,082045 |
| C4 | ds-GFP-hind legs-R4 | *M24* | 2 | 22,5638677 | 20,3911466 | 0,180693 |
| T1 | ds-U16-calyx-R1 |  |  | 18,0435323 | 18,9284962 | 1,526798 |
| T2 | ds-U16-calyx-R2 |  |  | 18,2569418 | 19,3049705 | 1,703043 |
| T3 | ds-U16-calyx-R3 |  |  | 23,5960403 | 20,4723268 | 0,093392 |
| T4 | ds-U16-calyx-R4 |  |  | 22,1731758 | 19,6629592 | 0,144054 |
| T1 | ds-U16-hind legs-R1 |  |  | 20,5003942 | 19,5191085 | 0,416300 |
| T2 | ds-U16-hind legs-R2 |  |  | 22,3217039 | 19,6603043 | 0,129726 |
| T3 | ds-U16-hind legs-R3 |  |  | 22,8229421 | 19,7922 | 0,100292 |
| T4 | ds-U16-hind legs-R4 |  |  | 20,7556333 | 18,6709878 | 0,195418 |
| C1 | ds-GFP-calyx-R1 |  |  | 13,615454 | 19,0152365 | 45,915537 |
| C2 | ds-GFP-calyx-R2 |  |  | 16,1067961 | 20,1914827 | 19,177903 |
| C3 | ds-GFP-calyx-R3 |  |  | 16,8148581 | 22,0709032 | 42,995631 |
| C4 | ds-GFP-calyx-R4 |  |  | 14,8713188 | 20,0149517 | 39,039679 |
| C1 | ds-GFP-hind legs-R1 |  |  | 19,0363104 | 18,0663004 | 0,625410 |
| C2 | ds-GFP-hind legs-R2 |  |  | 22,3449544 | 20,0652367 | 0,264368 |
| C3 | ds-GFP-hind legs-R3 |  |  | 23,8354939 | 22,3636463 | 0,466066 |
| C4 | ds-GFP-hind legs-R4 | *P30_Hd6* | 1,96 | 21,8089384 | 20,3911466 | 0,473749 |
| T1 | ds-U16-calyx-R1 |  |  | 19,2335556 | 18,9284962 | 0,986967 |
| T2 | ds-U16-calyx-R2 |  |  | 19,9816108 | 19,3049705 | 0,771553 |
| T3 | ds-U16-calyx-R3 |  |  | 21,9590705 | 20,4723268 | 0,452642 |
| T4 | ds-U16-calyx-R4 |  |  | 20,9358354 | 19,6629592 | 0,518430 |
| T1 | ds-U16-hind legs-R1 |  |  | 20,4821002 | 19,5191085 | 0,637708 |
| T2 | ds-U16-hind legs-R2 |  |  | 21,9296277 | 19,6603043 | 0,265131 |
| T3 | ds-U16-hind legs-R3 |  |  | 21,2432927 | 19,7922 | 0,460443 |
| T4 | ds-U16-hind legs-R4 |  |  | 19,9739276 | 18,6709878 | 0,502957 |

**Table D.** Genomic DNA amplification (Cf Fig 8). Statistical analyses.

| **DEVrep relative amplification –**  **Comparison between dsGFP vs dsU16** | | **Multiple comparisons of means** | | | | |
| --- | --- | --- | --- | --- | --- | --- |
| **Genes** | **Condition** | Mean difference | Std error | t | P value | Significance |
| ***H1*** | **dsGFP calyx vs dsU16 calyx** | -0,0044 | 0,079 | -0,055 | 1 | n.s |
|  | **dsGFP calyx vs dsGFP hind legs** | 0,033 | 0,079 | 0,42 | 0,97 | n.s |
|  | **dsGFP calyx vs dsU16 hind legs** | 0,038 | 0,079 | 0,48 | 0,96 | n.s |
|  | **dsU16 calyx vs dsGFP hind legs** | 0,038 | 0,079 | 0,47 | 0,96 | n.s |
|  | **dsU16 calyx vs dsU16 hind legs** | 0,043 | 0,079 | 0,54 | 0,94 | n.s |
|  | **dsU16 hind legs vs dsGFP hind legs** | 0,00048 | 0,079 | 0,061 | 1 | n.s |
| ***Rpl*** | **dsGFP calyx vs dsU16 calyx** | 0,13 | 0,16 | 0,79 | 0,85 | n.s |
|  | **dsGFP calyx vs dsGFP hind legs** | 0,064 | 0,16 | 0,38 | 0,98 | n.s |
|  | **dsGFP calyx vs dsU16 hind legs** | -0,045 | 0,16 | -0,27 | 0,99 | n.s |
|  | **dsU16 calyx vs dsGFP hind legs** | -0,067 | 0,16 | -0,4 | 0,97 | n.s |
|  | **dsU16 calyx vs dsU16 hind legs** | -0,17 | 0,16 | -1,06 | 0,71 | n.s |
|  | **dsU16 hind legs vs dsGFP hind legs** | -0,1 | 0,16 | -0,65 | 0,91 | n.s |
| ***IVp53-1*** | **dsGFP calyx vs dsU16 calyx** | -67,32 | 2,9 | -23,14 | <0,0001 | *** |
|  | **dsGFP calyx vs dsGFP hind legs** | -69,14 | 2,9 | -23,77 | <0,0001 | *** |
|  | **dsGFP calyx vs dsU16 hind legs** | -69,05 | 2,9 | -23,74 | <0,0001 | *** |
|  | **dsU16 calyx vs dsGFP hind legs** | -1,81 | 2,9 | -0,622 | 0,923 | n.s |
|  | **dsU16 calyx vs dsU16 hind legs** | -1,73 | 2,9 | -0,59 | 0,932 | n.s |
|  | **dsU16 hind legs vs dsGFP hind legs** | 0,081 | 2,9 | 0,028 | 1 | n.s |
| ***IVp53-2*** | **dsGFP calyx vs dsU16 calyx** | -12,74 | 1,69 | -7,52 | <0,0001 | *** |
|  | **dsGFP calyx vs dsGFP hind legs** | -13,07 | 1,69 | -7,72 | <0,0001 | *** |
|  | **dsGFP calyx vs dsU16 hind legs** | -13,04 | 1,69 | -7,7 | <0,0001 | *** |
|  | **dsU16 calyx vs dsGFP hind legs** | -0,33 | 1,69 | -0,19 | 0,99 | n.s |
|  | **dsU16 calyx vs dsU16 hind legs** | -0,3 | 1,69 | -0,17 | 0,99 | n.s |
|  | **dsU16 hind legs vs dsGFP hind legs** | 0,033 | 1,69 | 0,02 | 1 | n.s |
| ***IVp12-1*** | **dsGFP calyx vs dsU16 calyx** | -44,14 | 1,96 | -22,49 | <0,0001 | *** |
|  | **dsGFP calyx vs dsGFP hind legs** | -45,3 | 1,96 | -23,07 | <0,0001 | *** |
|  | **dsGFP calyx vs dsU16 hind legs** | -45,33 | 1,96 | -23,09 | <0,0001 | *** |
|  | **dsU16 calyx vs dsGFP hind legs** | -1,15 | 1,96 | -0,58 | 0,93 | n.s |
|  | **dsU16 calyx vs dsU16 hind legs** | -1,18 | 1,96 | -0,6 | 0,92 | n.s |
|  | **dsU16 hind legs vs dsGFP hind legs** | 0,037 | 1,96 | -0,019 | 1 | n.s |
| ***IVSP3-1*** | **dsGFP calyx vs dsU16 calyx** | -43,99 | 2,18 | -20,15 | <0,0001 | *** |
|  | **dsGFP calyx vs dsGFP hind legs** | -45,32 | 2,18 | -20,76 | <0,0001 | *** |
|  | **dsGFP calyx vs dsU16 hind legs** | -45,33 | 2,18 | -20,77 | <0,0001 | *** |
|  | **dsU16 calyx vs dsGFP hind legs** | -1,33 | 2,18 | -0,61 | 0,92 | n.s |
|  | **dsU16 calyx vs dsU16 hind legs** | -1,34 | 2,18 | -0,61 | 0,92 | n.s |
|  | **dsU16 hind legs vs dsGFP hind legs** | -0,0086 | 2,18 | -0,004 | 1 | n.s |
| ***U11*** | **dsGFP calyx vs dsU16 calyx** | -50,02 | 3,76 | -13,3 | <0,0001 | *** |
|  | **dsGFP calyx vs dsGFP hind legs** | -51,86 | 3,76 | -13,79 | <0,0001 | *** |
|  | **dsGFP calyx vs dsU16 hind legs** | 51,89 | 3,76 | -13,79 | <0,0001 | *** |
|  | **dsU16 calyx vs dsGFP hind legs** | -1,84 | 3,76 | -0,49 | 0,96 | n.s |
|  | **dsU16 calyx vs dsU16 hind legs** | -1,86 | 3,76 | -0,49 | 0,95 | n.s |
|  | **dsU16 hind legs vs dsGFP hind legs** | -0,022 | 3,76 | -0,006 | 1 | n.s |
| ***U16*** | **dsGFP calyx vs dsU16 calyx** | -67,14 | 4,35 | -15,43 | <0,0001 | *** |
|  | **dsGFP calyx vs dsGFP hind legs** | -69,05 | 4,35 | -15,87 | <0,0001 | *** |
|  | **dsGFP calyx vs dsU16 hind legs** | -69,05 | 4,35 | -15,87 | <0,0001 | *** |
|  | **dsU16 calyx vs dsGFP hind legs** | -1,91 | 4,35 | -0,43 | 0,97 | n.s |
|  | **dsU16 calyx vs dsU16 hind legs** | -1,91 | 4,35 | -0,43 | 0,97 | n.s |
|  | **dsU16 hind legs vs dsGFP hind legs** | 0,00023 | 4,35 | 0 | 1 | n.s |
| ***U22*** | **dsGFP calyx vs dsU16 calyx** | -90,31 | 3,91 | -23,04 | <0,0001 | *** |
|  | **dsGFP calyx vs dsGFP hind legs** | -92,79 | 3,91 | -23,67 | <0,0001 | *** |
|  | **dsGFP calyx vs dsU16 hind legs** | -92,81 | 3,91 | -23,68 | <0,0001 | *** |
|  | **dsU16 calyx vs dsGFP hind legs** | -2,48 | 3,91 | -0,63 | 0,91 | n.s |
|  | **dsU16 calyx vs dsU16 hind legs** | -2,49 | 3,91 | -0,63 | 0,91 | n.s |
|  | **dsU16 hind legs vs dsGFP hind legs** | -0,018 | 3,91 | -0,005 | 1 | n.s |
| ***U23*** | **dsGFP calyx vs dsU16 calyx** | -110,86 | 5,58 | -19,87 | <0,0001 | *** |
|  | **dsGFP calyx vs dsGFP hind legs** | -113,98 | 5,58 | -20,42 | <0,0001 | *** |
|  | **dsGFP calyx vs dsU16 hind legs** | -114,06 | 5,58 | -20,44 | <0,0001 | *** |
|  | **dsU16 calyx vs dsGFP hind legs** | 3,12 | 5,58 | -0,56 | 0,94 | n.s |
|  | **dsU16 calyx vs dsU16 hind legs** | 3,2 | 5,58 | -0,57 | 0,93 | n.s |
|  | **dsU16 hind legs vs dsGFP hind legs** | -0,083 | 5,58 | -0,015 | 1 | n.s |
| ***U34*** | **dsGFP calyx vs dsU16 calyx** | -3,22 | 0,3 | -10,68 | <0,0001 | *** |
|  | **dsGFP calyx vs dsGFP hind legs** | -3,45 | 0,3 | -11,42 | <0,0001 | *** |
|  | **dsGFP calyx vs dsU16 hind legs** | -3,39 | 0,3 | -11,21 | <0,0001 | *** |
|  | **dsU16 calyx vs dsGFP hind legs** | -0,22 | 0,3 | -0,74 | 0,87 | n.s |
|  | **dsU16 calyx vs dsU16 hind legs** | -0,16 | 0,3 | -0,53 | 0,94 | n.s |
|  | **dsU16 hind legs vs dsGFP hind legs** | 0,064 | 0,3 | 0,21 | 0,99 | n.s |
| ***U36*** | **dsGFP calyx vs dsU16 calyx** | -5,59 | 0,4 | -13,67 | <0,0001 | *** |
|  | **dsGFP calyx vs dsGFP hind legs** | -5,76 | 0,4 | -14,09 | <0,0001 | *** |
|  | **dsGFP calyx vs dsU16 hind legs** | -5,84 | 0,4 | -14,27 | <0,0001 | *** |
|  | **dsU16 calyx vs dsGFP hind legs** | -0,17 | 0,4 | -0,42 | 0,97 | n.s |
|  | **dsU16 calyx vs dsU16 hind legs** | -0,24 | 0,4 | -0,6 | 0,93 | n.s |
|  | **dsU16 hind legs vs dsGFP hind legs** | -0,074 | 0,4 | -0,18 | 0,99 | n.s |
| ***U37*** | **dsGFP calyx vs dsU16 calyx** | -18,67 | 1,7 | -10,98 | <0,0001 | *** |
|  | **dsGFP calyx vs dsGFP hind legs** | -19,27 | 1,7 | -11,33 | <0,0001 | *** |
|  | **dsGFP calyx vs dsU16 hind legs** | -19,3 | 1,7 | -11,35 | <0,0001 | *** |
|  | **dsU16 calyx vs dsGFP hind legs** | -0,59 | 1,7 | -0,34 | 0,98 | n.s |
|  | **dsU16 calyx vs dsU16 hind legs** | -0,62 | 1,7 | -0,36 | 0,98 | n.s |
|  | **dsU16 hind legs vs dsGFP hind legs** | -0,036 | 1,7 | *-0,021* | 1 | n.s |
| ***XRCC1*** | **dsGFP calyx vs dsU16 calyx** | -1,25 | 0,083 | -14,99 | <0,0001 | *** |
|  | **dsGFP calyx vs dsGFP hind legs** | -1,28 | 0,083 | -15,42 | <0,0001 | *** |
|  | **dsGFP calyx vs dsU16 hind legs** | -1,26 | 0,083 | -15,18 | <0,0001 | *** |
|  | **dsU16 calyx vs dsGFP hind legs** | -0,035 | 0,083 | -0,43 | 0,97 | n.s |
|  | **dsU16 calyx vs dsU16 hind legs** | -0,015 | 0,083 | -0,19 | 0,99 | n.s |
|  | **dsU16 hind legs vs dsGFP hind legs** | 0,019 | 0,083 | 0,23 | 0,99 | n.s |

| **DNA segment genes relative amplification - comparison between dsGFP vs dsU16** | | **Multiple comparisons of means** | | | | |
| --- | --- | --- | --- | --- | --- | --- |
| **Genes** | **Condition** | Mean difference | Std error | t | P value | Significance |
| ***Vank1_ Hd24*** | **dsGFP calyx vs dsU16 calyx** | -15,66 | 0,94 | -16,6 | <0,0001 | *** |
|  | **dsGFP calyx vs dsGFP hind legs** | -15,78 | 0,94 | -16,7 | <0,0001 | *** |
|  | **dsGFP calyx vs dsU16 hind legs** | -15,76 | 0,94 | -16,7 | <0,0001 | *** |
|  | **dsU16 calyx vs dsGFP hind legs** | -0,11 | 0,94 | -0,12 | 0,99 | n.s |
|  | **dsU16 calyx vs dsU16 hind legs** | -0,1 | 0,94 | -0,1 | 1 | n.s |
|  | **dsU16 hind legs vs dsGFP hind legs** | 0,016 | 0,94 | 0,017 | 1 | n.s |
| ***Vank1_ Hd43*** | **dsGFP calyx vs dsU16 calyx** | -36,31 | 0,85 | -42,6 | <0,0001 | *** |
|  | **dsGFP calyx vs dsGFP hind legs** | -36,62 | 0,85 | -46 | <0,0001 | *** |
|  | **dsGFP calyx vs dsU16 hind legs** | -36,62 | 0,85 | -43 | <0,0001 | *** |
|  | **dsU16 calyx vs dsGFP hind legs** | -0,3 | 0,85 | -0,35 | 0,98 | n.s |
|  | **dsU16 calyx vs dsU16 hind legs** | -0,3 | 0,85 | -0,36 | 0,98 | n.s |
|  | **dsU16 hind legs vs dsGFP hind legs** | -0,002 | 0,85 | -0 | 1 | n.s |
| ***Vank1_ Hd28*** | **dsGFP calyx vs dsU16 calyx** | -2,91 | 0,3 | -9,47 | <0,0001 | *** |
|  | **dsGFP calyx vs dsGFP hind legs** | -2,92 | 0,3 | -9,51 | <0,0001 | *** |
|  | **dsGFP calyx vs dsU16 hind legs** | -2,91 | 0,3 | -9,48 | <0,0001 | *** |
|  | **dsU16 calyx vs dsGFP hind legs** | -0,012 | 0,3 | -0,04 | 1 | n.s |
|  | **dsU16 calyx vs dsU16 hind legs** | -0,0032 | 0,3 | -0,01 | 1 | n.s |
|  | **dsU16 hind legs vs dsGFP hind legs** | 0,0093 | 0,3 | 0,03 | 1 | n.s |
| ***M24*** | **dsGFP calyx vs dsU16 calyx** | -50,34 | 15,008 | -3,35 | 0,0256 | * |
|  | **dsGFP calyx vs dsGFP hind legs** | -51,00044 | 15,008 | -3,39 | 0,0236 | * |
|  | **dsGFP calyx vs dsU16 hind legs** | -50,99 | 15,008 | -3,39 | 0,0238 | * |
|  | **dsU16 calyx vs dsGFP hind legs** | -0,65 | 15,008 | -0,04 | 1 | n.s |
|  | **dsU16 calyx vs dsU16 hind legs** | -0,65 | 15,008 | -0,04 | 1 | n.s |
|  | **dsU16 hind legs vs dsGFP hind legs** | 0,0031 | 15,008 | 0 | 1 | n.s |
| ***P30_Hd6*** | **dsGFP calyx vs dsU16 calyx** | -36,09 | 4,26 | -8,45 | <0,0001 | *** |
|  | **dsGFP calyx vs dsGFP hind legs** | -36,32 | 4,26 | -8,5 | <0,0001 | *** |
|  | **dsGFP calyx vs dsU16 hind legs** | -36,31 | 4,26 | -8,5 | <0,0001 | *** |
|  | **dsU16 calyx vs dsGFP hind legs** | -0,22 | 4,26 | -0,05 | 1 | n.s |
|  | **dsU16 calyx vs dsU16 hind legs** | -0,21 | 4,26 | -0,05 | 1 | n.s |
|  | **dsU16 hind legs vs dsGFP hind legs** | 0,0091 | 4,26 | 0,002 | 1 | n.s |

**RNA quantification of IVSPER genes in ds*GFP* and ds*U16*-injected wasps**

**Table E.** RNA quantification (Cf Fig 9A). Raw data.

| Biological replica ID | Sample | Target gene | Efficiency | Mean Ct Target gene | Mean Ct reference (ELF-1) | Relative expression to ELF-1 |
| --- | --- | --- | --- | --- | --- | --- |
| C1 | ds-GFP | CCO | 1,93 | 18,8856007 | 16,7441326 | 0,375400 |
| C2 | ds-GFP | CCO |  | 19,1679176 | 16,5891812 | 0,280500 |
| C3 | ds-GFP | CCO |  | 19,0064407 | 16,4614331 | 0,285800 |
| C4 | ds-GFP | CCO |  | 19,0191892 | 16,5955505 | 0,310600 |
| T1 | ds-U16 | CCO |  | 18,6942765 | 15,8012524 | 0,223600 |
| T2 | ds-U16 | CCO |  | 18,7606443 | 16,200272 | 0,281100 |
| T3 | ds-U16 | CCO |  | 18,9492907 | 16,1651763 | 0,242400 |
| T4 | ds-U16 | CCO |  | 19,6589699 | 16,7928027 | 0,233400 |
| T5 | ds-U16 | CCO |  | 18,8055348 | 16,3985105 | 0,312500 |
| T6 | ds-U16 | CCO |  | 18,813601 | 16,124396 | 0,257700 |
| T7 | ds-U16 | CCO |  | 18,8086081 | 16,3187396 | 0,295300 |
| T8 | ds-U16 | CCO |  | 19,0560037 | 16,5495418 | 0,293800 |
| C1 | ds-GFP | IVp53-1 | 1,93 | 19,6268949 | 16,7441326 | 0,230600 |
| C2 | ds-GFP | IVp53-1 |  | 19,3120372 | 16,5891812 | 0,255100 |
| C3 | ds-GFP | IVp53-1 |  | 18,7350186 | 16,4614331 | 0,341700 |
| C4 | ds-GFP | IVp53-1 |  | 18,9757192 | 16,5955505 | 0,319600 |
| T1 | ds-U16 | IVp53-1 |  | 28,3702391 | 15,8012524 | 0,000386 |
| T2 | ds-U16 | IVp53-1 |  | 28,7336676 | 16,200272 | 0,000399 |
| T3 | ds-U16 | IVp53-1 |  | 25,8533612 | 16,1651763 | 0,002590 |
| T4 | ds-U16 | IVp53-1 |  | 24,6083494 | 16,7928027 | 0,009010 |
| T5 | ds-U16 | IVp53-1 |  | 26,757121 | 16,3985105 | 0,001680 |
| T6 | ds-U16 | IVp53-1 |  | 23,8279802 | 16,124396 | 0,009530 |
| T7 | ds-U16 | IVp53-1 |  | 25,6762068 | 16,3187396 | 0,003230 |
| T8 | ds-U16 | IVp53-1 |  | 28,1148128 | 16,5495418 | 0,000761 |
| C1 | ds-GFP | IVp53-2 | 1,75 | 22,4800735 | 16,7441326 | 0,319100 |
| C2 | ds-GFP | IVp53-2 |  | 21,8351031 | 16,5891812 | 0,411800 |
| C3 | ds-GFP | IVp53-2 |  | 21,1594168 | 16,4614331 | 0,550800 |
| C4 | ds-GFP | IVp53-2 |  | 21,4038451 | 16,5955505 | 0,526500 |
| T1 | ds-U16 | IVp53-2 |  | 32,7004063 | 15,8012524 | 0,000550 |
| T2 | ds-U16 | IVp53-2 |  | 32,5632261 | 16,200272 | 0,000780 |
| T3 | ds-U16 | IVp53-2 |  | 31,0422918 | 16,1651763 | 0,001780 |
| T4 | ds-U16 | IVp53-2 |  | 31,2236907 | 16,7928027 | 0,002470 |
| T5 | ds-U16 | IVp53-2 |  | 31,3648832 | 16,3985105 | 0,001750 |
| T6 | ds-U16 | IVp53-2 |  | 28,9347979 | 16,124396 | 0,005640 |
| T7 | ds-U16 | IVp53-2 |  | 31,8777827 | 16,3187396 | 0,001240 |
| T8 | ds-U16 | IVp53-2 |  | 32,1122293 | 16,5495418 | 0,001270 |
| C1 | ds-GFP | IVSP2-2 | 2 | 23,9659345 | 16,7441326 | 0,005660 |
| C2 | ds-GFP | IVSP2-2 |  | 23,7550588 | 16,5891812 | 0,005890 |
| C3 | ds-GFP | IVSP2-2 |  | 22,7861246 | 16,4614331 | 0,010600 |
| C4 | ds-GFP | IVSP2-2 |  | 22,9955432 | 16,5955505 | 0,010000 |
| T1 | ds-U16 | IVSP2-2 |  | 31,4636199 | 15,8012524 | 0,000017 |
| T2 | ds-U16 | IVSP2-2 |  | 32,444937 | 16,200272 | 0,000011 |
| T3 | ds-U16 | IVSP2-2 |  | 30,6768262 | 16,1651763 | 0,000036 |
| T4 | ds-U16 | IVSP2-2 |  | 30,7168148 | 16,7928027 | 0,000054 |
| T5 | ds-U16 | IVSP2-2 |  | 29,2711486 | 16,3985105 | 0,000113 |
| T6 | ds-U16 | IVSP2-2 |  | 26,806765 | 16,124396 | 0,000517 |
| T7 | ds-U16 | IVSP2-2 |  | 29,0787185 | 16,3187396 | 0,000122 |
| T8 | ds-U16 | IVSP2-2 |  | 31,2778272 | 16,5495418 | 0,000031 |
| C1 | ds-GFP | IVSP3-1 | 1,94 | 20,7496123 | 16,7441326 | 0,099000 |
| C2 | ds-GFP | IVSP3-1 |  | 20,5753962 | 16,5891812 | 0,100000 |
| C3 | ds-GFP | IVSP3-1 |  | 19,9655557 | 16,4614331 | 0,137200 |
| C4 | ds-GFP | IVSP3-1 |  | 20,0786811 | 16,5955505 | 0,139500 |
| T1 | ds-U16 | IVSP3-1 |  | 29,9769567 | 15,8012524 | 0,000115 |
| T2 | ds-U16 | IVSP3-1 |  | 30,4371395 | 16,200272 | 0,000111 |
| T3 | ds-U16 | IVSP3-1 |  | 27,6366101 | 16,1651763 | 0,000695 |
| T4 | ds-U16 | IVSP3-1 |  | 27,7003189 | 16,7928027 | 0,001020 |
| T5 | ds-U16 | IVSP3-1 |  | 28,2065626 | 16,3985105 | 0,000558 |
| T6 | ds-U16 | IVSP3-1 |  | 25,0240286 | 16,124396 | 0,003820 |
| T7 | ds-U16 | IVSP3-1 |  | 27,268341 | 16,3187396 | 0,000985 |
| T8 | ds-U16 | IVSP3-1 |  | 30,5376957 | 16,5495418 | 0,000132 |
| C1 | ds-GFP | IVSP4-1 | 1,8 | 19,4638796 | 16,7441326 | 0,997300 |
| C2 | ds-GFP | IVSP4-1 |  | 18,9951229 | 16,5891812 | 1,182000 |
| C3 | ds-GFP | IVSP4-1 |  | 17,986244 | 16,4614331 | 1,959000 |
| C4 | ds-GFP | IVSP4-1 |  | 18,6902417 | 16,5955505 | 1,420000 |
| T1 | ds-U16 | IVSP4-1 |  | 29,9786805 | 15,8012524 | 0,001080 |
| T2 | ds-U16 | IVSP4-1 |  | 31,4686942 | 16,200272 | 0,000593 |
| T3 | ds-U16 | IVSP4-1 |  | 29,2283364 | 16,1651763 | 0,002160 |
| T4 | ds-U16 | IVSP4-1 |  | 29,1676993 | 16,7928027 | 0,003440 |
| T5 | ds-U16 | IVSP4-1 |  | 27,0519115 | 16,3985105 | 0,009100 |
| T6 | ds-U16 | IVSP4-1 |  | 24,7903055 | 16,124396 | 0,028500 |
| T7 | ds-U16 | IVSP4-1 |  | 27,423395 | 16,3187396 | 0,006930 |
| T8 | ds-U16 | IVSP4-1 |  | 30,8250387 | 16,5495418 | 0,001100 |
| C1 | ds-GFP | IVp12-1 | 1,94 | 15,9322403 | 16,7441326 | 2,410000 |
| C2 | ds-GFP | IVp12-1 |  | 15,2291684 | 16,5891812 | 3,455000 |
| C3 | ds-GFP | IVp12-1 |  | 14,6310429 | 16,4614331 | 4,706000 |
| C4 | ds-GFP | IVp12-1 |  | 14,8752248 | 16,5955505 | 4,387000 |
| T1 | ds-U16 | IVp12-1 |  | 28,6898175 | 15,8012524 | 0,000270 |
| T2 | ds-U16 | IVp12-1 |  | 29,9650882 | 16,200272 | 0,000152 |
| T3 | ds-U16 | IVp12-1 |  | 23,0432462 | 16,1651763 | 0,014600 |
| T4 | ds-U16 | IVp12-1 |  | 20,9213441 | 16,7928027 | 0,091300 |
| T5 | ds-U16 | IVp12-1 |  | 21,2608285 | 16,3985105 | 0,055700 |
| T6 | ds-U16 | IVp12-1 |  | 19,7986522 | 16,124396 | 0,121700 |
| T7 | ds-U16 | IVp12-1 |  | 22,4264359 | 16,3187396 | 0,024400 |
| T8 | ds-U16 | IVp12-1 |  | 28,188384 | 16,5495418 | 0,000627 |
| C1 | ds-GFP | U16 | 2 | 21,6127967 | 16,7441326 | 0,028900 |
| C2 | ds-GFP | U16 |  | 20,9484002 | 16,5891812 | 0,041200 |
| C3 | ds-GFP | U16 |  | 20,5625079 | 16,4614331 | 0,049400 |
| C4 | ds-GFP | U16 |  | 20,6935446 | 16,5955505 | 0,049400 |
| T1 | ds-U16 | U16 |  | 28,9304436 | 15,8012524 | 0,000095 |
| T2 | ds-U16 | U16 |  | 29,4623165 | 16,200272 | 0,000087 |
| T3 | ds-U16 | U16 |  | 28,0400333 | 16,1651763 | 0,000226 |
| T4 | ds-U16 | U16 |  | 28,1874821 | 16,7928027 | 0,000314 |
| T5 | ds-U16 | U16 |  | 29,1689334 | 16,3985105 | 0,000121 |
| T6 | ds-U16 | U16 |  | 26,3378402 | 16,124396 | 0,000716 |
| T7 | ds-U16 | U16 |  | 27,7512227 | 16,3187396 | 0,000307 |
| T8 | ds-U16 | U16 |  | 27,4073475 | 16,5495418 | 0,000456 |
| C1 | ds-GFP | U22 | 1,92 | 20,9079969 | 16,7441326 | 0,110700 |
| C2 | ds-GFP | U22 |  | 20,2052881 | 16,5891812 | 0,157500 |
| C3 | ds-GFP | U22 |  | 20,0028511 | 16,4614331 | 0,164700 |
| C4 | ds-GFP | U22 |  | 20,0466639 | 16,5955505 | 0,175400 |
| T1 | ds-U16 | U22 |  | 29,2209132 | 15,8012524 | 0,000257 |
| T2 | ds-U16 | U22 |  | 29,5875823 | 16,200272 | 0,000265 |
| T3 | ds-U16 | U22 |  | 27,7676394 | 16,1651763 | 0,000849 |
| T4 | ds-U16 | U22 |  | 27,10156 | 16,7928027 | 0,002010 |
| T5 | ds-U16 | U22 |  | 27,9071105 | 16,3985105 | 0,000909 |
| T6 | ds-U16 | U22 |  | 25,2997209 | 16,124396 | 0,004130 |
| T7 | ds-U16 | U22 |  | 27,0585591 | 16,3187396 | 0,001500 |
| T8 | ds-U16 | U22 |  | 28,0668294 | 16,5495418 | 0,000908 |
| C1 | ds-GFP | U23 | 1,76 | 18,1154624 | 16,7441326 | 3,310000 |
| C2 | ds-GFP | U23 |  | 17,7277539 | 16,5891812 | 3,707000 |
| C3 | ds-GFP | U23 |  | 17,0135627 | 16,4614331 | 5,087000 |
| C4 | ds-GFP | U23 |  | 17,371101 | 16,5955505 | 4,555000 |
| T1 | ds-U16 | U23 |  | 30,1202562 | 15,8012524 | 0,001960 |
| T2 | ds-U16 | U23 |  | 30,5771966 | 16,200272 | 0,001990 |
| T3 | ds-U16 | U23 |  | 24,8188274 | 16,1651763 | 0,050400 |
| T4 | ds-U16 | U23 |  | 24,7661953 | 16,7928027 | 0,079700 |
| T5 | ds-U16 | U23 |  | 24,9040895 | 16,3985105 | 0,056300 |
| T6 | ds-U16 | U23 |  | 22,6574254 | 16,124396 | 0,166300 |
| T7 | ds-U16 | U23 |  | 24,9574358 | 16,3187396 | 0,051700 |
| T8 | ds-U16 | U23 |  | 29,4884303 | 16,5495418 | 0,004680 |

**Table F.** RNA quantification (Cf Fig 9A). Statistical analyses.

**DNA amplification of Hd29 segment in ds*GFP* and ds*U16*-injected wasps**

**Table G.** Genomic DNA amplification (Cf Fig 9B). Raw data.

| Biological replica ID | Sample | Target gene | Efficiency | Mean Ct Target gene | Mean Ct reference (*ELF-1*) | Relative amplification to *ELF-1* |
| --- | --- | --- | --- | --- | --- | --- |
| C1 | ds-GFP-calyx-R1 |  |  | 28,0774299 | 29,637748 | 4,457195 |
| C2 | ds-GFP-calyx-R2 |  |  | 28,0148388 | 29,5776069 | 4,460397 |
| C3 | ds-GFP-calyx-R3 |  |  | 28,5395861 | 30,2490804 | 4,970287 |
| T1 | ds-U16-calyx-R1 | *rpl (ribosomal protein)* | 1,95 | 28,1386785 | 29,1636183 | 3,094851 |
| T2 | ds-U16-calyx-R2 |  |  | 28,0871373 | 28,9485737 | 2,765618 |
| T3 | ds-U16-calyx-R3 |  |  | 28,5755791 | 29,9967644 | 4,084029 |
| C1 | ds-GFP-calyx-R1 |  |  | 23,3347142 | 29,637748 | 58,619111 |
| C2 | ds-GFP-calyx-R2 |  |  | 23,3295587 | 29,5776069 | 56,461112 |
| C3 | ds-GFP-calyx-R3 |  |  | 24,0019033 | 30,2490804 | 56,047514 |
| T1 | ds-U16-calyx-R1 | *Proviral left* | 2 | 28,4297181 | 29,1636183 | 1,240602 |
| T2 | ds-U16-calyx-R2 |  |  | 28,3701144 | 28,9485737 | 1,116294 |
| T3 | ds-U16-calyx-R3 |  |  | 27,4167033 | 29,9967644 | 4,423293 |
| C1 | ds-GFP-calyx-R1 |  |  | 23,4302903 | 29,637748 | 54,861535 |
| C2 | ds-GFP-calyx-R2 |  |  | 23,4571132 | 29,5776069 | 51,683474 |
| C3 | ds-GFP-calyx-R3 |  | 2 | 23,9850448 | 30,2490804 | 56,706295 |
| T1 | ds-U16-calyx-R1 | *Proviral right* |  | 26,9238901 | 29,1636183 | 3,523156 |
| T2 | ds-U16-calyx-R2 |  |  | 26,9317307 | 28,9485737 | 3,025348 |
| T3 | ds-U16-calyx-R3 |  |  | 26,2866893 | 29,9967644 | 9,680858 |
| C1 | ds-GFP-calyx-R1 |  |  | 20,750977 | 29,637748 | 351,416076 |
| C2 | ds-GFP-calyx-R2 |  |  | 20,7388801 | 29,5776069 | 340,111559 |
| C3 | ds-GFP-calyx-R3 |  |  | 21,1222133 | 30,2490804 | 412,505184 |
| T1 | ds-U16-calyx-R1 | *Episomal* | 2 | 32,0571892 | 29,1636183 | 0,100382 |
| T2 | ds-U16-calyx-R2 |  |  | 32,1715572 | 28,9485737 | 0,080063 |
| T3 | ds-U16-calyx-R3 |  |  | 28,6674631 | 29,9967644 | 1,858787 |
| C1 | ds-GFP-calyx-R1 |  |  | 20,4766357 | 29,637748 | 425,017271 |
| C2 | ds-GFP-calyx-R2 |  |  | 20,4936871 | 29,5776069 | 403,117660 |
| C3 | ds-GFP-calyx-R3 |  |  | 20,860789 | 30,2490804 | 494,454085 |
| T1 | ds-U16-calyx-R1 | *Proviral + Episomal* | 2 | 28,4503626 | 29,1636183 | 1,222976 |
| T2 | ds-U16-calyx-R2 |  |  | 28,5207201 | 28,9485737 | 1,005638 |
| T3 | ds-U16-calyx-R3 |  |  | 26,19514 | 29,9967644 | 10,315088 |

**Table H.** Genomic DNA amplification (Cf Fig 9B). Statistical analyses.
