# Supplementary material for: Identification of a viral gene essential for the genome replication of a domesticated endogenous virus in ichneumonid parasitoid wasps
Source: PLoS Pathog. 2024 Apr 25;20(4):e1011980. doi: 10.1371/journal.ppat.1011980 (PMC11075835; doi:10.1371/journal.ppat.1011980)
Supplement: S3 Dataset — The dataset includes: Table A. Raw sequence of U16 proteins. Table B. Position of the PriCT-domain in U16 proteins. Fig A. Detail of the predicted secondary structure of the PricT-2 domain in the H. didymator U16 protein. Fig B. Subcellular localization of U16 predicted by DeepLoc 2.0. (DOCX) [file ppat.1011980.s003.docx]

**S3 Dataset. Sequence analysis and alignment of the U16 gene from *H. didymator* to four other wasp species that harbor IVs.**

The dataset includes:

**Table A.** Raw sequence of U16 proteins.

**Table B.** Position of the PriCT-domain in U16 proteins.

**Fig A.** Detail of the predicted secondary structure of the PricT-2 domain in the *H. didymator* U16 protein.

**Fig B.** Subcellular localization of U16 predicted by DeepLoc 2.0.

**Table A.** Raw sequence of U16 proteins from *H. didymator* [1], *Campoletis sonorensis* [2], *Bathyplectes anurus* [3], *Lissonota* sp [4] and *Glypta fumiferanae* [5].

>Hd_IVSP_U16 [Hyposoter didymator]

MVKSIEDFVFQGLSTKDQPSSHLFPRKCMVTRANTNGFDALDLFAASYKPQIGQYKCFQEKNGCAPEQGYPYRIVVDLDSSDEHLLQSLLYEIGKLLEKLLCQANTSDFKVMICIMRKQQTGRFHVHLLNVVTNDLTTYKNFLIMLHEKVQAVDKGAGVNYFMVFGAIKAYKLNVNPTTPAADQCYLPWKLCCAEPNEIDLNNFVDLPGFTGGSCDEYLEDIYNYFKKHFEPFSCKTLFHALSLHRRYNPDFDVVLPSCQDSLVRCAKRRANEDSDSEGKKKVRTGKSDPINRAKESFFENVLFKLPRNYYEEYDSWIGIGKIIAYVKQNYGLHLFHKFSAQSRNKYDAEKVTATYEGLLETIKVNQGEGEDEPAIRTTSALRTLLLGSNSIIDQIEHKMFYKWKGDTHAIVGAVNCCIQQMSPMLTFPHPLNANYLFAIEFSRSDGAYRISHDTFIQSFGGQRATIDAGVERHEHYERILECFIYLAIKYYNDNHRFLRMYTAHGILDSTPFKLKNHLHAKNNAEDMRSSQYSFYRRAVVQKLNKLKKRWFLAQANGATTVSRKNNKRLWSKLYAEFEFHRALQSARKCGRYPQSVLVKAVTRSGPPRTAL

>Cs_IVSP_U16L [Campoletis sonorensis]

MVKCIGEFTFQGLSATDQPSSHLFPRKCMVCAPNTSGFDALDMFAASYKPQIGQLKCFQEKNGCAPEHGYPYRIVVDVDSNDENLLQSLLCEMGKLLEELLPQNKISDLKILICIMRKQQTGRFHIHLLNVVSNDLVTYKNFLIALHEKFQAVDRGAGVNYFMVFGAVKAYKQNVDPTTSLADQCYLPWKMCCTKINEIRLNNFINLPAFTGGSSDEYCNNIQTYFNKYSQRSSFVTLFHLLSLHRRYNPDFDVVLPPAQDSVVRCAKKRANDGSVSEGKKKVRTAKSDPINRAKESFFENVLFKLPKNYYEEYDSWIRIGKIIAYVAQGHGLHLFHKFSSQSREKYDAEKVTSTYEGLLEAMKVNQSVEDDKPVIRTTSALRSLLLGSNSIIDQIERKMFYKWKGDTHAIVGAVTSCTQQMSPMLTFPHPLNANYWFAIEFSRTDGAYKISHDTFIQSFGGQRATIDAGVERHEHYERILECFIYLAIKYYNDHYRFLRMYTAHGILDSTPFKLKHHLHAKNNVEDMRSSQHSFYRRTVVHKLNKLKKRWLLAQSNDATAVSKKKNRRLWKKLYTQFEFSRALESARKCGRYPQSVFDKAVPRSDASMCSR

>Ba_IVSP_U16L [Bathyplectes anurus]

MIKSIGGFTFQGCNEKDQPSSHLFPRKCMVCTPNASGYNALDLFAASYKPQIGKLKCFQEKNGCAPERGYPYRIVVDLDSNDENLLESLLCEIRKLLRELLPQAEASDFKLIICILRKQQTGRFHIHLLNVVSNDLITYKNFLMVLHEKIKAVDRGAGVNYFMVFGAIKAYKQKVDPTTPVADQCYLPWKMCSAKINEIKLNNFIDLPPFTGGSSDEYCNDIQNYFNKYFQRSSCGTLFHLLSLHRRYNPDFDVVLPLCQESIVRSSRKRTSDDSDSEGKKKIRTAKSDPINRAKESFFENVLFKLPKNYYEEYDSWIGIGKIIAFVKQTDGLNLFHKFSSQSRDKYDAERVATTYEGLLEAMKVNQSDKAAKPVIHMTSALRALLLGSNSIIDQIERKMFYKWKGDTHAIVGAVTCCIQQMSPMLTFPHPLNANYLFAIEFSRVDGAYRISHDTFIQSFGGQRATIDAGVERHEHYERILECFIYLAIKYFNDNHRFWRMYSAHSILDSTPFKLKQHLHAKNNVEDMRSSQHSFYRRAVVQKLNKLKKRWLLAQSNGATISSKKKNKRLWSKLYTQYEFQRALDSARKCGRYPQSVFDKAVPRSDASSCSR

>Lsp_IVSP_U16L [Lissonota sp. PSUC_FEM 10030012]

MVKRIDNFTFIPHKSDNTKPVSHLFPQKCRVTTAESDGEDALLAFVNGYKPQIGELKCFQEKNGYSTELGHPFRLVVDLDSSSELDLENVLSEIRNIVNQYLCQETNDINIIVCILRKAQSGRFHIHLLNLVCNNQESYKAFLNRLHLSIKNVDNKAGLNYFMSFGSIKTYRAESSSSIEPSHRSCYLPWKMLHATSTAQMRLDNFSDIPEFEDFENSDRYCRFVFNNFFRSHVPSSVANSLFGLLSLHRKYVDDFDHTLPIPADSAIVGSRKRKPKRLPSFHLPEEDDDPDDEETSGWINNKKKKRNKGRLEILRETYLENALFKLPSYYYDNYNGWLLVGFLIAKEWRDAGRFLYHKFSSLSKKYDAVTVDAKYTNLLGSLREGENPSLGTTHLAPLLLESNSILEDMEKKMFHCWKGPIYSIVSAVGVYVRHMSPMLIFPHPLNANYIYAIEYSSLEGIYRPTHDTFMQSFGGQRGTMEMSVERSEHYGKLLDCIIYLALKNYNDNHRFTVMCKAHETLDSTPSRLKVQLNSRITSEERRNTEMSFYRRLLVKKLNKLMKRWQMIKVTRCKNNKHLWAKLYSEFNYGRALVSAKRHDRYPQSVFEKAIALKDVPSRNRSQISCDREEYTGEY

>Gf_IVSP_U16L [Glypta fumiferanae]

MVKRIDNFIFIPHKSDNTKPFTHLFPQKCRVTTAKSDGQDALLAFVNTYKPQIGDLRCFQEKNGYSTELGHPFRLVVDLDSSSELDLENVLVEIRNIVNQYLCSKTNDINIIVCILKKAQSGRFHIHLLNLVCNNQESYKAFLNRLHLNIKNVDNKAGLNYFMSFGSVKTYRAERSSSIEPSHRSCYLPWKMLHATNTDQMRLNNFSNIPEFDDFENSDKYCRFVFNNFFRSHVPSCVANTLFGLLSLHRKYIDEFDHTLPIPAETGTEGFRKRKQKRLPSVDVPEEEDDGDDEETNGWVNKRKKKSKGRLEILRETYLENALFKLPSYYYDNYNGWSFVGFLIAREWRDAGRFLYHKFSSLSKKYNAETVDAKFTSLLAVLREGENLNQPSPALGTTHLAPLLLESNSILEDMEKKMFHSWKGPIYSIVSAVGVYVRHMSPMLIFPHPLNANYIYAIEYCSMEGIYRPTHDTFMQSFGGQRGTMEMSVERSEHYGKLLDCIIYLALKNYNDNHRFTVMCKAHETLDSTPSRLKVQLNSRITSEERRNTELSFYRRLIVKKLNKLMKRWGSIEIAKNRSNKYLWSKLYSEFDYGQALVSAKRHDRYSQSVFEKAIVIKDNSVSNRSEARCDQEYQVNGY

**Table B.** Position of the PriCT-domain in U16 proteins.

| **Parasitoid species** | **U16 size** | **PriCT-2 position (e-value)** |
| --- | --- | --- |
| *Hyposoter didymator* (Hd) | 612 aa | 303-365 (6.18e-03) |
| *Campoletis sonorensis* (Cs) | 612 aa | 303-365 (2.64e-04) |
| *Bathyplectes anurus* (Ba) | 612 aa | 303-365 (6.41e-04) |
| *Lissonota* sp. (Lsp) | 635 aa | 322-382 (9.91e-05) |
| *Glypta fumiferanae* (Gf) | 639 aa | 321-379 (1.98e-05) |

**Fig A. Detail of the secondary structure of the *H. didymator* U16 protein PricT-2 domain**

Prediction obtained using the Quick2D set on the MPI bioinformatic Toolkit software (<https://toolkit.tuebingen.mpg.de/tools/quick2d>) [6]. Vertical arrows indicate the start and the end of the predicted PriCT-2 domain in *H. didymator* U16 protein.

**
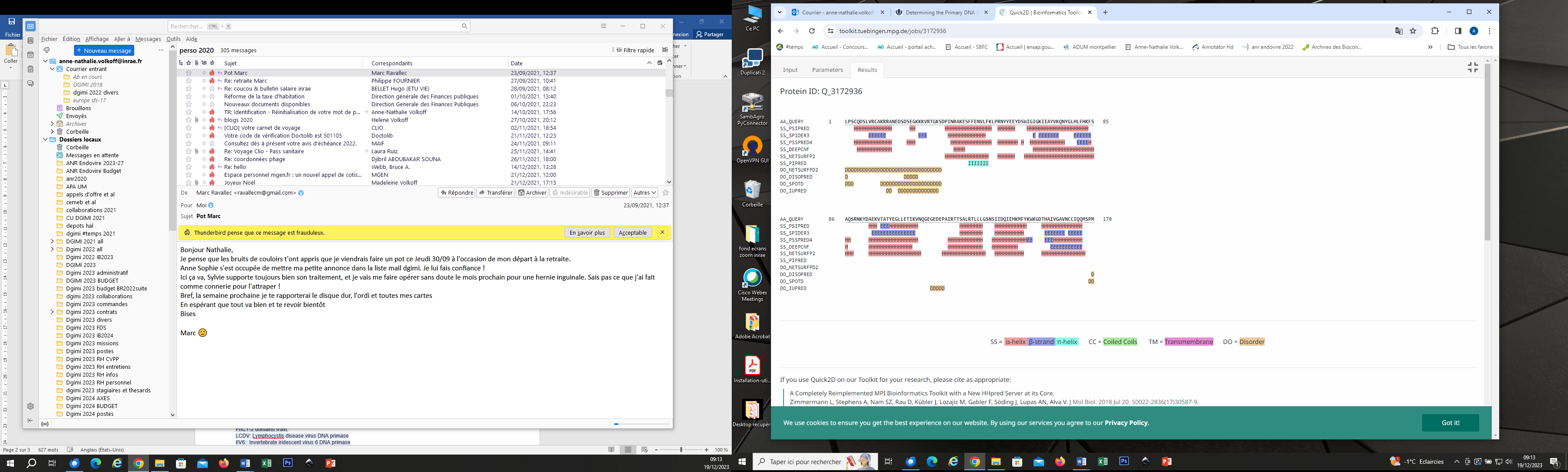

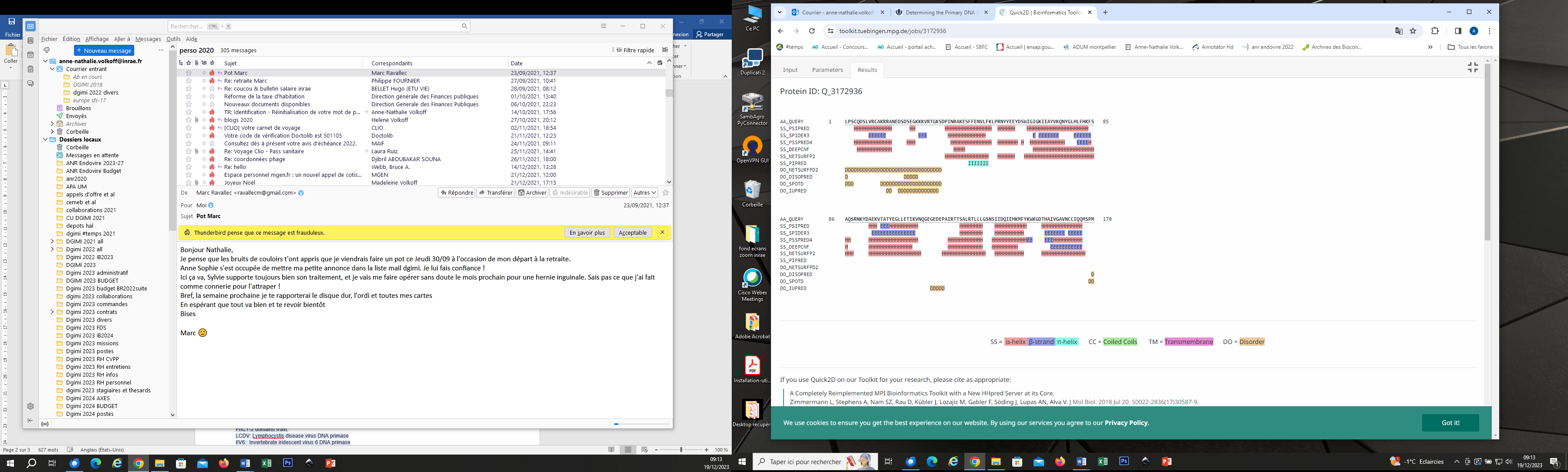
**

**Fig B. U16 subcellular localization predicted using DeepLoc 2.0 [7].**

DepLoc2.0 subcellular localization prediction (Probability): Nucleus (0.8123); Cytoplasm (0.1747); Extracellular (0.0426); Cell membrane (0.0400); Mitochondrion (0.0824); Plastid (0.0051); Endoplasmic reticulum (0.0303); Lysosome/Vacuole (0.0748); Golgi apparatus (0.0517); Peroxisome (0.0755)

Bellow: Logo-like plot of the positions in the query protein with higher importance for the prediction of nuclear localization.


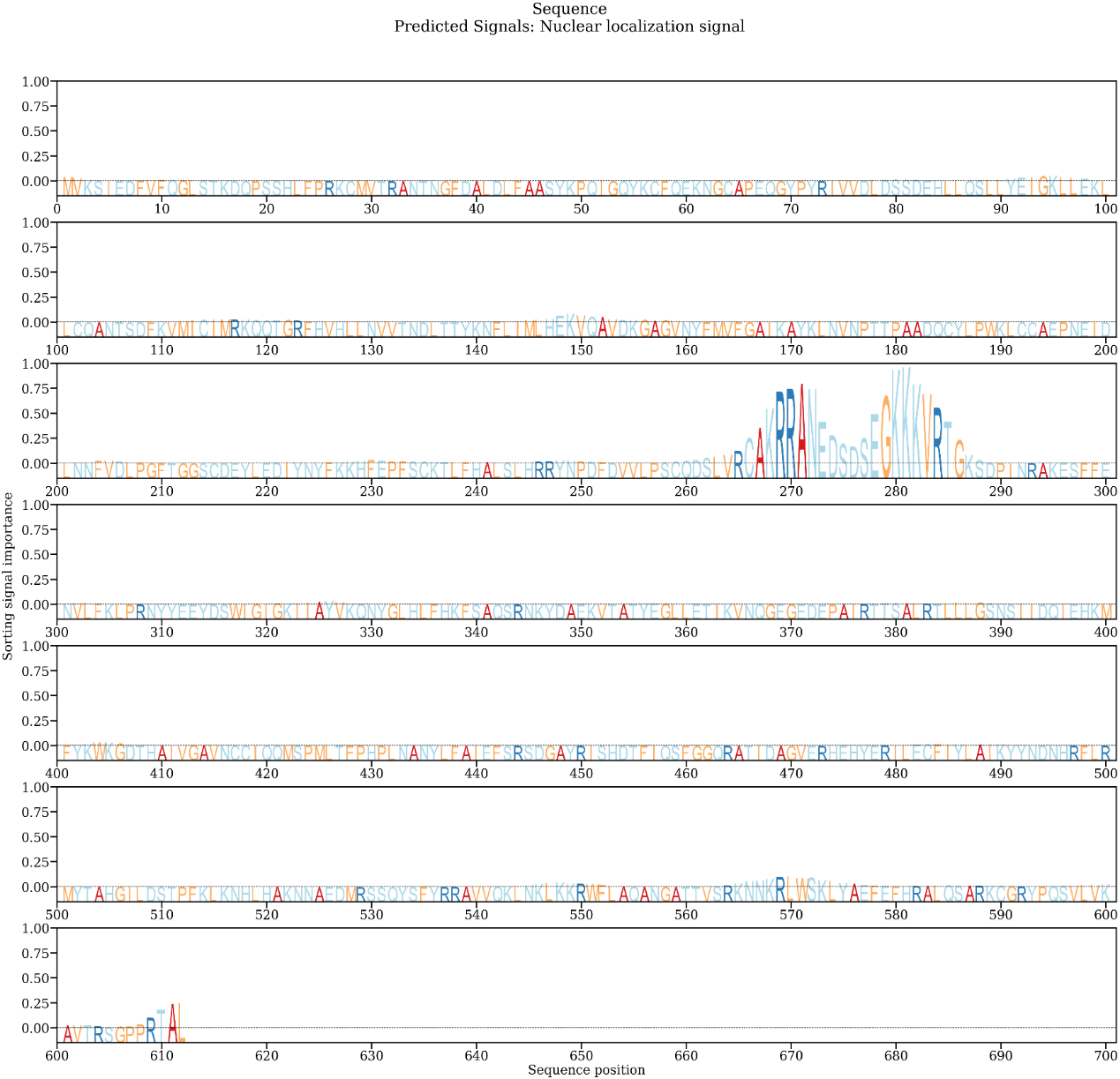


References

1. Volkoff AN, Jouan V, Urbach S, Samain S, Bergoin M, Wincker P, et al. Analysis of virion structural components reveals vestiges of the ancestral ichnovirus genome. PLoS Pathog. 2010 May 27;6(5):e1000923. doi: 10.1371/journal.ppat.1000923.
2. Legeai F, Santos BF, Robin S, Bretaudeau A, Dikow RB, Lemaitre C, et al. Genomic architecture of endogenous ichnoviruses reveals distinct evolutionary pathways leading to virus domestication in parasitic wasps. BMC Biol. 2020 Jul 24;18(1):89. doi: 10.1186/s12915-020-00822-3.
3. Robin S, Ravallec M, Frayssinet M, Whitfield J, Jouan V, Legeai F, et al. Evidence for an ichnovirus machinery in parasitoids of coleopteran larvae. Virus Res. 2019;263: 189–206. doi: 10.1016/j.virusres.2019.02.001.
4. Burke GR, Hines HM, Sharanowski BJ. The presence of ancient core genes reveals endogenization from diverse viral ancestors in parasitoid wasps. Genome Biol Evol. 2021 Jul 6;13(7):evab105. doi: 10.1093/gbe/evab105. PMID: 33988720.
5. Béliveau C, Cohen A, Stewart D, Periquet G, Djoumad A, Kuhn L, et al. Genomic and Proteomic Analyses Indicate that Banchine and Campoplegine Polydnaviruses Have Similar, if Not Identical, Viral Ancestors. J Virol. 2015 Sep;89(17):8909-21. doi: 10.1128/JVI.01001-15.
6. Gabler F, Nam SZ, Till S, Mirdita M, Steinegger M, Söding J, et al. Protein sequence analysis using the MPI Bioinformatics Toolkit. Curr Protoc Bioinformatics. 2020 Dec;72(1):e108. doi: 10.1002/cpbi.108.
7. Thumuluri V, Armenteros JJA, Johansen AR, Nielsen H, Winther O. DeepLoc 2.0: multi-label subcellular localization prediction using protein language models. Nucleic Acids Research. 2022. doi:10.1093/nar/gkac278.
