# Supplementary material for: Identification of a viral gene essential for the genome replication of a domesticated endogenous virus in ichneumonid parasitoid wasps
Source: PLoS Pathog. 2024 Apr 25;20(4):e1011980. doi: 10.1371/journal.ppat.1011980 (PMC11075835; doi:10.1371/journal.ppat.1011980)
Supplement: S1 Table — (DOCX) [file ppat.1011980.s004.docx]

**S1 Table. Read depth of HdIV loci on each scaffold of the *H. didymator* genome.** Start and end sites and size of each locus is indicated. Read depths for each locus were calculated based on the length of the locus. Data are given for calyx cell DNA samples that were collected from stage 1 and 3 pupae. The highest value of the DNA amplification for the locus (last column), was determined as the ratio between the Counts per Million (CPM, calculated for 10 bp intervals) value for stage 3 and the CPM value for stage 1 at the position displaying the highest ratio (summit).

| Scaffold | HdIV locus | Start | End | Size (bp) | Read depth of the locus at pupal stage 1 | Read depth of the locus at pupal stage 3 | CPM ratio between stage 3 / stage 1 at the curve summit |
| --- | --- | --- | --- | --- | --- | --- | --- |
| Scaffold-1 | Hd26 | 13523617 | 13528634 | 5,017 | 2.58 | 722.72 | 479.73 |
|  | Hd38 | 20666612 | 20670275 | 3,663 | 2.67 | 1,090.17 | 611.17 |
|  | Hd36 | 20670791 | 20674528 | 3,737 | 2.56 | 500.90 | 329.06 |
|  | Hd50 | 27247704 | 27253490 | 5,786 | 2.42 | 122.56 | 81.33 |
| Scaffold-2 | Hd49 | 2124324 | 2129588 | 5,264 | 2.35 | 505.47 | 299.33 |
|  | U37 | 2735040 | 2736878 | 1,838 | 2.66 | 88.02 | 44.89 |
|  | Hd46 | 2737536 | 2741644 | 4,108 | 2.44 | 187.66 | 109.56 |
|  | Hd43 | 2744162 | 2748320 | 4,158 | 2.53 | 364.55 | 240.15 |
|  | Hd22 | 5839467 | 5843644 | 4,177 | 2.48 | 152.09 | 86.53 |
|  | Hd44.1 | 16284645 | 16287653 | 3,008 | 2.57 | 217.54 | 113.63 |
|  | Hd44.2 | 16289605 | 16294435 | 4,830 | 2.62 | 122.27 | 70.63 |
| Scaffold-3 | Hd30 | 419458 | 423621 | 4,163 | 2.57 | 1,502.10 | 900.61 |
|  | Hd48 | 16689745 | 16699417 | 9,672 | 2.53 | 184.07 | 127.13 |
| Scaffold-4 | Hd19 | 6231095 | 6235534 | 4,439 | 2.57 | 427.63 | 264.17 |
|  | Hd41 | 12119882 | 12127834 | 7,952 | 2.34 | 618.46 | 637.52 |
|  | Hd45.2 | 12329917 | 12331967 | 2,050 | 0.98 | 83.08 | 200.54 |
|  | Hd45.1 | 15910208 | 15914421 | 4,213 | 1.38 | 186.30 | 242.37 |
| Scaffold-5 | Hd13 | 5876265 | 5882021 | 5,756 | 2.51 | 384.93 | 228.16 |
| Scaffold-6 | Hd40 | 2988772 | 2992265 | 3,493 | 2.42 | 53.57 | 31.25 |
|  | Hd9 | 7229774 | 7247665 | 17,891 | 2.49 | 148.38 | 147.42 |
|  | Hd1 | 11708977 | 11723747 | 14,770 | 6.54 | 283.78 | 121.04 |
|  | Hd31-34 | 11868292 | 11872410 | 4,118 | 2.74 | 945.47 | 566.08 |
| Scaffold-7 | Hd27 | 6697648 | 6701649 | 4,001 | 2.84 | 2,407.77 | 1,174.47 |
|  | Hd5 | 7084945 | 7098657 | 13,712 | 2.48 | 219.66 | 162.53 |
|  | Hd47 | 7900815 | 7905317 | 4,502 | 2.56 | 248.24 | 130.95 |
|  | IVSPER-5 | 9178274 | 9179903 | 1,629 | 2.73 | 21.54 | 10.06 |
|  | IVSPER-3 | 9252903 | 9278334 | 25,431 | 2.45 | 341.96 | 211.66 |
|  | IVSPER-4 | 13191258 | 13207069 | 15,811 | 2.49 | 61.44 | 38.22 |
|  | Hd7 | 13968918 | 13976983 | 8,065 | 2.49 | 156.74 | 111.94 |
|  | Hd2 | 14085672 | 14099608 | 13,936 | 2.64 | 373.33 | 252.40 |
|  | Hd6 | 14221056 | 14231516 | 10,460 | 3.43 | 473.28 | 261.40 |
|  | Hd2-like | 14388343 | 14389879 | 1,536 | 3.48 | 527.86 | 221.67 |
| Scaffold-8 | Hd20 | 1942407 | 1949270 | 6,863 | 2.41 | 189.21 | 162.14 |
|  | Hd8 | 6823745 | 6831100 | 7,355 | 2.43 | 404.10 | 278.69 |
|  | Hd4 | 7097009 | 7107334 | 10,325 | 2.22 | 779.57 | 596.09 |
|  | Hd17 | 9012106 | 9019835 | 7,729 | 2.35 | 342.21 | 303.59 |
|  | Hd18 | 9364794 | 9369489 | 4,695 | 2.47 | 770.86 | 413.25 |
| Scaffold-9 | Hd39 | 5744955 | 5749076 | 4,121 | 2.44 | 242.93 | 156.35 |
| Scaffold-10 | Hd23.2 | 3553203 | 3556564 | 3,361 | 2.81 | 90.14 | 43.62 |
|  | Hd23.1 | 3592527 | 3596983 | 4,456 | 2.46 | 229.23 | 165.30 |
|  | Hd25 | 9399172 | 9403345 | 4,173 | 2.53 | 496.34 | 277.27 |
| Scaffold-11 | Hd10 | 453966 | 460472 | 6,506 | 2.39 | 403.48 | 263.14 |
|  | Hd11 | 768148 | 777337 | 9,189 | 2.35 | 360.60 | 311.13 |
|  | Hd16 | 2262941 | 2270644 | 7,703 | 2.48 | 1,298.26 | 992.83 |
|  | Hd12 | 2280031 | 2285932 | 5,901 | 2.34 | 176.88 | 146.49 |
|  | Hd29 | 3146273 | 3150628 | 4,355 | 2.62 | 587.39 | 315.64 |
|  | IVSPER-2 | 3155228 | 3181838 | 26,610 | 2.48 | 153.40 | 88.37 |
|  | Hd24 | 3182748 | 3187444 | 4,696 | 2.59 | 195.72 | 109.30 |
|  | Hd33 | 3234245 | 3238079 | 3,834 | 2.55 | 243.63 | 135.16 |
|  | Hd15 | 3249051 | 3254037 | 4,986 | 2.59 | 363.46 | 208.51 |
|  | IVSPER-1 | 3255266 | 3269285 | 14,019 | 2.47 | 141.13 | 113.40 |
|  | Hd14 | 3759577 | 3764772 | 5,195 | 2.34 | 325.08 | 187.61 |
|  | Hd32 | 3811943 | 3819858 | 7,915 | 2.24 | 401.60 | 387.46 |
|  | Hd42 | 6043764 | 6046920 | 3,156 | 2.39 | 184.42 | 116.10 |
|  | Hd21 | 6076348 | 6080715 | 4,367 | 2.45 | 311.65 | 173.90 |
|  | Hd3 | 6685111 | 6695124 | 10,013 | 2.33 | 806.66 | 560.49 |
|  | Hd37 | 6786206 | 6789913 | 3,707 | 2.25 | 220.01 | 136.79 |
| Scaffold-12 | Hd28 | 308843 | 313456 | 4,613 | 2.46 | 40.10 | 36.75 |
|  | Hd35 | 2941876 | 2945585 | 3,709 | 2.85 | 1,062.73 | 550.62 |
