# Supplementary material for: Identification of a viral gene essential for the genome replication of a domesticated endogenous virus in ichneumonid parasitoid wasps
Source: PLoS Pathog. 2024 Apr 25;20(4):e1011980. doi: 10.1371/journal.ppat.1011980 (PMC11075835; doi:10.1371/journal.ppat.1011980)
Supplement: S2 Table — (DOCX) [file ppat.1011980.s005.docx]

**S2 Table. List of the regions enriched in reads (peaks) predicted in *H. didymator* genome scaffolds using MACS2 algorithm.** For each region, the start and end positions are provided, along with the outputs from MACS2 for Broad peaks: fold change, -log10(pvalue), and -log10(qvalue) representing the mean value across all positions in the peak region. Previously known HdIV loci are highlighted with orange cells in the fold enrichment column (fold_change). Newly identified proviral segments are indicated in orange (Hd52, Hd53, and Hd54), while putative IV replication genes are marked in red (U38 to U42). Yellow cells in the fold enrichment column represent regions with a fold enrichment score >2, manually verified either to match with wasp genes with a high BlastP e-value or lacking clear evidence of transcription in the calyx (based on data from [1]). Read depth per amplified region (i.e. based on the predicted peak length) at pupal stages 1 and 3 is provided for each region.

| **Scaffold** | **Region start** | **Region end** | **fold_ change** | **-log10 (pvalue)** | **-log10 (qvalue)** | **Proviral locus ID** | **Read depth Stage 1** | **Read depth Stage 3** |
| --- | --- | --- | --- | --- | --- | --- | --- | --- |
| Scaffold-1 | 2185034 | 2193009 | 1.30577 | 3.70654 | 2.11144 |  | 2.58348 | 2.51615 |
|  | 2199889 | 2207005 | 1.36662 | 4.93397 | 3.28069 |  | 2.49154 | 2.66734 |
|  | 4878369 | 4912934 | 1.50793 | 8.55091 | 6.77718 |  | 2.54969 | 3.00330 |
|  | 7405158 | 7410203 | 1.58462 | 12.6884 | 10.7928 |  | 4.68815 | 5.18185 |
|  | 8803575 | 8812486 | 1.33296 | 4.27343 | 2.65156 |  | 2.57173 | 2.56450 |
|  | 12133732 | 12139644 | 1.27407 | 3.13212 | 1.57026 |  | 2.44676 | 2.23627 |
|  | 12572196 | 12590199 | 1.43326 | 6.43451 | 4.72094 |  | 2.46786 | 2.81677 |
|  | 13517317 | 13534392 | 2.30108 | 42.959 | 39.2482 | Hd26 | 2.48248 | 216.53155 |
|  | 19978928 | 19998650 | 1.45273 | 7.0171 | 5.28952 |  | 2.45972 | 2.83180 |
|  | 20656417 | 20681709 | 2.51698 | 54.5749 | 49.9013 | Hd36+Hd38 | 2.33986 | 246.31034 |
|  | 26084623 | 26093108 | 1.28112 | 3.25212 | 1.68287 |  | 2.50353 | 2.23467 |
|  | 27241046 | 27259533 | 2.2404 | 40.68 | 36.7212 | Hd50 | 2.35002 | 41.85925 |
| Scaffold-2 | 18792 | 29130 | 1.33184 | 4.21783 | 2.59736 |  | 2.60915 | 2.56435 |
|  | 68360 | 91362 | 1.41024 | 6.02083 | 4.33047 |  | 2.48036 | 2.70199 |
|  | 2117165 | 2135110 | 2.47947 | 51.5554 | 47.715 | Hd49 | 2.28573 | 154.78586 |
|  | 2679438 | 2704618 | 2.25074 | 40.0429 | 37.7806 | HD004510; wasp gene | 2.69515 | 7.92222 |
|  | 2728207 | 2748509 | 2.69635 | 63.5668 | 57.6371 | U37+Hd46+Hd43 | 2.88115 | 184.27235 |
|  | 2749360 | 2758008 | 2.23186 | 41.2132 | 37.5621 | end of Hd43 | 2.34499 | 23.91050 |
|  | 5832207 | 5850065 | 2.27775 | 40.9832 | 37.6924 | Hd22 | 2.33612 | 40.25669 |
|  | 6321570 | 6353333 | 1.49165 | 8.14587 | 6.38629 |  | 2.46967 | 2.96521 |
|  | 7316702 | 7336349 | 1.4496 | 6.84535 | 5.11762 |  | 2.48382 | 2.87621 |
|  | 7492850 | 7498100 | 1.27437 | 3.14194 | 1.57987 |  | 2.70799 | 2.30229 |
|  | 11057085 | 11065377 | 1.30776 | 3.73625 | 2.13974 |  | 2.55456 | 2.39022 |
|  | 11131303 | 11137723 | 1.28625 | 3.34211 | 1.76742 |  | 2.57344 | 2.32575 |
|  | 11166223 | 11176483 | 1.30626 | 3.71814 | 2.12289 |  | 2.53346 | 2.34889 |
|  | 11329813 | 11347577 | 1.50434 | 8.23641 | 6.46368 |  | 2.46564 | 3.09661 |
|  | 11379525 | 11385495 | 1.30445 | 3.67155 | 2.07796 |  | 2.51395 | 2.40498 |
|  | 13950273 | 13958021 | 1.29137 | 3.43858 | 1.85882 |  | 2.49524 | 2.32478 |
|  | 15136789 | 15150998 | 1.33498 | 4.29375 | 2.67078 |  | 2.48062 | 2.44187 |
|  | 15997706 | 16019240 | 1.88726 | 22.6199 | 20.5569 |  | 2.50917 | 4.57689 |
|  | 16132374 | 16144819 | 1.31739 | 3.94935 | 2.34282 |  | 2.55656 | 2.39533 |
|  | 16165725 | 16188844 | 1.94713 | 25.9989 | 23.9174 |  | 2.47814 | 5.24985 |
|  | 16278981 | 16302690 | 2.40503 | 49.3163 | 44.973 | Hd44.2+Hd44.1 | 2.45453 | 59.54165 |
|  | 17979905 | 18002447 | 1.40061 | 5.69328 | 4.00845 |  | 2.46717 | 2.69263 |
|  | 18356992 | 18379514 | 1.34164 | 4.4163 | 2.78695 |  | 2.45448 | 2.46584 |
|  | 18971354 | 18982075 | 1.30241 | 3.65755 | 2.0663 |  | 2.50532 | 2.35332 |
|  | 20387743 | 20408811 | 1.42826 | 6.363 | 4.65514 |  | 2.46175 | 2.74624 |
| Scaffold-3 | 410434 | 429813 | 2.58828 | 57.8437 | 53.2985 | Hd30 | 2.50996 | 335.88288 |
|  | 5880228 | 5897516 | 1.4245 | 6.33813 | 4.63442 |  | 2.47336 | 2.76064 |
|  | 6566362 | 6596353 | 1.47713 | 7.63789 | 5.88919 |  | 2.45635 | 2.92710 |
|  | 16679121 | 16706535 | 2.44145 | 50.7997 | 46.4311 | Hd48 | 2.42044 | 70.27019 |
|  | 17531347 | 17537369 | 1.43035 | 6.5188 | 4.81229 |  | 2.41320 | 2.86504 |
|  | 17537986 | 17545769 | 1.5215 | 8.92728 | 7.14511 |  | 2.30850 | 3.01088 |
|  | 20718102 | 20743617 | 1.56547 | 10.3021 | 8.48438 |  | 2.55182 | 3.44083 |
|  | 20748107 | 20771475 | 1.37278 | 5.11012 | 3.45078 |  | 2.57603 | 2.69848 |
|  | 20778136 | 20799849 | 1.53467 | 9.17409 | 7.37956 |  | 2.43699 | 3.21297 |
|  | 20801207 | 20808206 | 1.34609 | 4.52458 | 2.89034 |  | 2.63778 | 2.68429 |
| Scaffold-4 | 1353008 | 1363447 | 1.32579 | 4.11471 | 2.49975 |  | 2.41712 | 2.44771 |
|  | 3340947 | 3346697 | 1.30287 | 3.65084 | 2.05904 |  | 2.46821 | 2.39290 |
|  | 3371424 | 3388050 | 1.44312 | 6.84294 | 5.12483 |  | 2.49171 | 2.84679 |
|  | 4949375 | 4957931 | 1.40664 | 7.42867 | 5.67684 |  | 6.26680 | 4.76251 |
|  | 6222563 | 6228864 | 1.85906 | 21.9349 | 19.9045 |  | 2.43079 | 4.47319 |
|  | 6229498 | 6243248 | 2.32991 | 44.5738 | 40.6774 | Hd19 | 2.46537 | 142.62221 |
|  | 7231517 | 7238830 | 1.28354 | 3.29882 | 1.72681 |  | 2.53561 | 2.31123 |
|  | 12113372 | 12135096 | 2.51458 | 54.1878 | 49.3013 | Hd41 | 2.29180 | 232.72004 |
|  | 12321418 | 12340715 | 2.21551 | 39.1799 | 36.4031 | Hd45.2 | 2.13451 | 17.84939 |
|  | 15863989 | 15893575 | 1.34821 | 4.56569 | 2.92975 |  | 2.48967 | 2.51641 |
| Scaffold-5 | 3413526 | 3426939 | 1.44633 | 6.76791 | 5.04325 |  | 2.65101 | 2.88868 |
|  | 5869105 | 5889886 | 2.35262 | 46.2398 | 42.429 | Hd13 | 2.39194 | 112.33644 |
|  | 8214890 | 8226701 | 1.39782 | 5.60941 | 3.92736 |  | 2.45627 | 2.71007 |
|  | 8267888 | 8282165 | 1.52526 | 9.06983 | 7.27972 |  | 2.57871 | 3.16742 |
|  | 8316114 | 8369985 | 2.03216 | 28.138 | 25.9733 | HD011867-RA; NS | 2.51092 | 5.50794 |
|  | 8406028 | 8415752 | 1.2823 | 3.2799 | 1.70875 |  | 2.58454 | 2.28908 |
|  | 10188143 | 10197562 | 1.34719 | 4.54658 | 2.91179 |  | 2.54411 | 2.52744 |
|  | 10202683 | 10212864 | 1.49935 | 8.36667 | 6.6016 |  | 2.59388 | 3.10511 |
|  | 10214157 | 10225952 | 1.53164 | 9.25069 | 7.45851 |  | 2.64173 | 3.12859 |
|  | 13410644 | 13416814 | 1.30076 | 3.60668 | 2.01652 |  | 2.72956 | 2.56398 |
|  | 13480696 | 13486832 | 1.31378 | 3.93369 | 2.33289 |  | 2.64234 | 2.52439 |
|  | 13487863 | 13500191 | 1.32102 | 4.00285 | 2.39306 |  | 2.58431 | 2.60833 |
|  | 13501172 | 13510846 | 1.30465 | 3.78028 | 2.18331 |  | 3.45201 | 2.69846 |
|  | 13546596 | 13590940 | 1.49008 | 8.15606 | 6.39882 |  | 2.58973 | 3.10653 |
|  | 14084197 | 14089339 | 1.29221 | 4.52918 | 2.89475 |  | 6.87373 | 3.96149 |
|  | 15238461 | 15245303 | 1.81847 | 26.4145 | 24.2612 |  | 24.00287 | 16.95620 |
| Scaffold-6 | 2983699 | 2997266 | 1.89543 | 26.5736 | 23.5162 | Hd40 | 2.35038 | 15.99159 |
|  | 4744357 | 4750197 | 1.29498 | 3.49919 | 1.91568 |  | 2.54473 | 2.36688 |
|  | 7223110 | 7254609 | 2.50522 | 54.2614 | 49.2716 | Hd9 | 2.51361 | 86.95529 |
|  | 7337396 | 7345888 | 1.30358 | 3.66682 | 2.07439 |  | 2.50936 | 2.38654 |
|  | 9075600 | 9095671 | 2.18452 | 37.4216 | 35.1586 | U42* | 2.35536 | 7.99408 |
|  | 10196676 | 10202399 | 1.27002 | 3.06066 | 1.50293 |  | 2.56372 | 2.27515 |
|  | 11702950 | 11728752 | 2.19437 | 40.9325 | 38.6041 | Hd1 | 4.89306 | 168.16298 |
|  | 11862430 | 11878832 | 2.27232 | 41.6765 | 38.427 | Hd31-34 | 2.47574 | 241.92469 |
|  | 13037790 | 13085971 | 1.56631 | 10.4816 | 8.66316 |  | 2.50175 | 3.26306 |
|  | 13115690 | 13141219 | 1.40529 | 5.80949 | 4.12123 |  | 2.43602 | 2.70856 |
| Scaffold-7 | 72259 | 86779 | 1.35113 | 4.63063 | 2.99202 |  | 2.57888 | 2.61565 |
|  | 1343578 | 1358647 | 2.33247 | 43.6809 | 41.333 | HD007422-RA; wasp gene | 2.44352 | 10.25415 |
|  | 2628028 | 2642233 | 1.36985 | 5.05376 | 3.39688 |  | 2.53525 | 2.57577 |
|  | 4758477 | 4776797 | 2.45133 | 50.712 | 47.0742 | U38* | 2.29697 | 14.75900 |
|  | 6688177 | 6708677 | 2.4625 | 51.1607 | 47.2331 | Hd27 | 2.38091 | 479.38497 |
|  | 6765613 | 6783734 | 2.12157 | 34.3515 | 31.0638 | Hd52* | 2.34803 | 35.37937 |
|  | 7078958 | 7104504 | 2.63396 | 60.3303 | 54.8117 | Hd5 | 2.47680 | 124.37948 |
|  | 7409690 | 7419723 | 1.31928 | 3.98599 | 2.3773 |  | 2.52907 | 2.38674 |
|  | 7604621 | 7619065 | 1.57907 | 10.7192 | 8.89122 |  | 2.53997 | 3.31051 |
|  | 7894307 | 7912018 | 2.25761 | 41.3395 | 37.6478 | Hd47 | 2.37431 | 67.32005 |
|  | 8975583 | 8984746 | 1.34568 | 4.46871 | 2.83467 |  | 2.51324 | 2.55893 |
|  | 9168108 | 9191030 | 2.2379 | 39.766 | 37.3937 | IVSPER-5 | 2.36462 | 9.17331 |
|  | 9243166 | 9287918 | 2.71837 | 64.6511 | 58.1256 | IVSPER-3 | 2.42279 | 220.53817 |
|  | 11101424 | 11116888 | 2.03294 | 29.1669 | 27.0295 | wasp genes | 2.41299 | 5.87039 |
|  | 11738062 | 11764025 | 2.42836 | 50.742 | 46.3375 | U39* | 2.52859 | 33.43549 |
|  | 13161847 | 13169652 | 1.69113 | 15.8761 | 13.9444 |  | 3.09589 | 4.54898 |
|  | 13176550 | 13216107 | 2.44184 | 51.3316 | 46.4458 | IVSPER-4 | 2.58547 | 33.09181 |
|  | 13523608 | 13533171 | 1.27964 | 3.23261 | 1.66455 |  | 2.49764 | 2.26415 |
|  | 13965551 | 13978603 | 2.31686 | 45.5361 | 41.2032 | Hd7 | 2.48280 | 98.00431 |
|  | 14079432 | 14106161 | 2.55001 | 56.86 | 52.2597 | Hd2 | 2.49291 | 204.70211 |
|  | 14218171 | 14238167 | 2.45043 | 52.9257 | 50.4607 | Hd6 | 2.96900 | 253.82465 |
|  | 14390907 | 14403768 | 1.3491 | 6.04922 | 4.3662 |  | 6.22749 | 4.44348 |
| Scaffold-8 | 1934960 | 1956552 | 2.41156 | 48.8265 | 44.5676 | Hd20 | 2.27607 | 64.64881 |
|  | 2120175 | 2125496 | 1.25947 | 3.61615 | 2.02582 |  | 5.67938 | 3.58389 |
|  | 2624935 | 2642677 | 1.50285 | 8.30212 | 6.53228 |  | 2.41870 | 3.04463 |
|  | 2752511 | 2779646 | 1.64183 | 12.9032 | 11.0282 |  | 2.51562 | 3.53764 |
|  | 2829273 | 2854191 | 1.61092 | 11.9008 | 10.0495 |  | 2.49720 | 3.51544 |
|  | 2866568 | 2873809 | 1.29639 | 3.55483 | 1.96991 |  | 2.58140 | 2.31188 |
|  | 2889896 | 2895041 | 1.29418 | 3.50038 | 1.91794 |  | 2.67695 | 2.32431 |
|  | 2999894 | 3041381 | 1.51888 | 9.16234 | 7.38084 |  | 2.60160 | 3.15207 |
|  | 6818165 | 6837954 | 2.44018 | 50.6151 | 46.2711 | Hd8 | 2.30306 | 155.50709 |
|  | 7090110 | 7114242 | 2.57496 | 57.1066 | 52.0791 | Hd4 | 2.22583 | 339.84043 |
|  | 9006318 | 9026009 | 2.4553 | 51.0048 | 46.2886 | Hd17 | 2.23539 | 138.27328 |
|  | 9208814 | 9228042 | 2.38224 | 47.3174 | 43.224 | Hd54* | 2.25956 | 103.73108 |
|  | 9360612 | 9376778 | 2.41303 | 48.3089 | 44.4818 | Hd18 | 2.30735 | 229.25203 |
|  | 9385423 | 9391696 | 1.2991 | 4.55575 | 2.91902 |  | 5.86896 | 3.92181 |
|  | 12137881 | 12161480 | 1.5697 | 10.7015 | 8.88409 |  | 2.72343 | 3.54060 |
|  | 12406684 | 12413050 | 1.2948 | 3.50057 | 1.91713 |  | 2.55505 | 2.42953 |
| Scaffold-9 | 827344 | 833100 | 1.57476 | 13.4423 | 11.5391 |  | 5.87985 | 5.93141 |
|  | 2650085 | 2675949 | 2.11345 | 32.8171 | 30.6138 | HD008455.1-RA; wasp gene | 2.66719 | 6.92901 |
|  | 2712629 | 2739344 | 1.94686 | 24.537 | 22.4291 |  | 2.59436 | 5.12372 |
|  | 5534710 | 5545067 | 1.31004 | 3.79755 | 2.19801 |  | 2.60060 | 2.38419 |
|  | 5714059 | 5757032 | 2.51446 | 53.5439 | 49.7109 | Hd39 + U40* | 2.35703 | 36.86513 |
|  | 7040265 | 7060018 | 2.25763 | 41.3915 | 38.4657 | U41* | 2.24979 | 10.80453 |
|  | 11866173 | 11875022 | 1.32948 | 4.18757 | 2.5694 |  | 2.56884 | 2.61242 |
|  | 12095319 | 12114763 | 1.34337 | 4.4778 | 2.84717 |  | 2.43676 | 2.51705 |
| Scaffold-10 | 155419 | 161336 | 1.3272 | 5.40558 | 3.72771 |  | 8.43990 | 4.78626 |
|  | 2708912 | 2743748 | 1.65995 | 13.608 | 11.7187 |  | 2.60542 | 3.86020 |
|  | 3545352 | 3561495 | 2.43023 | 49.7283 | 46.4326 | Hd23.2 | 2.56837 | 26.83644 |
|  | 3587203 | 3605012 | 2.32902 | 44.7235 | 41.1402 | Hd23.1 | 2.37063 | 63.02112 |
|  | 9393559 | 9412007 | 2.27288 | 41.6891 | 38.1961 | Hd25 | 2.35742 | 117.38364 |
| Scaffold-11 | 447712 | 465323 | 2.45916 | 51.0898 | 46.6842 | Hd10 | 2.33748 | 153.95716 |
|  | 764311 | 783916 | 2.52704 | 55.0936 | 49.7688 | Hd11 | 3.83837 | 174.87599 |
|  | 2257264 | 2292899 | 2.59568 | 58.1736 | 53.3086 | Hd12+Hd16 | 2.32485 | 317.70367 |
|  | 3139131 | 3193136 | 2.75498 | 66.4367 | 59.8622 | Hd29+IVSPER-2+Hd24 | 2.44589 | 152.67090 |
|  | 3229681 | 3277203 | 2.71544 | 64.1522 | 58.5465 | Hd33+Hd15+IVSPER-1 | 2.44887 | 114.56153 |
|  | 3753870 | 3770377 | 2.42835 | 49.2794 | 45.0577 | Hd14 | 2.24606 | 107.12005 |
|  | 3807216 | 3823379 | 2.49647 | 53.8046 | 48.5363 | Hd32 | 2.23563 | 200.34486 |
|  | 5248145 | 5262048 | 1.32095 | 4.00188 | 2.39277 |  | 2.45508 | 2.40340 |
|  | 6039490 | 6052307 | 2.00977 | 30.1143 | 27.0419 | Hd42 | 2.21113 | 48.45070 |
|  | 6067122 | 6086904 | 2.39727 | 47.0528 | 43.5071 | Hd21 | 2.30989 | 75.03783 |
|  | 6177710 | 6199505 | 2.27748 | 42.7237 | 39.2882 | Hd53* | 2.45600 | 49.10217 |
|  | 6678585 | 6701155 | 2.65379 | 60.9311 | 55.0985 | Hd3 | 2.26510 | 366.64188 |
|  | 6779687 | 6795693 | 2.22328 | 38.872 | 35.3679 | Hd37 | 2.11716 | 54.97750 |
| Scaffold-12 | 305632 | 316324 | 2.04762 | 33.6323 | 30.1057 | Hd28 | 2.42653 | 18.95388 |
|  | 695145 | 700147 | 1.62976 | 16.444 | 14.4658 |  | 13.74352 | 8.29064 |
|  | 2935430 | 2954029 | 2.22965 | 40.5317 | 37.1927 | Hd35 | 2.35286 | 219.02554 |
|  | 6209819 | 6224397 | 1.4202 | 6.15307 | 4.45131 |  | 2.45865 | 2.80320 |
|  | 6616838 | 6630851 | 1.30942 | 3.76587 | 2.16767 |  | 2.49825 | 2.44033 |
|  | 6648068 | 6663456 | 1.32451 | 4.38551 | 2.76969 |  | 3.46988 | 3.21925 |
| Scaffold-18 | 8228 | 19003 | 1.69307 | 19.4763 | 17.4447 |  | 14.03120 | 10.29598 |
| Scaffold-22 | 59113 | 64470 | 1.56892 | 13.9793 | 12.0636 |  | 9.42636 | 6.96808 |
| Scaffold-31 | 17924 | 23081 | 1.84802 | 27.8033 | 25.615 |  | 28.02908 | 20.04656 |
| Scaffold-64 | 29466 | 36283 | 1.36214 | 6.1839 | 4.47686 |  | 6.55109 | 4.55819 |
| Scaffold-169 | 14781 | 22335 | 1.74743 | 22.1517 | 20.0718 |  | 14.82566 | 10.82847 |
| Scaffold-186 | 2721 | 9406 | 1.86875 | 28.7223 | 26.5157 |  | 31.36077 | 21.46417 |
| Scaffold-190 | 8180 | 16793 | 1.51628 | 11.9248 | 10.058 |  | 11.50017 | 7.12331 |
| Scaffold-265 | 6797 | 12331 | 1.84722 | 27.7948 | 25.6078 |  | 29.13768 | 21.02706 |
| Scaffold-277 | 2393 | 9510 | 1.69634 | 20.0331 | 17.9953 |  | 17.19146 | 12.01046 |
| Scaffold-426 | 946 | 7308 | 1.56918 | 13.6135 | 11.7034 |  | 8.40499 | 7.10557 |
| Scaffold-435 | 863 | 7643 | 1.45048 | 8.9478 | 7.14554 |  | 7.16503 | 5.26290 |
| Scaffold-493 | 174 | 7219 | 1.51918 | 12.6797 | 10.8104 |  | 14.60250 | 7.84412 |

Reference

1.Lorenzi A, Ravallec M, Eychenne M, Jouan V, Robin S, Darboux I, et al. RNA interference identifies domesticated viral genes involved in assembly and trafficking of virus-derived particles in ichneumonid wasps. PLoS Pathog. 2019 Dec 13;15(12):e1008210. doi: 10.1371/journal.ppat.1008210.
