# Supplementary material for: Identification of a viral gene essential for the genome replication of a domesticated endogenous virus in ichneumonid parasitoid wasps
Source: PLoS Pathog. 2024 Apr 25;20(4):e1011980. doi: 10.1371/journal.ppat.1011980 (PMC11075835; doi:10.1371/journal.ppat.1011980)
Supplement: S3 Table — (DOCX) [file ppat.1011980.s006.docx]

**S3 Table. Read depth of HdIV amplified regions in calyx cell DNA from ds*GFP*- and ds*U16*-injected female pupae.** Values correspond to read depth per proviral locus or per MACS2 predicted amplified region (peak) calculated on the total length of the selected region in ds*U16*- and ds*GFP*-injected females. Read depth per locus and per amplified region in non-treated females at stage 1 and stage 3 are also reminded (columns pupal stage 1 & pupal stage 3). For dsRNA-injected samples, values are given for each of the triplicates, and the mean read depth per region and per treatment is indicated. Loci newly identified in the present work are marked by asterisks.

|  |  | **Locus amplification (read depth per locus)** | | | | | | | | | | **MACS2 predicted region amplification (read depth per amplified region)** | | | | | | | | | |
| --- | --- | --- | --- | --- | --- | --- | --- | --- | --- | --- | --- | --- | --- | --- | --- | --- | --- | --- | --- | --- | --- |
| **Scaffold** | **HdIV locus** | ***Pupal stage 1*** | ***Pupal stage 3*** | **locus GFP_2** | **locus GFP_3** | **locus GFP_4** | **locus U16_2** | **locus U16_3** | **locus U16_4** | **GFP mean** | **U16 mean** | ***Pupal stage 1*** | ***Pupal stage 3*** | **peak GFP_2** | **peak GFP_3** | **peak GFP_4** | **peak U16_2** | **peak U16_3** | **peak U16_4** | **GFP mean** | **U16 mean** |
| Scaffold-1 | Hd26 | *2.6* | *722.7* | 960.9 | 772.5 | 648.3 | 29.9 | 2.6 | 2.9 | **793.9** | **11.8** | *2.5* | *216.5* | 284.8 | 228.8 | 192.6 | 10.6 | 2.5 | 2.6 | **235.4** | **5.2** |
|  | Hd38 | *2.7* | *1,090.2* | 1,319.2 | 1,005.0 | 874.3 | 33.9 | 2.7 | 3.1 | **1,066.2** | **13.3** | *2.3* | *246.3* | 277.0 | 210.5 | 185.2 | 9.7 | 2.4 | 2.6 | **224.2** | **4.9** |
|  | Hd36 | *2.6* | *500.9* | 531.3 | 410.0 | 361.2 | 16.4 | 2.4 | 2.8 | **434.2** | **7.2** |  |  |  |  |  |  |  |  |  |  |
|  | Hd50 | *2.4* | *122.6* | 110.2 | 95.5 | 82.4 | 6.1 | 2.5 | 2.8 | **96.0** | **3.8** | *2.4* | *41.9* | 36.4 | 31.6 | 27.7 | 3.7 | 2.5 | 2.5 | **31.9** | **2.9** |
| Scaffold-2 | Hd49 | *2.4* | *505.5* | 581.7 | 505.3 | 426.1 | 18.5 | 2.7 | 3.0 | **504.4** | **8.1** | *2.3* | *154.8* | 173.5 | 150.4 | 127.5 | 7.4 | 2.4 | 2.4 | **150.4** | **4.1** |
|  | U37 | *2.7* | *88.0* | 39.0 | 26.3 | 32.2 | 7.4 | 2.8 | 3.2 | **32.5** | **4.5** | *2.6* | *132.2* | 139.9 | 98.0 | 92.9 | 10.7 | 3.1 | 3.5 | **110.3** | **5.8** |
|  | Hd46 | *2.4* | *187.7* | 129.7 | 90.4 | 89.7 | 10.7 | 2.7 | 3.3 | **103.3** | **5.6** |  |  |  |  |  |  |  |  |  |  |
|  | Hd43 | *2.5* | *364.5* | 390.1 | 278.3 | 240.0 | 14.3 | 2.8 | 3.0 | **302.8** | **6.7** |  |  |  |  |  |  |  |  |  |  |
|  | Hd22 | *2.5* | *152.1* | 211.1 | 179.8 | 152.5 | 7.5 | 2.6 | 2.8 | **181.1** | **4.3** | *2.3* | *40.3* | 51.7 | 44.0 | 38.0 | 3.9 | 2.5 | 2.5 | **44.6** | **3.0** |
|  | Hd44.1 | *2.6* | *217.5* | 226.9 | 162.8 | 155.0 | 7.5 | 2.6 | 2.8 | **181.6** | **4.3** | *2.5* | *59.5* | 57.8 | 43.7 | 40.9 | 4.0 | 2.4 | 2.6 | **47.5** | **3.0** |
|  | Hd44.2 | *2.6* | *122.3* | 122.6 | 100.3 | 88.8 | 6.0 | 2.6 | 2.8 | **103.9** | **3.8** |  |  |  |  |  |  |  |  |  |  |
| Scaffold-3 | Hd30 | *2.6* | *1,502.1* | 1,607.5 | 1,238.9 | 1,052.7 | 43.7 | 2.7 | 2.9 | **1,299.7** | **16.4** | *2.5* | *335.9* | 351.3 | 270.1 | 230.7 | 12.0 | 2.6 | 2.7 | **284.0** | **5.8** |
|  | Hd48 | *2.5* | *184.1* | 146.6 | 129.9 | 116.0 | 7.4 | 2.5 | 2.8 | **130.8** | **4.3** | *2.4* | *70.3* | 54.6 | 47.9 | 43.3 | 4.5 | 2.5 | 2.7 | **48.6** | **3.2** |
| Scaffold-4 | Hd19 | *2.6* | *427.6* | 387.2 | 336.5 | 281.2 | 11.4 | 2.8 | 3.4 | **335.0** | **5.8** | *2.5* | *142.6* | 127.5 | 110.5 | 93.1 | 5.7 | 2.7 | 2.9 | **110.4** | **3.7** |
|  | Hd41 | *2.3* | *618.5* | 521.6 | 402.7 | 353.3 | 17.2 | 2.4 | 2.7 | **425.9** | **7.4** | *2.3* | *232.7* | 193.7 | 149.5 | 131.8 | 8.1 | 2.3 | 2.3 | **158.3** | **4.2** |
|  | Hd45.2 | *1.0* | *83.1* | 84.8 | 64.4 | 68.7 | 2.8 | 1.3 | 1.4 | **72.7** | **1.8** | *2.1* | *17.8* | 15.0 | 11.5 | 12.5 | 2.8 | 2.3 | 2.4 | **13.0** | **2.5** |
|  | Hd45.1 | *1.4* | *186.3* | 172.4 | 127.7 | 131.4 | 3.4 | 1.4 | 1.5 | **143.8** | **2.1** | *ND* | *ND* |  |  |  |  |  |  |  |  |
| Scaffold-5 | Hd13 | *2.5* | *384.9* | 365.0 | 327.6 | 265.8 | 13.7 | 2.5 | 3.0 | **319.4** | **6.4** | *2.4* | *112.3* | 104.1 | 93.0 | 76.2 | 5.8 | 2.4 | 2.6 | **91.1** | **3.6** |
| Scaffold-6 | Hd40 | *2.4* | *53.6* | 53.0 | 40.2 | 37.9 | 3.9 | 2.9 | 2.9 | **43.7** | **3.2** | *2.4* | *16.0* | 15.0 | 11.7 | 11.2 | 2.9 | 2.5 | 2.6 | **12.7** | **2.6** |
|  | Hd9 | *2.5* | *148.4* | 112.0 | 119.3 | 84.3 | 7.7 | 2.5 | 3.0 | **105.2** | **4.4** | *2.5* | *87.0* | 65.2 | 69.1 | 49.3 | 5.6 | 2.5 | 2.9 | **61.2** | **3.7** |
|  | U42* | *3.0* | *20.2* | 8.7 | 6.4 | 8.2 | 4.1 | 3.1 | 3.6 | **7.8** | **3.6** | *2.4* | *8.0* | 3.8 | 3.0 | 3.6 | 2.7 | 2.4 | 2.6 | **3.5** | **2.6** |
|  | Hd1 | *6.5* | *283.8* | 230.1 | 231.4 | 164.5 | 18.6 | 4.8 | 6.3 | **208.7** | **9.9** | *4.9* | *168.2* | 135.3 | 136.0 | 96.9 | 12.0 | 3.8 | 4.8 | **122.7** | **6.9** |
|  | Hd31-34 | *2.7* | *945.5* | 1,222.1 | 914.3 | 819.4 | 31.2 | 2.7 | 3.2 | **985.2** | **12.3** | *2.5* | *241.9* | 309.4 | 231.5 | 207.9 | 9.8 | 2.3 | 2.5 | **249.6** | **4.9** |
| Scaffold-7 | U38* | *2.4* | *30.5* | 12.0 | 7.7 | 9.9 | 4.1 | 2.3 | 3.3 | **9.9** | **3.2** | *2.3* | *14.8* | 5.8 | 4.4 | 5.1 | 3.1 | 2.4 | 2.6 | **5.1** | **2.7** |
|  | Hd27 | *2.8* | *2,407.8* | 2,776.9 | 2,225.4 | 2,038.9 | 60.6 | 2.9 | 3.4 | **2,347.1** | **22.3** | *2.4* | *479.4* | 546.6 | 437.5 | 401.8 | 14.2 | 2.4 | 2.6 | **462.0** | **6.4** |
|  | Hd52* | *2.5* | *141.2* | 183.8 | 134.3 | 120.1 | 7.2 | 2.7 | 3.3 | **146.1** | **4.4** | *2.3* | *35.4* | 43.3 | 32.0 | 29.0 | 3.6 | 2.5 | 2.7 | **34.8** | **2.9** |
|  | Hd5 | *2.5* | *219.7* | 193.1 | 169.6 | 139.5 | 10.6 | 2.5 | 3.0 | **167.4** | **5.4** | *2.5* | *124.4* | 106.6 | 93.5 | 77.6 | 7.3 | 2.5 | 2.8 | **92.6** | **4.2** |
|  | Hd47 | *2.6* | *248.2* | 306.3 | 260.2 | 217.6 | 10.5 | 2.6 | 3.0 | **261.4** | **5.4** | *2.4* | *67.3* | 80.2 | 68.0 | 57.5 | 4.5 | 2.4 | 2.5 | **68.6** | **3.1** |
|  | IVSPER-5 | *2.7* | *21.5* | 9.6 | 6.5 | 7.7 | 4.0 | 2.7 | 3.2 | **7.9** | **3.3** | *2.4* | *9.2* | 4.2 | 3.3 | 4.1 | 2.9 | 2.4 | 2.6 | **3.9** | **2.6** |
|  | IVSPER-3 | *2.5* | *342.0* | 130.7 | 101.5 | 121.2 | 17.9 | 2.6 | 3.0 | **117.8** | **7.8** | *2.4* | *220.5* | 83.8 | 66.1 | 78.4 | 12.6 | 2.5 | 2.8 | **76.1** | **6.0** |
|  | U39* | *3.4* | *91.6* | 34.2 | 24.1 | 29.6 | 8.2 | 3.5 | 4.0 | **29.3** | **5.3** | *2.5* | *33.4* | 13.3 | 10.1 | 11.9 | 4.6 | 2.7 | 2.9 | **11.8** | **3.4** |
|  | IVSPER-4 | *2.5* | *61.4* | 25.6 | 18.5 | 22.6 | 6.5 | 2.5 | 2.9 | **22.2** | **4.0** | *2.6* | *33.1* | 14.1 | 10.6 | 12.7 | 4.7 | 2.5 | 2.8 | **12.5** | **3.4** |
|  | Hd7 | *2.5* | *156.7* | 101.3 | 93.3 | 87.5 | 5.9 | 2.5 | 2.8 | **94.0** | **3.7** | *2.5* | *98.0* | 63.5 | 58.5 | 55.1 | 4.6 | 2.5 | 2.6 | **59.0** | **3.2** |
|  | Hd2 | *2.6* | *373.3* | 334.6 | 296.2 | 241.6 | 17.5 | 2.7 | 3.4 | **290.8** | **7.9** | *2.5* | *204.7* | 180.0 | 159.5 | 130.6 | 10.8 | 2.6 | 2.9 | **156.7** | **5.4** |
|  | Hd6 | *3.4* | *473.3* | 273.7 | 262.2 | 217.4 | 12.7 | 2.6 | 2.9 | **251.1** | **6.1** | *3.0* | *253.8* | 145.2 | 138.8 | 115.7 | 7.9 | 2.4 | 2.6 | **133.2** | **4.3** |
|  | Hd2-like | *3.5* | *527.9* | 551.9 | 536.2 | 380.8 | 32.4 | 3.9 | 5.1 | **489.6** | **13.8** | *ND* | *ND* |  |  |  |  |  |  |  |  |
| Scaffold-8 | Hd20 | *2.4* | *189.2* | 196.2 | 162.9 | 139.6 | 9.0 | 2.5 | 3.1 | **166.2** | **4.9** | *2.3* | *64.6* | 64.4 | 53.6 | 46.4 | 4.8 | 2.4 | 2.5 | **54.8** | **3.2** |
|  | Hd8 | *2.4* | *404.1* | 343.0 | 288.1 | 248.5 | 12.1 | 2.7 | 3.0 | **293.2** | **5.9** | *2.3* | *155.5* | 129.8 | 108.9 | 94.4 | 6.2 | 2.3 | 2.5 | **111.1** | **3.7** |
|  | Hd4 | *2.2* | *779.6* | 617.8 | 607.2 | 477.5 | 21.2 | 2.4 | 2.9 | **567.5** | **8.9** | *2.2* | *339.8* | 267.4 | 262.0 | 206.7 | 10.8 | 2.4 | 2.7 | **245.4** | **5.3** |
|  | Hd17 | *2.3* | *342.2* | 400.3 | 354.7 | 290.4 | 13.0 | 2.5 | 3.1 | **348.5** | **6.2** | *2.2* | *138.3* | 159.1 | 140.9 | 115.8 | 6.6 | 2.3 | 2.5 | **138.6** | **3.8** |
|  | Hd54* | *2.4* | *318.7* | 305.6 | 301.8 | 235.4 | 12.0 | 2.6 | 3.0 | **280.9** | **5.9** | *2.3* | *103.7* | 97.3 | 95.3 | 75.2 | 5.7 | 2.5 | 2.6 | **89.3** | **3.6** |
|  | Hd18 | *2.5* | *770.9* | 895.6 | 784.1 | 650.1 | 26.6 | 2.8 | 2.9 | **776.6** | **10.8** | *2.3* | *229.3* | 262.8 | 229.7 | 191.1 | 9.7 | 2.5 | 2.5 | **227.8** | **4.9** |
| Scaffold-9 | U40* | *2.6* | *36.8* | 16.4 | 11.1 | 13.9 | 5.1 | 2.8 | 3.3 | **13.8** | **3.7** | *2.4* | *36.9* | 37.9 | 27.7 | 26.5 | 4.1 | 2.5 | 2.6 | **30.7** | **3.1** |
|  | Hd39 | *2.4* | *242.9* | 334.5 | 239.1 | 218.9 | 11.6 | 2.7 | 3.2 | **264.2** | **5.9** |  |  |  |  |  |  |  |  |  |  |
|  | U41* | *2.4* | *21.7* | 10.6 | 6.8 | 8.8 | 3.8 | 2.6 | 2.7 | **8.7** | **3.0** | *2.2* | *10.8* | 5.2 | 3.8 | 4.5 | 2.9 | 2.4 | 2.4 | **4.5** | **2.6** |
| Scaffold-10 | Hd23.2 | *2.8* | *90.1* | 81.0 | 63.3 | 59.6 | 5.2 | 2.6 | 3.0 | **68.0** | **3.6** | *2.6* | *26.8* | 20.9 | 15.9 | 15.7 | 3.5 | 2.6 | 2.8 | **17.5** | **3.0** |
|  | Hd23.1 | *2.5* | *229.2* | 290.0 | 254.2 | 223.9 | 10.2 | 2.5 | 2.8 | **256.0** | **5.2** | *2.4* | *63.0* | 75.6 | 66.1 | 58.9 | 4.7 | 2.5 | 2.5 | **66.9** | **3.2** |
|  | Hd25 | *2.5* | *496.3* | 530.4 | 448.8 | 389.6 | 13.3 | 2.5 | 2.9 | **456.3** | **6.2** | *2.4* | *117.4* | 122.9 | 103.6 | 90.5 | 5.0 | 2.3 | 2.5 | **105.7** | **3.3** |
| Scaffold-11 | Hd10 | *2.4* | *403.5* | 326.7 | 309.2 | 263.0 | 13.5 | 2.6 | 3.0 | **299.6** | **6.3** | *2.3* | *154.0* | 123.0 | 116.1 | 99.3 | 6.7 | 2.4 | 2.5 | **112.8** | **3.9** |
|  | Hd11 | *2.3* | *360.6* | 353.8 | 304.8 | 243.8 | 13.2 | 2.6 | 3.1 | **300.8** | **6.3** | *3.8* | *174.9* | 168.4 | 144.8 | 116.4 | 7.7 | 2.4 | 2.6 | **143.2** | **4.2** |
|  | Hd16 | *2.5* | *1,298.3* | 1,550.1 | 1,331.3 | 1,102.1 | 45.6 | 2.6 | 3.1 | **1,327.8** | **17.1** | *2.3* | *317.7* | 370.2 | 323.5 | 267.3 | 13.3 | 2.4 | 2.6 | **320.3** | **6.1** |
|  | Hd12 | *2.3* | *176.9* | 193.1 | 200.3 | 157.5 | 9.4 | 2.6 | 3.0 | **183.7** | **5.0** |  |  |  |  |  |  |  |  |  |  |
|  | Hd29 | *2.6* | *587.4* | 746.8 | 574.9 | 498.9 | 23.4 | 2.7 | 2.9 | **606.9** | **9.7** | *2.4* | *152.7* | 114.2 | 90.0 | 86.0 | 9.8 | 2.4 | 2.8 | **96.7** | **5.0** |
|  | IVSPER-2 | *2.5* | *153.4* | 60.2 | 47.7 | 55.2 | 10.9 | 2.5 | 2.9 | **54.4** | **5.4** |  |  |  |  |  |  |  |  |  |  |
|  | Hd24 | *2.6* | *195.7* | 219.8 | 181.4 | 156.9 | 11.0 | 2.5 | 3.1 | **186.1** | **5.5** |  |  |  |  |  |  |  |  |  |  |
|  | Hd33 | *2.6* | *243.6* | 353.1 | 282.9 | 239.7 | 10.2 | 2.6 | 3.2 | **291.9** | **5.3** | *2.4* | *114.6* | 93.4 | 77.0 | 70.4 | 8.4 | 2.5 | 2.8 | **80.3** | **4.6** |
|  | Hd15 | *2.6* | *363.5* | 396.2 | 350.7 | 294.9 | 22.5 | 2.7 | 3.2 | **347.2** | **9.5** |  |  |  |  |  |  |  |  |  |  |
|  | IVSPER-1 | *2.5* | *141.1* | 58.0 | 41.3 | 48.8 | 10.3 | 2.6 | 2.9 | **49.4** | **5.3** |  |  |  |  |  |  |  |  |  |  |
|  | Hd14 | *2.3* | *325.1* | 359.4 | 336.4 | 276.4 | 10.6 | 2.5 | 3.1 | **324.0** | **5.4** | *2.2* | *107.1* | 115.5 | 107.8 | 89.1 | 5.1 | 2.4 | 2.5 | **104.1** | **3.3** |
|  | Hd32 | *2.2* | *401.6* | 465.2 | 392.1 | 323.5 | 15.0 | 2.6 | 2.9 | **393.6** | **6.9** | *2.2* | *200.3* | 229.7 | 193.6 | 160.2 | 8.9 | 2.5 | 2.5 | **194.5** | **4.6** |
|  | Hd42 | *2.4* | *184.4* | 225.3 | 169.0 | 139.4 | 6.3 | 2.7 | 3.0 | **177.9** | **4.0** | *2.2* | *48.5* | 57.2 | 43.2 | 36.0 | 3.4 | 2.4 | 2.4 | **45.5** | **2.7** |
|  | Hd21 | *2.4* | *311.7* | 371.9 | 292.6 | 278.7 | 14.6 | 2.6 | 3.1 | **314.4** | **6.8** | *2.3* | *75.0* | 85.4 | 67.0 | 64.4 | 5.4 | 2.4 | 2.7 | **72.3** | **3.5** |
|  | Hd53* | *2.8* | *178.2* | 164.1 | 159.5 | 118.2 | 7.6 | 2.7 | 2.9 | **147.3** | **4.4** | *2.5* | *49.1* | 43.2 | 41.4 | 31.5 | 3.9 | 2.5 | 2.6 | **38.7** | **3.0** |
|  | Hd3 | *2.3* | *806.7* | 927.5 | 919.1 | 727.2 | 33.5 | 2.6 | 2.8 | **857.9** | **13.0** | *2.3* | *366.6* | 415.3 | 410.7 | 325.8 | 16.5 | 2.4 | 2.4 | **383.9** | **7.1** |
|  | Hd37 | *2.2* | *220.0* | 333.9 | 245.9 | 232.9 | 11.0 | 2.5 | 2.9 | **270.9** | **5.5** | *2.1* | *55.0* | 79.4 | 58.7 | 55.9 | 4.4 | 2.3 | 2.2 | **64.7** | **3.0** |
| Scaffold-12 | Hd28 | *2.5* | *40.1* | 41.7 | 30.8 | 26.9 | 3.7 | 2.7 | 3.1 | **33.1** | **3.2** | *2.4* | *19.0* | 19.1 | 14.4 | 12.8 | 3.0 | 2.6 | 2.7 | **15.4** | **2.8** |
|  | Hd35 | *2.9* | *1,062.7* | 1,174.6 | 923.3 | 856.8 | 33.4 | 3.8 | 5.0 | **984.9** | **14.0** | *2.4* | *219.0* | 239.9 | 190.8 | 180.2 | 9.2 | 2.6 | 2.9 | **203.6** | **4.9** |
