# Supplementary material for: Identification of a viral gene essential for the genome replication of a domesticated endogenous virus in ichneumonid parasitoid wasps
Source: PLoS Pathog. 2024 Apr 25;20(4):e1011980. doi: 10.1371/journal.ppat.1011980 (PMC11075835; doi:10.1371/journal.ppat.1011980)
Supplement: S4 Table — (DOCX) [file ppat.1011980.s007.docx]

**S4 Table. List of primers used in the present work.**

| **Primer name** | **Primer sequence** | **Target gene/fragment** |  |
| --- | --- | --- | --- |
| ds-RNA synthesis | | | |
| ds-U16 F | ACA-AAT-GGA-AAG-GCG-ATA-CG | *U16* |  |
| ds-U16 R | CGC-ATA-TCT-TCC-GCG-TTA-TT |  |  |
| qPCR on gDNA and cDNA | | | |
| Wasp genes | | | |
| ELF1 F | AGA-TCG-ACG-AGG-AAG-AAC | *elongation factor ELF 1* | normalizing gene |
| ELF1 R | AAG-CGA-GTT-GGT-TGG-GTT-TG |  |  |
| q-H1 F | AAA-ATA-GCG-CTC-CGA-CAA-CG | *histone H1* | Fig 8 |
| q-H1 R | TTG-CAA-GGA-AGA-GCC-ATT- GC |  |  |
| q-rpl55 F2 | CCC-GGG-CTC-CTG-TAT-AGA-GA | *ribosomal protein rpl55* | Fig 8 |
| q rpl55 R2 | TAT-CCG-CCT-CCG-ACA-AAG-TG |  |  |
| q-XRCC1 F | GCC-CCA-ATA-AGG-ATT-CTG-GC | *XRCC-1* | Fig 2A, 8 |
| q-XRCC1 R | TCG-TCT-CTT-GAT-CCG-CTA-CC |  |  |
| IV replication genes | | | |
| U1 F | CGT-CTG-ATA-GAA-CGC-GAC-AA | *U1* | Fig 2A |
| U1 L | GGC-CCA-ATT-TCG-GAA-TAT-CT |  |  |
| q-p53-2 F | GTT-CGA-TCA-AGC-GTT-GGT-TT | *p53-2* | Fig 2A, 8, 9A |
| q-p53-2 R | TGA-TTT-CAT-TCT-CGG-GCT-TT |  |  |
| q-IVSP2-2 F | GGG-TAA-CCA-GGA-AGT-CGT-CA | *IVSP2-2* | Fig 2A, 9A |
| q-IVSP2-2 R | TGC-AAC-GTC-CAC-TCT-CAG-AC |  |  |
| U11 F | GCA-CCA-CGT-GTA-CAG-CAA-TC | *U11* | Fig 2A, 8 |
| U11 R | TCG-TTT-GTA-CAG-GTC-GCT-TG |  |  |
| q-IVSP3-1 F | AAA-CAT-CGG-CGG-TAA-ATG-AG | *IVSP3-1* | Fig 2A, 8, 9A |
| q-IVSP3-1 R | CCC-TCA-GAT-CGT-TCT-TCT-CG |  |  |
| q-IVSP4-1 F | GGC-TTC-AAA-CTT-TCC-GAT-GA | *IVSP4-1* | Fig 9A |
| q-IVSP4-1 R | ATG-CGA-TGA-TT-TCC-GAG-TC |  |  |
| U6 F | GTC-TGT-CTT-TCA-CGC-AGC-AA | *U6* | Fig 2A |
| U6 R | CCT-GCG-ATC-CAC-ATT-TTC-TT |  |  |
| U15 F | GCT-TGT-CGG-ACG-AAA-ATA-GC | *U15* | Fig 2A |
| U15 R | TGA-GTG-GTT-ACG-CAT-GGT-GT |  |  |
| q-p12-1 F | CCA-GTC-TTG-TGA-GTG-GTG-GA | *p12-1* | Fig 2A, 8, 9A |
| q-p12-1 R | TTT-CGA-CGA-AGA-GTC-ATC-AGA |  |  |
| q-U22 F | TGG-GCT-CAT-GTT-GGT-ATT-CA | *U22* | Fig 2A, 8, 9A |
| q-U22 R | GGT-GCG-GTT-CAT-GGA-TTT-AG |  |  |
| q-U23 F | AGA-TTC-TGC-TGC-CCT-TGA-TG | *U23* | Fig 2A, 8, 9A |
| q-U23 R | TAC-GAC-AGA-TCC-GAC-TGA-GC |  |  |
| q-p53-1 F | ATG-CCA-CGG-AAG-CAA-TAA-TC | *p53-1* | Fig 2A, 8, 9A |
| q-p53-1 R | GTG-ATA-AGA-TCG-CGG-ATG-GT |  |  |
| q-U16 F | CCT-ACA-AAC-CCC-AGA-TTG-GA | *U16* | Fig 6A, 8, 9A |
| q-U16 R | TCT-CCA-GTA-GCT-TGC-CGA-TT |  |  |
| q-U34 F | CAA-CGT-GTT-CTG-GGA-CAT-TG | *U34* | Fig 8 |
| q-U34 R | ACA-ACA-ATC-CAG-CAC-CAT-GA |  |  |
| U36 F | CGA-GGT-GTG-CAA-CAG-CTA-TG | *U36* | Fig 8 |
| U36 R | CAA-TGT-CAC-TTA-CGC-GAG-GA |  |  |
| U37 F | ATC-GAC-TCG-CGG-TAA-TCA-AC | *U37* | Fig 8 |
| U37 R | GCT-GAA-GAA-CGG-CAA-AAC-TC |  |  |
| Viral segment genes | | | |
| 1803 F | GCC-GTA-CGA-AAT-GGC-ATA-CA | *Vank1_Hd24* | Fig 8 |
| 1803 R | AGG-ATG-TCC-ATG-ATA-CGG-TGG |  |  |
| 1814 F | ACC-TGA-ATT-GGC-AAG-ACG-GA | *Vank1_Hd43* | Fig 8 |
| 1814 R | CGG-TTG-TAT-GAA-GCA-CGG-TGT |  |  |
| CR1 F | TCA-CCG-TAC-TCC-ATC-TGG-CA | *Vank1_Hd28* | Fig 8 |
| CR1 R | ATT-GCA-CCA-GTT-CGT-GAT-CG |  |  |
| M24intron F | AGA-TTC-TGC-TGC-CCT-TGA-TG | *M24 (Gly-Pro rich)* | Fig 8 |
| M24intron R | TAC-GAC-AGA-TCC-GAC-TGA-GC |  |  |
| q-P30 F | CTA-GTG-CAG-GCG-CGA-TCA-C | *P30_Hd6* | Fig 8 |
| q-P30 R | CCC-CAC-ACA-TCG-GGA-TAT-TTC |  |  |
| Hd29 forms | | | |
| HdBR-LR | TGC-CGA-ACG-ACG-AGA-AAG-AA | Hd29 proviral (left) | Fig 9B |
| HdBR-LL2 | ACG-AAT-GTT-CTC-GAC-GGA-CA |  |  |
| HdBR-RR | GCG-AAA-TGT-TCT-CGA-GCG-AC | Hd29 proviral (right) | Fig 9B |
| HdBR-RL | TTT-GAT-GGA-GGG-ACG-TAG-CG |  |  |
| HdBR-LL2 | ACG-AAT-GTT-CTC-GAC-GGA-CA | Hd29 episomal | Fig 9B |
| HdBR-RR | GCG-AAA-TGT-TCT-CGA-GCG-AC |  |  |
| Seg29 L | TGT-TCT-TGG-TGA-GCA-GCA-TC | Hd29 proviral + episomal | Fig 9B |
| Seg29 R | GCT-GTG-CAC-TGG-AAA-TAG-CA |  |  |
