# Supplementary material for: Identification of a viral gene essential for the genome replication of a domesticated endogenous virus in ichneumonid parasitoid wasps
Source: PLoS Pathog. 2024 Apr 25;20(4):e1011980. doi: 10.1371/journal.ppat.1011980 (PMC11075835; doi:10.1371/journal.ppat.1011980)
Supplement: S1 Fig — (DOCX) [file ppat.1011980.s008.docx]

**S1 Fig**. **DNA amplification patterns of HdIV loci in calyx cells of *Hyposoter didymator*.** Amplification curves correspond to the ratio between Counts per Million (CPM) values, calculated for 10 bp intervals, obtained for pupal stage 3 and pupal stage 1 (ratio Stage 3 / Stage 1). The region represented in the figure corresponds, for each HdIV locus, to the annotated proviral locus indicated in red, plus 10,000 bp on each side.

For proviral segments, the annotated locus corresponds to the sequence delimited on each side by direct repeats (DRs), enabling homologous recombination for circularization of the viral segment.

For IVSPERs, the annotated locus corresponds to the sequence from the start to the stop codons of the first and the last gene in the cluster. For IV replication genes located outside an IVSPER, it corresponds to the length of the CDS.

**
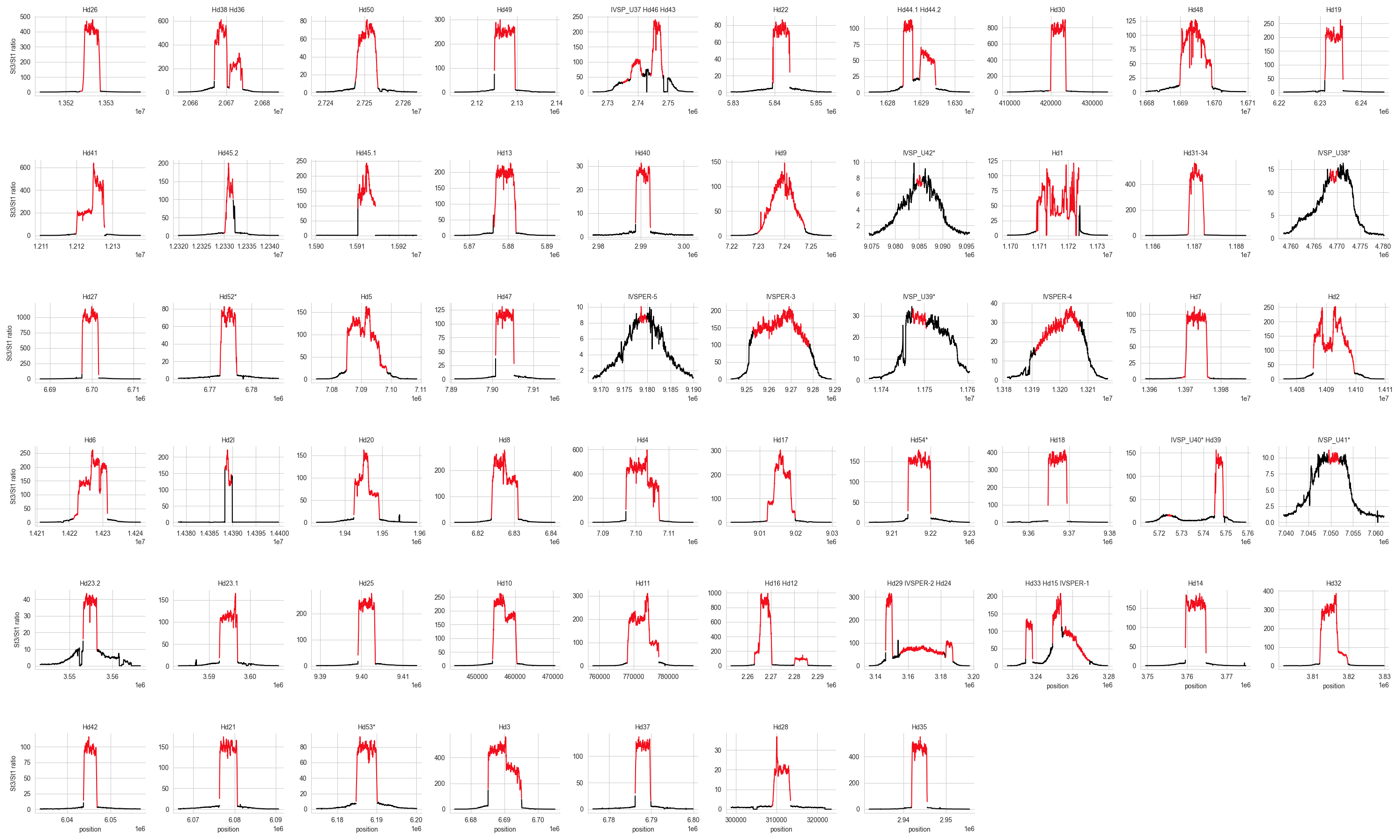
**

Scaffold-9

Scaffold-8

Scaffold-12

Scaffold-11

Scaffold-10

Scaffold-7

Scaffold-6

Scaffold-5

Scaffold-4

Scaffold-3

Scaffold-2

Scaffold-1
