# Supplementary material for: Identification of a viral gene essential for the genome replication of a domesticated endogenous virus in ichneumonid parasitoid wasps
Source: PLoS Pathog. 2024 Apr 25;20(4):e1011980. doi: 10.1371/journal.ppat.1011980 (PMC11075835; doi:10.1371/journal.ppat.1011980)
Supplement: S2 Fig — (DOCX) [file ppat.1011980.s009.docx]

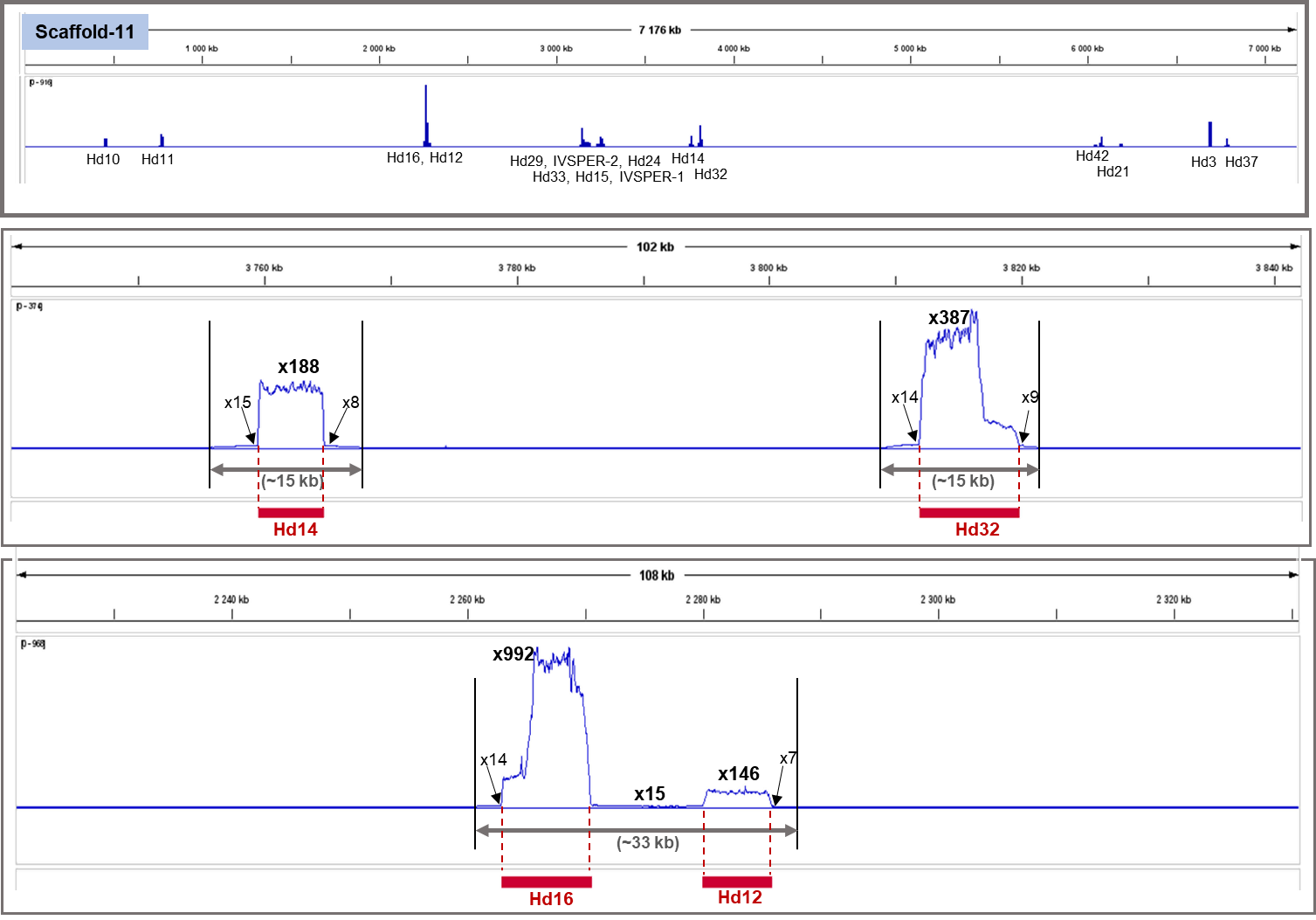
**S2 Fig. HdIV amplified regions in Scaffold-11.** Two of these regions are proviral segment loci (Hd12 and Hd14) delimited by one pair of flanking direct repeats (DRs), while two other are proviral loci (Hd32 and Hd16) that are delimited by flanking DRs but also contain internal DRs. The upper panel illustrates the amplification curve (CPM ratio stage 3 pupa / stage 1 pupa) of Scaffold-11, revealing peaks corresponding to the amplified HdIV loci. The two lower panels provide zoomed-in views of the regions of interest. The proviral segments are indicated by red bars, and the amplified regions are marked by gray arrows. For each locus, amplification values at the summit of the peaks and at the start and end positions of the annotated proviral segment loci are indicated. Each figure was generated by Integrated Genome Viewer (IGV) [1].

Reference: Robinson JT, Thorvaldsdóttir H, Winckler W, Guttman M, Lander ES, Getz G, Mesirov JP. Integrative Genomics Viewer. Nat Biotechnol. 2011;29:24-26. doi:10.1038/nbt.1754.
