# Supplementary material for: Identification of a viral gene essential for the genome replication of a domesticated endogenous virus in ichneumonid parasitoid wasps
Source: PLoS Pathog. 2024 Apr 25;20(4):e1011980. doi: 10.1371/journal.ppat.1011980 (PMC11075835; doi:10.1371/journal.ppat.1011980)

**S3 Fig. MEME analysis of boundaries of the predicted MACS2 HdIV amplified regions.**

Analyses were conducted using the MEME suite (https://meme-suite.org/meme/tools/meme).

**(A)** MEME output using 110 sequences, each consisting of 1,000 bp on either side of the start and end positions of the 55 HdIV amplified regions predicted by the MACS2 algorithm.


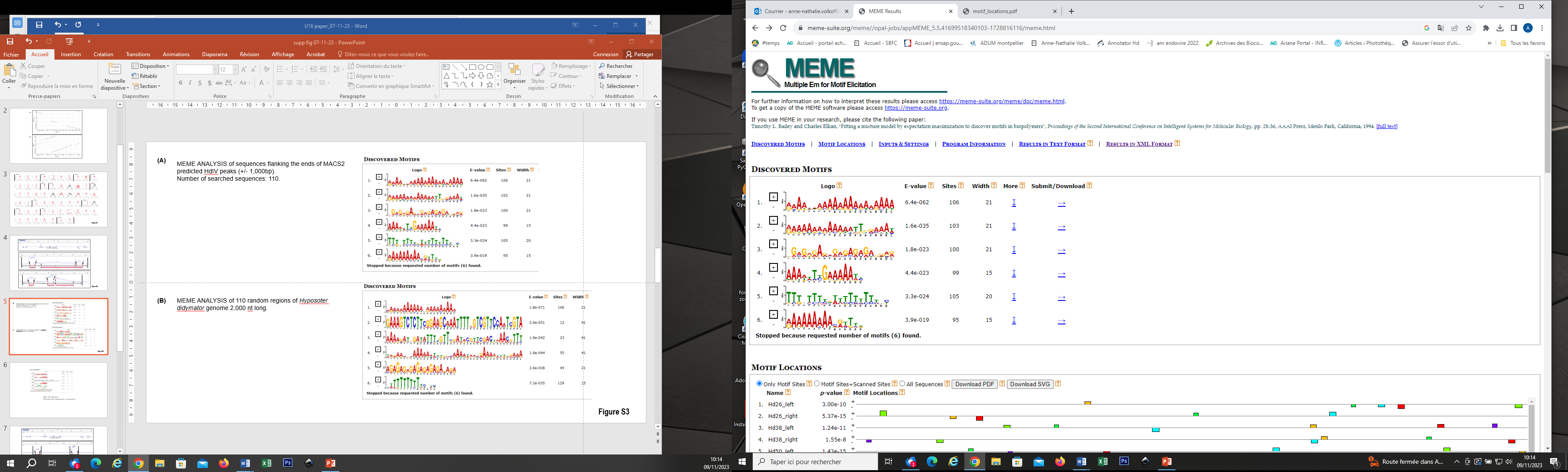


**(B)** MEME output using 110 sequences, each 2,000 bp long, randomly selected from the *H. didymator* genome outside the proviral loci.


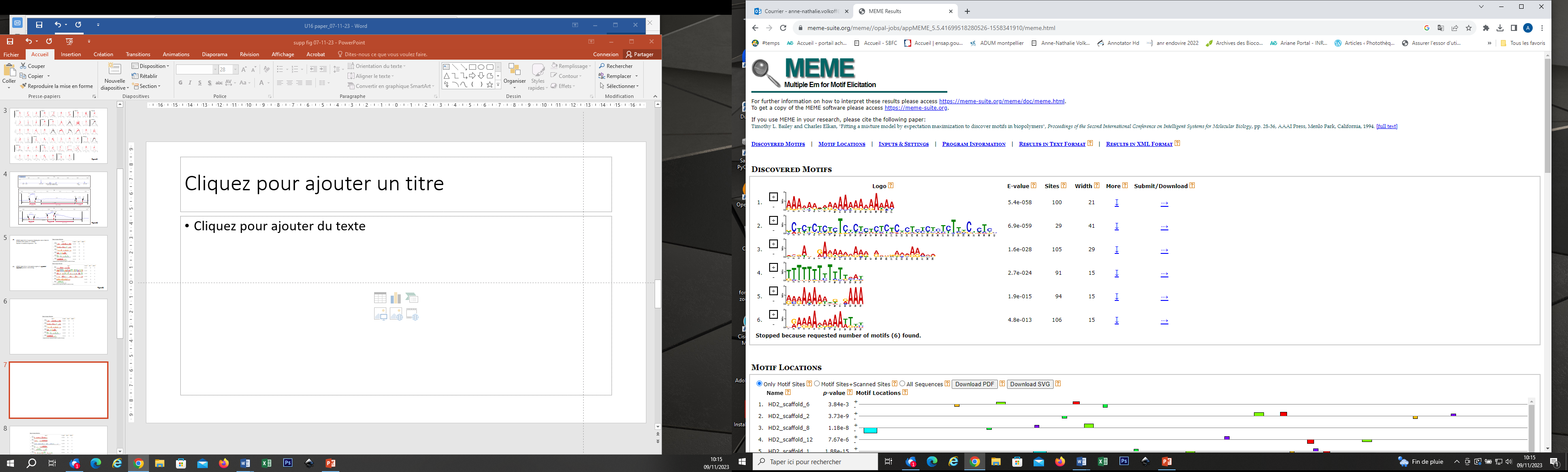

Supplement: S3 Fig — (DOCX) [file ppat.1011980.s010.docx]
